# Widespread Endogenization of Genome Sequences of Non-Retroviral RNA Viruses into Plant Genomes

**DOI:** 10.1371/journal.ppat.1002146

**Published:** 2011-07-14

**Authors:** Sotaro Chiba, Hideki Kondo, Akio Tani, Daisuke Saisho, Wataru Sakamoto, Satoko Kanematsu, Nobuhiro Suzuki

**Affiliations:** 1 Institute of Plant Science and Resources, Okayama University, Kurashiki, Japan; 2 National Institute of Fruit Tree Science, National Agricultural Research Organization (NARO), Morioka, Japan; University of Kentucky, United States of America

## Abstract

Non-retroviral RNA virus sequences (NRVSs) have been found in the chromosomes of vertebrates and fungi, but not plants. Here we report similarly endogenized NRVSs derived from plus-, negative-, and double-stranded RNA viruses in plant chromosomes. These sequences were found by searching public genomic sequence databases, and, importantly, most NRVSs were subsequently detected by direct molecular analyses of plant DNAs. The most widespread NRVSs were related to the coat protein (CP) genes of the family *Partitiviridae* which have bisegmented dsRNA genomes, and included plant- and fungus-infecting members. The CP of a novel fungal virus (Rosellinia necatrix partitivirus 2, RnPV2) had the greatest sequence similarity to *Arabidopsis thaliana* ILR2, which is thought to regulate the activities of the phytohormone auxin, indole-3-acetic acid (IAA). Furthermore, partitivirus CP-like sequences much more closely related to plant partitiviruses than to RnPV2 were identified in a wide range of plant species. In addition, the nucleocapsid protein genes of cytorhabdoviruses and varicosaviruses were found in species of over 9 plant families, including Brassicaceae and Solanaceae. A replicase-like sequence of a betaflexivirus was identified in the cucumber genome. The pattern of occurrence of NRVSs and the phylogenetic analyses of NRVSs and related viruses indicate that multiple independent integrations into many plant lineages may have occurred. For example, one of the NRVSs was retained in *Ar. thaliana* but not in *Ar. lyrata* or other related Camelina species, whereas another NRVS displayed the reverse pattern. Our study has shown that single- and double-stranded RNA viral sequences are widespread in plant genomes, and shows the potential of genome integrated NRVSs to contribute to resolve unclear phylogenetic relationships of plant species.

## Author Summary

Eukaryotic genomes contain sequences that have originated from DNA viruses and reverse-transcribing viruses, i.e., retroviruses, pararetroviruses (DNA viruses), and transposons. However, the sequences of non-retroviral RNA viruses, which are unable to convert their genomes to DNA, were until recently considered not to be integrated into eukaryotic nuclear genomes. We present evidence for multiple independent events of horizontal gene transfer from a wide range of RNA viruses, including plus-sense, minus-sense, and double-stranded RNA viruses, into the genomes of distantly related plant lineages. Some non-retroviral integrated RNA viral sequences are conserved across genera within a plant family, whereas others are retained only in a limited number of species in a genus. Integration profiles of non-retroviral integrated RNA viral sequences demonstrate the potential of these sequences to serve as powerful molecular tools for deciphering phylogenetic relationships among related plants. Moreover, this study highlights plants co-opting non-retroviral RNA virus sequences, and provides insights into plant genome evolution and interplay between non-reverse-transcribing RNA viruses and their hosts.

## Introduction

Events of horizontal gene transfer (HGT) have been identified between various combinations of viruses and their eukaryotic hosts. HGT can occur during evolution in 2 inverse directions: “from host to virus” or “from virus to host.” In the host to virus direction, viral acquisition of host genes is observed as insertion of cellular genes for proteases (see [1] for review), ubiquitin [2], chloroplast protein [3] and heat-shock proteins [4], [5] into viral genomes. The virus to host direction involves endogenization of viral genes. Fossil sequences of viral origin, mostly from retroviruses, have been detected in many animal genomes. However, retrovirus sequences have not been identified in plants; instead, reverse-transcribing DNA viruses (pararetroviruses) have been identified. Although pararetroviral sequences have been found in some plant nuclear genomes [6], [7], [8], [9], only a limited number of integrated sequences are exogenized to launch virus infection; however, their cellular functions remain unclear in other examples.

In contrast, the sequences of non-retroviral RNA viruses were considered not to integrate into host chromosomes. However, recent reports identified endogenized genes of non-retroviral elements in mammals [10], [11], [12], [13]. Examples include the nucleocapsid protein (*N*) and nucleoprotein (*NP*) genes of bornaviruses and filoviruses, members of the negative-strand RNA virus group in the order *Mononegavirales* [11], [12], [14]. While some integrated *N* genes are expressed, their biological significance is unclear. Identification of these sequences contrasts with the lack of evidence for negative-strand RNA virus genome integration into plant genomes. Furthermore, RNA-dependent RNA polymerase (RdRp) and capsid protein (CP) coding domains from a group of monopartite dsRNA viruses have been identified in yeast chromosomes, and while some of these viruses appear to be expressed, their biological significance has not been explored [15], [16], [17].

The white root rot fungus *Rosellinia necatrix* is a soil-borne phytopathogenic ascomycetous fungus that causes damages to perennial crops. An extensive search of a large collection of field fungal isolates (over 1,000) was conducted to identify dsRNA (mycoviruses) that may serve as virocontrol (biological control) agents. Approximately 20% of field isolates were infected with known or unknown viral strains [18], [19], [20]. During molecular characterization of these viruses, we identified a novel partitivirus termed Rosellinia necatrix partitivirus 2 (RnPV2) in an ill-defined *R. necatrix* strain. The family *Partitiviridae* contains members with small bi-segmented dsRNA genomes [21] that infect plants, fungi or protozoa. They are thought to replicate using virion-associated RdRp in the host cytoplasm, which are phylogenetically related to those from the picorna-like superfamily [22]. Surprisingly, the RnPV2 CP showed the highest level of sequence identity to an *Arabidopsis thaliana* gene, *IAA/LEU resistant 2* (*ILR2*), which was previously shown to regulate the activity of the phytohormone auxin [23]. Combined with information regarding integrated mononegaviral sequences in animals, this finding generated significant interest in searching currently available genome sequence data for not only dsRNA but also negative-strand viral sequences. In October 2010, Liu et al. [24] reported similar results based on an extensive search conducted in 2009. This group identified sequences in the chromosomes of diverse organisms that may have been acquired from monopartite (totiviruses and related unclassified viruses) and bipartite dsRNA viruses (partitiviruses).

We further examined plant genome sequences available as of December 10, 2010 for integrated sequences of not only partitivirus genomes but also negative-, and positive-strand RNA viruses (Table S1). Combining database searches and molecular analyses led to the identification of multiple endogenized sequences related to partitiviruses, cytorhabdoviruses, varicosaviruses and betaflexiviruses in the genomes of a variety of plants including those from the families Solanaceae and Brassicaceae. For example, while some partitivirus-related sequences are conserved on the orthologous locus across some genera, e.g., *Arabidopsis*, *Capsella*, *Turritis,* and *Olimarabidopsis* within the family Brassicaceae, others are retained in only a few species within a single genus, *Arabidopsis*. A similar integration pattern was observed for a rhabdovirus-related sequence in the family Solanaceae. These profiles of occurrence can potentially resolve unclear phylogenetic relationships between plants. Our study demonstrates widespread endogenization of non-retroviral RNA virus sequences (NRVSs) including sequences of plant positive- and negative-strand RNA viruses for the first time. We have proposed a model of viral gene transfer, in which NRVSs are suggested to be a factor constituting plant genomes.

## Results

### The CP sequence from a novel mycovirus shows the highest identity to a plant functional gene product, ILR2

We determined the complete nucleotide (nt) sequence of the genome segments (dsRNA1 and dsRNA2) of a novel partitivirus, RnPV2, from the white root rot fungus *Rosellinia necatrix*, a soil-borne phytopathogenic ascomycetous fungus. DsRNA2 was found to be 1828 nt long, encoding a polypeptide of 483 amino acids (aa) (CP, 54 kDa). Low-level sequence similarities among CPs from *Partitiviridae* family members were observed using a BLASTP search with RnPV2 CP against non-redundant sequences available in the NCBI database (http://www.ncbi.nlm.nih.gov/). Surprisingly, RnPV2 CP showed the highest degree of sequence similarity to ILR2 from *Ar. thaliana*. Notably, sequence similarities between RnPV2 CP and ILR2 were greater than those between the CP sequence from another mycovirus, Sclerotinia sclerotiorum partitivirus S (SsPV-S) and ILR2 noted previously [24]. ILR2 is known to regulate indole-3-acetic acid (IAA)-amino acid conjugate sensitivity and metal transport. An *Ar. thaliana* mutant with a single amino acid substitution in *ILR2*, known as *ilr2-1*, was shown to exhibit normal root elongation in the presence of a high concentration of exogenous IAA-leucine conjugates, which represses root elongation in wild-type lines [23].

### RnPV2 CP-like sequences are conserved in some Brassicaceae spp. and *Mimulus guttatus*

**Figure 1:**
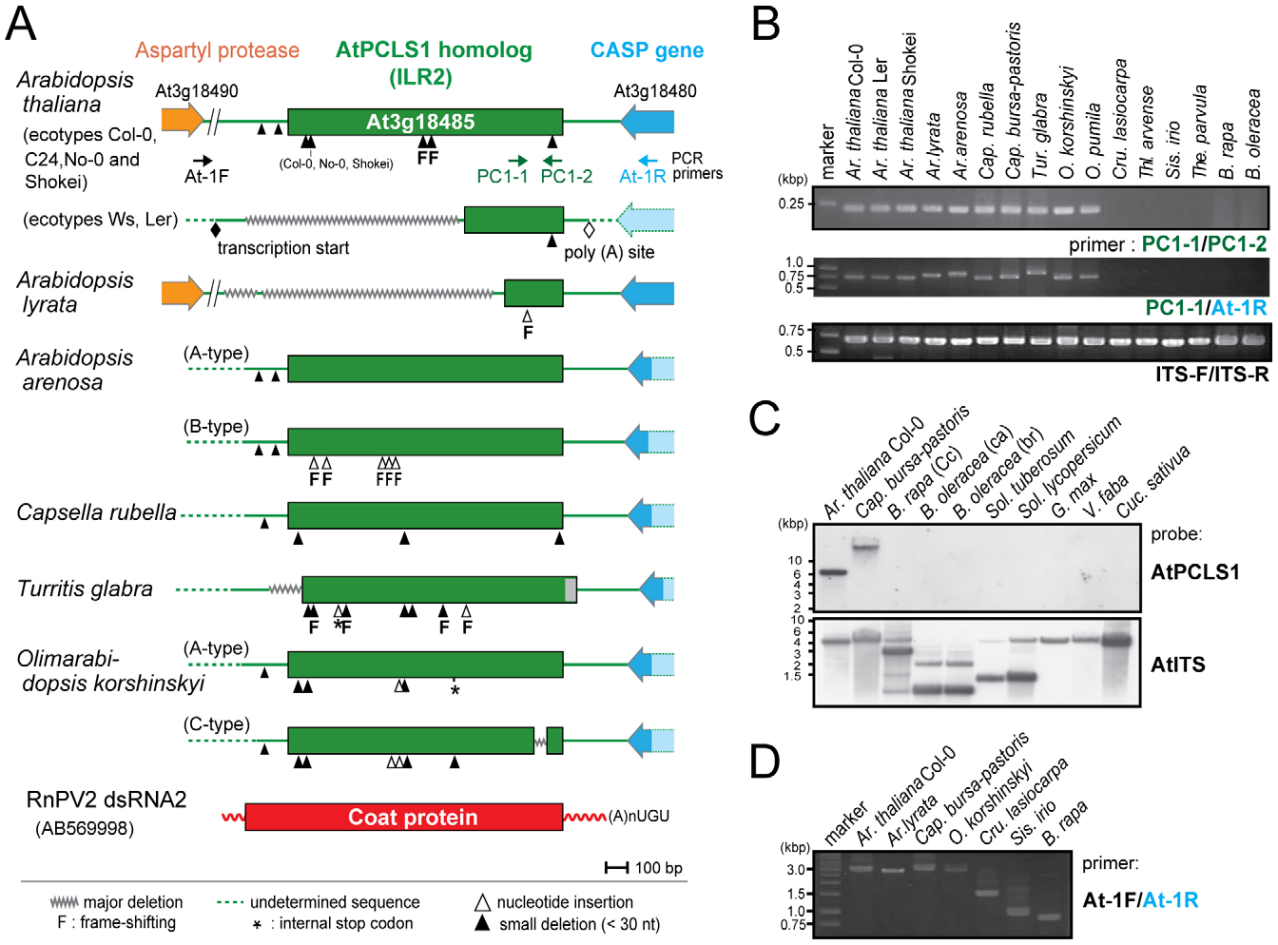
*ILR2* (*PCLS1*) homologs from members of the family Brassicaceae. (A) Schematic representation of RnPV2 CP-related ILR2 genes from *Arabidopsis*-related species. Green boxes refer to the coding regions of *ILR2* homologs, while orange and blue thick arrows indicate those of cellular genes. *Ar. thaliana* Col-0, No-0, C24 and Shokei have long versions of *ILR2*, while those of the other *Ar. thaliana* ecotypes and *Ar. lyrata* have large deletions at the 5′-terminal portion. *ILR2* homologs of *Arabidopsis* and closely related genera reside on the orthologous position. These plant homologs were most closely related to the CP gene of a fungal partitivirus, RnPV2. Symbols referring to mutations are shown at the bottom: waved line, major deletion; dashed line, undetermined sequence; open triangle, nucleotide insertion; filled triangle, small deletion (<30 nt); asterisk, internal stop codon; F, frame-shift; filled diamond, transcription start site; open diamond, poly(A) addition site. These symbols were utilized in this and subsequent figures. (B) Genomic PCR analysis of *ILR2*. The top and middle panels show amplification patterns with two primer sets (PC-1 and PC-2; PC-1 and At-1R). Primer positions and sequences are shown in Figure 1A and Table S3. A primer set, At-IRS-FW (ITS-F) and At-IRS-RV (ITS-R) [66], was used for amplification of the complete ribosomal internal transcribed spacer (ITS) regions 1 and 2 including the 5.8S rDNA. (C) Southern blotting of plant species in different families. Ten microgram of *Eco* RI-digested genomic DNA (per lane), except for that from *Ar. thaliana* Col-0 (2.5 µg/lane), was probed with a DIG-labeled *ILR2* (top panel) or ITS DNA fragment (bottom panel) derived from *Ar. thaliana* Col-0. (D) Genomic PCR analysis of the *ILR2*-flanking region. PCR fragments were amplified by a primer set (At-1F and At-1R) on *ILR2*-carrying genomic DNAs from *Ar. thaliana*, *Ar. lyrata*, *Cap. bursa-pastoris*, and *O. korshinskyi*, and *ILR2*-non-carrying DNAs from *Cru. lasiocarpa*, *Sis. irio*, and *B. rapa*.

Magidin et al. [23] identified 2 alleles of *ILR2* in *Ar. thaliana* accessions (a long and a short allele) (Figure 1A). Although the authors confirmed *ILR2* expression for only the WS ecotype (short allele), they determined that both short and long versions of *ILR2* were functional. Given the similarity between ILR2 and RnPV2 CP sequences, we hypothesized that HGT occurred between the 2 organisms. Therefore, we assessed the extent to which *ILR2* is conserved in plants. We used 3 approaches: BLAST search, genomic PCR, and Southern blot analyses. We first conducted an exhaustive BLAST (tblastn) search against genome sequence databases as described in the Materials and Methods. This search identified *ILR2* homologs in *Ar. lyrata* and *Mimulus guttatus* (yellow monkey flower), which included both short and long versions of *ILR2* homologs with modest levels of aa sequence identities (over 20%) to RnPV2 CP (Table S2, Figure 1A). Furthermore, a variety of partitivirus CP-related sequences with low-levels of aa sequence identities (approximately 20%) to RnPV2 CP were also detectable from genome sequences from other 17 plant species (Table 1). These sequences were classified into a total of 8 subgroups based on relatedness to best matched extant partitiviruses (Table 1). Their nomenclature is: AtPCLS1 (ILR2) is from *Arabidopsis thaliana* partitivirus CP-like sequence (PCLS) 1. Differently numbered PCLSs, referring to proteins potentially encoded by *PCLSs*, show the highest level of aa sequence identities to CPs encoded by different partitiviruses.

**Table 1:** Non-retroviral partitivirus CP-like sequences (*PCLSs*) identified in plant genome sequence databases.

*PCLS*	Plant	Sequence ID	Database	Best-matched virus (abbreviation, segment)	e-value	Mol. analysis^a^
*AtPCLS1*	*Arabidopsis thaliana*	At3g18485 (ILR2)^b^	NCBI	Rosellinia necatrix partitivirus 2 (RnPV2, dsRNA2)	2e-47	GP, GS, SQ, PA
*AlPCLS1*	*Arabidopsis lyrata*	929729 (XM_002885214)	Phytozome	Rosellinia necatrix partitivirus 2 (RnPV2, dsRNA2)	4e-01^c^	GP
*MgPCLS1*	*Mimulus guttatus*	mgv1a022511m.g	Phytozome	Rosellinia necatrix partitivirus 2 (RnPV2, dsRNA2)	6e-39	PA
*AtPCLS2*	*Arabidopsis thaliana*	At4g14104^b^	NCBI	Raphanus sativus cryptic virus 2 (RSCV2, dsRNA2)	3e-49	GP, SQ
*MePCLS2* d	*Manihot esculenta*	cassava4.1_029961m.g	Phytozome	Raphanus sativus cryptic virus 2 (RSCV2, dsRNA2)	3e-42	PA
*AlPCLS3*	*Arabidopsis lyrata*	352550 (XM_002872767)	Phytozome	Fragaria chiloensis cryptic virus (FCCV, dsRNA 2)	5e-38	GP, SQ
*BrPCLS4*	*Brassica rapa*	Bra021820	BRAD	carrot cryptic virus 1 (CaCV1, dsRNA2)	3e-70	GP, GS, SQ, PA
*BoPCLS4*	*Brassica oleracea*	BH939664^e^	NCBI-gss	carrot cryptic virus 1 (CaCV1, dsRNA2)	6e-47	GP, GS, SQ, PA
*BrPCLS5*	*Brassica rapa*	Bra020160^b^	BRAD	Raphanus sativus cryptic virus 1 (RSCV1, dsRNA2)	2e-130	GP, GS, SQ, PA
*BoPCLS5*	*Brassica oleracea*	FI711962.1^b^	NCBI-gss	Raphanus sativus cryptic virus 1 (RSCV1, dsRNA2)	3e-16	GP, GS, SQ, PA
*SpPCLS5*	*Solanum phureja*	unassigned (scaffold.20100818064734797543000)	PGSC	Raphanus sativus cryptic virus 1 (RSCV1, dsRNA2)	8e-122	PA
*StPCLS5*	*Solanum tuberosum*	EI814115^e^	NCBI-gss	Raphanus sativus cryptic virus 1 (RSCV1, dsRNA2)	5e-05	GP, GS, SQ, PA
*NtPCLS5-1*	*Nicotiana tabacum*	GSS^b^ (Contig-1)^f^	NCBI-gss	Raphanus sativus cryptic virus 1 (RSCV1, dsRNA2)	5e-106	GP, GS, SQ, PA
*NtPCLS5-2*	*Nicotiana tabacum*	GSS^b^ (Contig-2)^f^	NCBI-gss	Raphanus sativus cryptic virus 1 (RSCV1, dsRNA2)	2e-64	GP, GS, SQ
*NtPCLS6*	*Nicotiana tabacum*	GSS^b^ (Contig-3)^f^	NCBI-gss	Fragaria chiloensis cryptic virus (FCCV, dsRNA3)	1e-33	GP, GS, SQ
*VuPCLS6*	*Vigna unguiculata*	EI930635^c^	NCBI-gss	Fragaria chiloensis cryptic virus (FCCV, dsRNA3)	1e-05	-
*GmPCLS6*	*Glycine max*	unassigned (WGS ACUP01011070,984-1304)	NCBI-wgs	Fragaria chiloensis cryptic virus (FCCV, dsRNA3)	8e-10	-
*NtPCLS7*	*Nicotiana tabacum*	GSS^b^ (Contig-4)^f^	NCBI-gss	Raphanus sativus cryptic virus 3 (RSCV3, dsRNA2)	9e-06	GP, GS, SQ
*MtPCLS7*	*Medicago truncatula*	GSS^b^ (Contig-1)^f^	NCBI-gss	Raphanus sativus cryptic virus 3 (RSCV3, dsRNA2)	2e-17	GP, SQ
*MdPCLS7*	*Malus x domestica*	unassigned (wgs ACYM01118643, 10505-11776)	NCBI-wgs	Raphanus sativus cryptic virus 3 (RSCV3, dsRNA2)	4e-46	PA
*LjPCLS8*	*Lotus japonicus*	AP010106^e^	NCBI-htgs	rose cryptic virus (RoCV, dsRNA 3)	6e-63^g^	GP, SQ
*PdPCL8*	*Phoenix dactylifera*	unassigned (wgs ACYX01071982, 560-268; 790-1379)	NCBI-wgs	rose cryptic virus (RoCV, dsRNA 3)	2e-24	-
*SbPCL8*	*Sorghum bicolor*	unassigned (wgs ABXC01001628, 27853-28723)	NCBI-wgs	rose cryptic virus (RoCV, dsRNA 3)	1e-40	PA
*ZmPCLS8* d	*Zea mays*	GSS^b^ (Contig-1)^f^	NCBI-gss	rose cryptic virus (RoCV, dsRNA 3)	7e-09	-

a Molecular analysis carried out in this study: GP, genomic PCR; GS, genomic Southern blot; SQ, sequencing; PA, phylogenetic analysis; -, not performed.

b Reported as non-retroviral integrated plant genome sequence by Liu et al. (2010).

c AlPCLS1 shows an e-value, 3e-35 against AtILR2.

d MePCLS2 in cassava and ZmPCLS8 in maize were found in intron of particular gene loci.

e Reported as the candidates for non-retroviral integrated plant genome sequence in Liu et al. (2010).

f Contig1-4 indicate GSS assembly sequences as described by Liu et al. (2010).

g An unrelated sequence interrupting the virus-like sequence (Figure S1A) was removed for BLAST search.

Genomic PCR analysis with primers corresponding to highly conserved 240-bp portions revealed that *ILR2* homologs were retained in genera closely related to *Arabidopsis*, such as *Capsella*, *Turritis*, and *Olimarabidopsis*, but not in members of distantly-related genera, *Brassica*, *Thellungiella*, *Crucihimalaya*, *Sisymbrium*, and *Thlaspi* within the Brassicaceae family (Figure 1B). Genomic PCR fragments covering *the entire ILR2*-like domains of the plants shown in Table S4 were sequenced directly or after cloning into a plasmid. It should be noted that *PCLS1*s of closely related genera reside in an orthologous position [25], i.e., in a convergent configuration with the gene for the transmembrane Golgi matrix protein AtCASP, which shares a high degree of sequence similarity across kingdoms [26]. This notion was confirmed by genomic PCR in which a primer pair allowed detection of 0.75- to 1-kb fragments spanning the CASP gene. Previous comparative genomics studies proposed a hypothesis that the Brassicaceae genomes consist of 24 (A to X) conserved genome blocks [27]. The *ILR2* locus is on block F which is considered to be duplicated in *B. rapa*. A search against the *Brassica* database (BRAD) confirmed the absence of a *PCLS1* on the 2 *B. rapa* loci that flank the CASP gene. Southern blotting with members of the Brassicaceae, Cucurbitaceae, Solanaceae, and Leguminosae families indicated that *PCLS1* (*ILR2*) is present in *Ar. thaliana* and *Cap. bursa-pastoris*, but absent in the other plants (Figure 1C), consistent with BLAST results and genomic PCR analyses. Furthermore, the absence of *ILR2* in *Crucihimalaya lasiocarpa*, *Sisymbrium irio* and *B. rapa* was confirmed by sequence analysis of genomic PCR fragments covering the entire *ILR2* region and its flanking regions (Figure 1D).

### Prevalence of partitivirus CP-like sequences (PCLS1 to PCLS8) in plant chromosomes

**Figure 2 ppat-1002146-g002:**
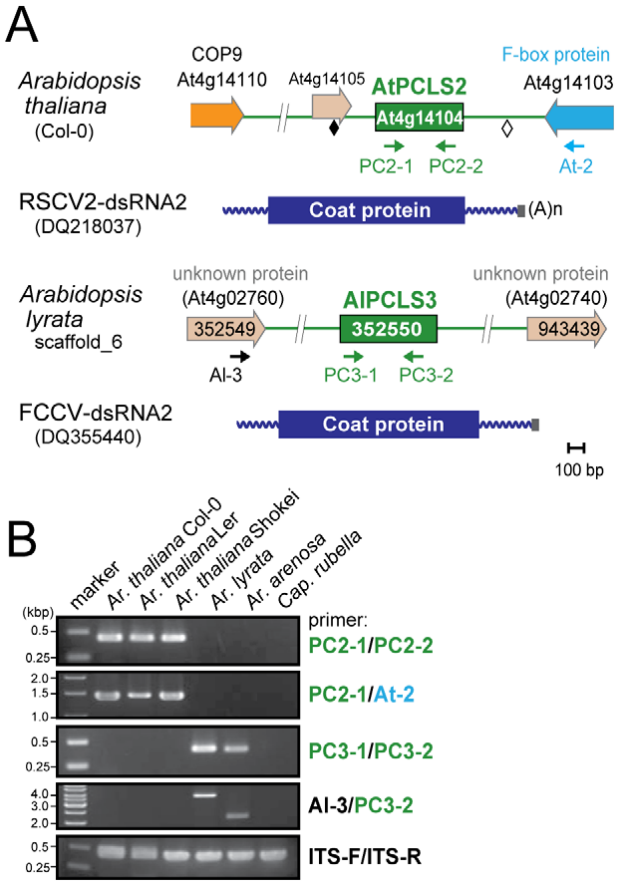
*PCLS2* and *PCLS3* homologs from members of the genus *Arabidopsis*. (A) Diagrams of the plant genome map containing *PCLS2* and *PCLS3* from *Arabidopsis*-related species. See Figure 1 legend for explanation of symbols. AtPCLS2 and AllPCL3 showed the highest levels of similarity to the CP of plant partitiviruses, Raphanus sativus cryptic virus 2 (RSCV2) and Fragaria chiloensis cryptic virus (FCCV), respectively. (B) Genomic PCR analysis of *PCLS2* and *PCLS3*. *PCLS2* homologs were amplified using primer sets PC2-1 and PC2-2 (top panel) and PC2-1 and At-2 (second panel). These primers are specific for *AtPCLS2* except for At-2, which corresponds to an F-box protein gene (At4g14103). The third and fourth panels show amplification patterns of *PCLS3* with primer sets PC3-1 and PC3-2 or Al-3 and PC3-2, respectively. A primer set, At-IRS-FW and At-IRS-RV (ITS-F and ITS-R for abbreviation, see the Figure 1 legend) were used in this and subsequent figures (Figures 3, 5, S1, S3) for amplification of the complete ITS regions. Primers' positions are shown by small arrows in A, while their sequences are shown in Table S3.

Genome sequences with low levels of similarities to RnPV2 CP included a number of PCLSs from various plants spanning more than 17 species from 8 families (Table 1). Most *PCLS*s confirmed to be present on their chromosomes of these organisms were identified by genomic PCR and/or Southern blotting and sequencing (Tables 1, S4). For instance, *AtPCLS2* and *Ar. lyrata PCLS3* (*AlPCLS3*) are retained on non-orthologous loci of *ILR2s* of *Ar. thaliana* and *Ar. lyrata*, respectively (Figure 2A). *AtPCLS2* (At4g14104) resides between the genes for COP9 (constitutive photo-morphogenic-9, *COP*9) and an F-box protein, while *AlPCLS3* is between 2 coding sequences for F-box domains corresponding to At4g02760 and At4g02740 [25]. AtPCLS2 and AlPCLS3 from 2 closely related plant species show the highest sequence identities to the CPs from 2 different partitiviruses: Raphanus sativus cryptic virus 2 (RSCV2) and Fragaria chiloensis cryptic virus (FCCV) (dsRNA2) [28]. The PCLS retention profile was revealed by genomic PCR using 2 primer sets. A primer set designed to amplify internal AtPCLS2 sequences provided DNA fragments of an expected size of 470 bp in *Ar. thaliana* accessions Col-0, Ler, and Shokei, but not in *Ar. lyrata*, *Ar. Arenosa*, or *Cap. rubella* (Figure 2B, top panel). A different primer set specific for *AtPCLS2* and the F-box protein gene (At4g14103) gave the same amplification pattern (Figure 2B, second panel) as shown in the top panel. Using the same approach with 2 sets of primers, *PCLS3* was detected by genomic PCR in *Ar. lyrata* and *Ar. arenosa*, while no such sequence was observed in *Ar. thaliana* ecotypes or *Cap. rubella* (Figure 2B, third and fourth panels). Although the COP9 and the F-box protein genes are conserved on the corresponding loci of *Ar. lyrata*, no counterpart of *AtPCLS2* was identified between the genes (Phytozome). Similarly, no *AlPCLS3* homolog was observed on the corresponding chromosomal position of *Ar. thaliana* [25].

**Figure 3:**
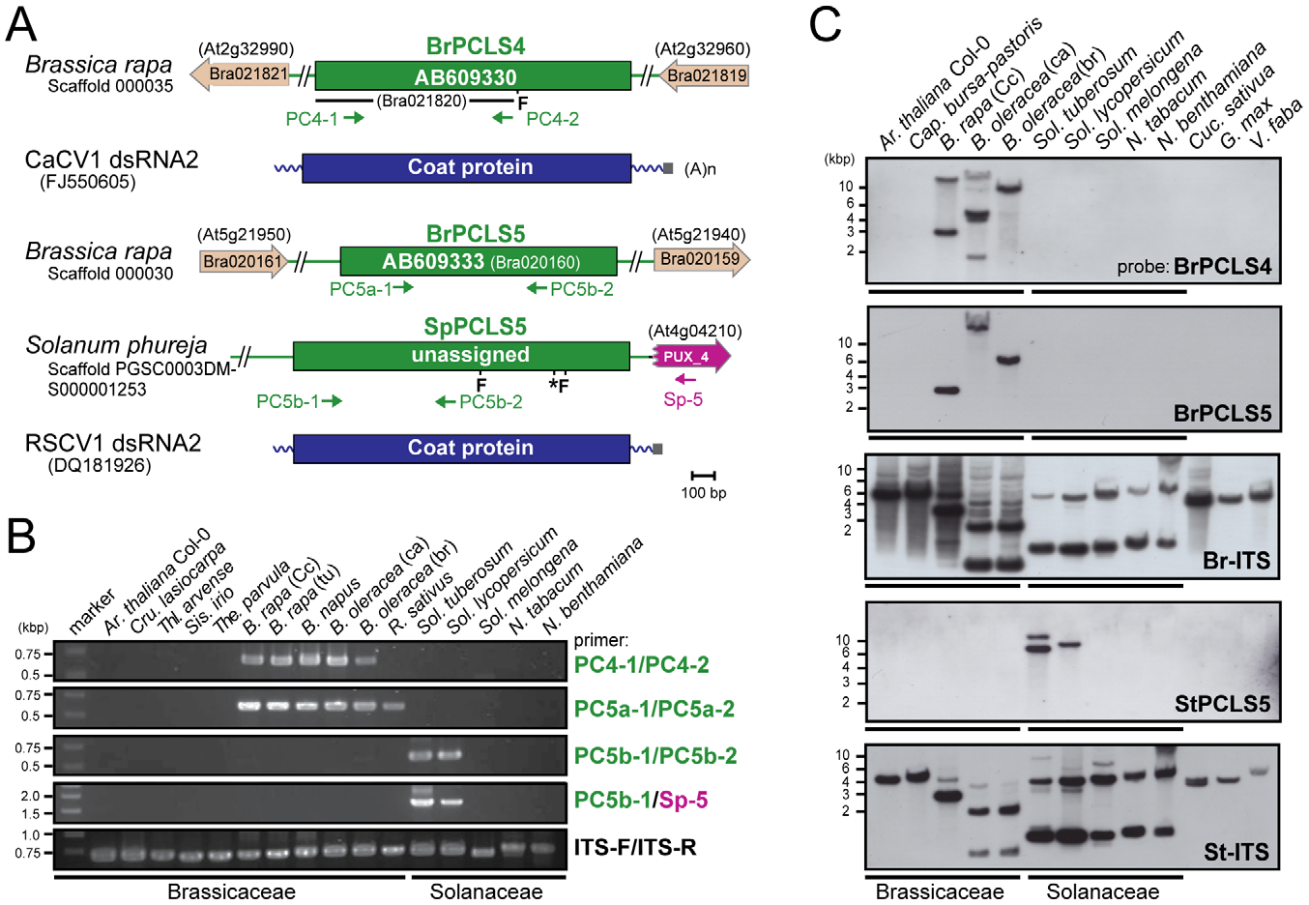
*PCLS4* and *PCLS5* homologs from members of the families Solanaceae and Brassicaceae. (*A*) Diagrams of the structures of *B. rapa PCLS4* (*BrPCLS4*), and *PCLS5s from Sol. phureja* (*SpPCLS5*) and from *B. rapa* (*BrPCLS5*). PCLS4 shows the highest similarities to carrot cryptic virus 1 (CaCV1) CP, while PCLS5s exhibit the greatest sequence similarities to the CP of another plant partitivirus, Raphanus sativus cryptic virus 1 (RSCV1). (B) Genomic PCR analysis of *PCLS4* and *PCLS5*. Genomic DNA from members of the families Brassicaceae and Solanaceae shown on the top of gels were used for amplification of *PCLS*s. Primers used were: PC4-1 and PC4-2 specific for *BrPCLS4* (top panel); PC5a-1 and PC5a-2 specific for *BrPCLS5* (second panel); PC5b-1 and PC5b-2 specific for *SpPCLS5* (third panel); PC5b-1 and SP-5 specific for *SpPCLS5* and *PUX_4* (fourth panel); ITS-F and ITS-F specific for the ITS region (bottom panel). (C) Genomic Southern blotting of *PCLS4* and *PCLS5*. *Eco*RI-digested genomic DNA isolated from various plants shown at the top of the blots were hybridized with different DIG-labeled probes specific for *BrPCLS4* (top panel), *BrPCLS5* (second panel), *B. rapa* ITS (third panel) *Sol. tuberosum PCLS5* (fourth panel) and *Sol. tuberosum* ITS (bottom panel). Migration positions of DNA size standards are shown at the left.

*PCLS4* and *PCLS5* were found in the genome sequence databases of *B. rapa* (*BrPCLS4* and *5*), *Solanum phureja* (wild species of potato) (*SpPCLS5*) (Figure 3A, S2), and *Nicotiana tabacum* (*NtPCLS5-1 and -2*) (Figure S1A). These sequences commonly exhibited greater sequence similarity to CPs of previously reported plant partitiviruses than to RnPV2 CP (Tables 1). The 3 PCLS5s from the Solanaceae family were very similar to each other (approximately 60% aa sequence identity), and showed high sequence identity (over 45%) (Table S2) to CP of Raphanus sativus cryptic virus 1 (RSCV1, plant partitivirus) [29]. Two *PCLS*s, *BrPCLS4* (Bra021820) and *BrPCLS5* (Bra020160), which are detected on different scaffolds, were determined to not flank the CASP gene of *B. rapa* as *AtPCLS1* (*ILR2*) does. BrPCLS4 and 5 show much greater aa sequence identities to CPs of RSCV1 and carrot cryptic virus 1 (CaCV1, plant partitivirus) [30] than it does to RnPV2 CP (Table S2).

Molecular analyses were performed to determine how widely these *PCLS4* and *PCLS5* are conserved. Genomic PCR using a primer set specific for *BrPCLS4* detected related sequences in all *Brassica* species tested, but not in other plants including members of the family Solanaceae or genera other than *Brassica* in Brassicaceae, such as *Ar. thaliana*, *Cru. lasiocarpa*, *Thellungiella parvula*, *Thl. arvense* and *Sis. irio*, and *Raphanus sativus* (Figure 3B, top panel). For *BrPCLS5*, the primer set, PC5a-1 and PC5a-2 enabled detection of expected PCR fragments in all *Brassica* plants in addition to *R. sativus*, while no PCR fragments were amplified in the other plant species (Figure 3B, second panels). A different detection profile was obtained by genomic PCR with a primer set specific for *SpPCLS5* in which *PCLS5*-related sequences were detectable only in *Sol. tuberosum* and *Sol. lycopersicum* (Figure 3B, third and fourth panels). We failed to yield amplification from all other tested plants in the families Brassicaceae and Solanaceae including *Sol. melongena*. Interestingly, *PCLS5*, but not *PCLS4* fragments, were detected in *R. sativus*. Moreover, the presence or absence of *PCLS*s was confirmed by genomic Southern analysis. As expected from the genomic PCR results, hybridization signals were detected with a *BrPCLS4*- or a *BrPCLS5*-specific probe in the *Brassica* species such as *B. rapa* and *B. oleracea* (Figure 3C, top and second panels); however, the numbers and signal positions differed between the 2 blots. The *StPCLS4*-specific probe allowed detection of 2 and 1 hybridization signals in *Sol. tuberosum* and *Sol. lycopersicum*, respectively, but not in any other plants examined in this study (Figure 3C, fourth panel).

In addition to *PCLS1* to *PCLS5*, 2 other subgroups of *PCLS*s (*PCLS6* and *PCLS7*) were observed in the GSS database of *N. tabacum* and showed an interesting detection pattern in *Nicotiana* species (Figure S1). NtPCLS6 and NtPCLS7 showed moderate aa sequence identities to CPs encoded by FCCV dsRNA3 (38%) [28] and RSCV3 dsRNA2 (30%) [29], respectively. Sequencing of genomic PCR fragments and Southern blotting (Figure S1B, E) suggested that *NtPCLS5-1* and *NtPCLS5-2* are retained only in *N. tabacum*, but not in other *Nicotiana* species examined, such as *N. benthamiana* and *N. megalosiphon*, whereas *PCLS6* was detected in both *N. tabacum* and *N. megalosiphon* (Figure S1B). In contrast, *PCLS7* is conserved in all 4 *Nicotiana* plants tested, although sequence divergence was observed among the *PCLS7*s. Other *PCLS*s from 2 legume plants, *MtPCLS7* and *LjPCLS8* were identified on their nuclear genomes by PCR (Figures S1A, C, D).

### Phylogenetic analysis of the PCLSs

**Figure 4:**
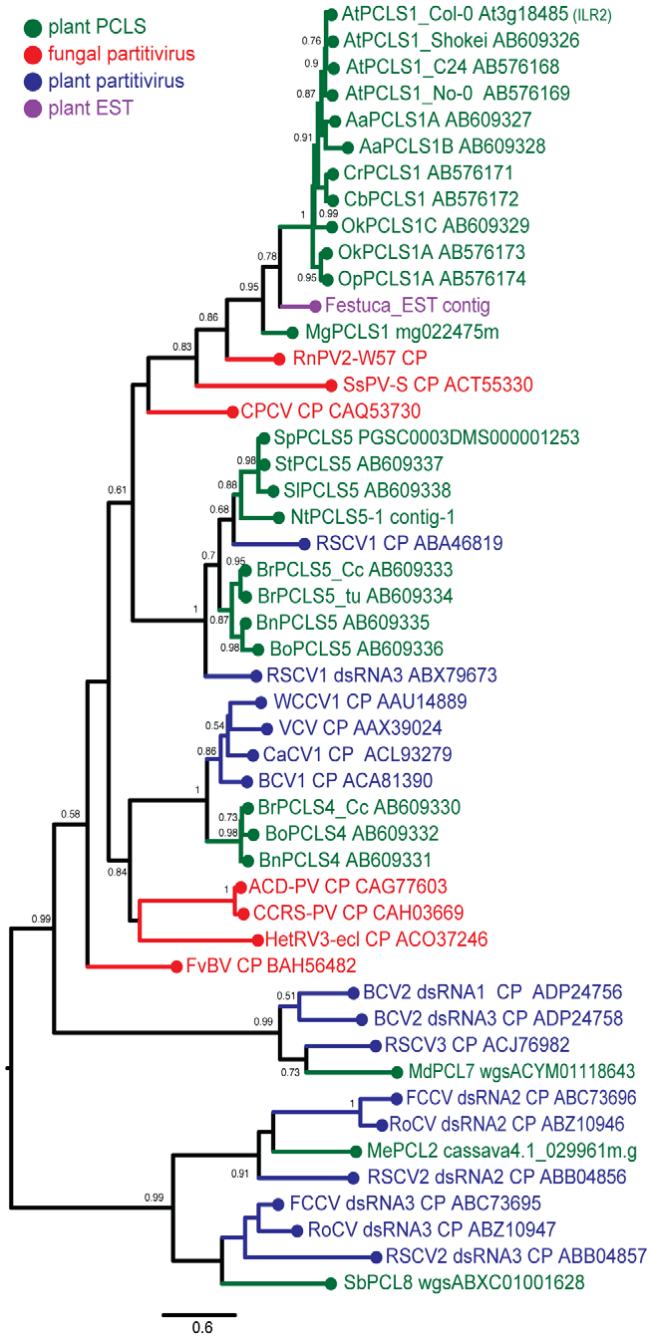
Molecular phylogenetic analysis of partitivirus CPs and plant PCLSs. A phylogenetic tree was generated based on an alignment (see Figure S2) of the entire region of partitivirus CP-related sequences. Analyzed sequences were from 7 fungal partitiviruses (shown in red), 10 plant partitiviruses (in blue), 1 *F. pratensis* EST-derived sequence (shown in purple), 4 accessions of *Ar. thaliana*, and 16 other plant species (in green) (See Tables 1 and S4 for their descriptions). The assembled sequence from the *F. pratensis* ESTs in the database is believed to be of plant-infecting partitivirus origin because the library contains EST entries of RdRp sequences and some had interrupted poly(A) tails typical of a partitiviral mRNA. Viruses analyzed phylogenetically are: Rosellinia necatrix partitivirus 2, RnPV2; Sclerotinia sclerotiorum partitivirus S, SsPV-S; Chondrostereum purpureum cryptic virus, CPCV; Raphanus sativus cryptic virus 1, RSCV1; white clover cryptic virus 1, WCCV1; vicia cryptic virus, VCV; carrot cryptic virus 1, CaCV1; beet cryptic virus 1, BCV1; Amasya cherry disease associated partitivirus, ACD-PV; cherry chlorotic rusty spot-associated partitivirus, CCRS-PV; Heterobasidion RNA virus 3, HetRV3; Flammulina velutipes browning virus, FvBV; beet cryptic virus 2, BCV2; Raphanus sativus cryptic virus 3, RSCV3; Fragaria chiloensis cryptic virus, FCCV; rose cryptic virus 1, RoCV; Raphanus sativus cryptic virus 2, RSCV2. Note that RSCV1 CP gene and RSCV1 dsRNA3, BCV2 dsRNA2 and 3, and RSCV2 dsRNA2 and 3 are assumed to be from two independent viruses although the same virus name was assigned to the segments in the database. Numbers at the branches show aLRT values using an SH-like calculation (only values greater than 0.5 are shown). The scale bar represents the relative genetic distance (number of substitutions per nucleotide).

An expanded BLAST (tblastn) search against the EST sequence libraries (in NCBI) helped detect many related sequences of possible plant partitiviruses that shared moderate levels of sequence similarity. Some representative EST sequences, PCLSs and partitivirus CPs, whose entire sequences are available, were aligned using the MAFFT program. Three relatively well-conserved motifs are located on the N- terminal, central, and C-terminal regions of partitivirus CPs and PCLSs, and are represented by PGPLxxxF [31], F/WxGSxxL and GpfW domains (Figure S2). As expected from sequence similarities, phylogenetic analysis of partitivirus CPs and PCLSs identified in plant genomes clearly show that members of each PCLSs subgroup (PCLS1, 2, 4, 5, 7, 8) clusters together with the CP of the respective partitivirus that shows the highest sequence similarities (Figure 4, Table 1). For example, RnPV2 CP (in red), MgPCLS1, and ILR2 homologs (PCLS1s) from *Arabidopsis*-related genera (in green) constitute one group in the tree. The MgPCLS1 clade includes an assembled sequence in the EST database from meadow fescue (*Festuca pratensis*) (in purple) believed to be from a plant partitivirus. Another group includes PCLS5s from the families Brassicaceae and Solanaceae (in green), CPs of fungal (in red) and plant partitiviruses (in blue) are grouped together. Within this group, PCLSs from the families Brassicaceae (BrPCLS5, BoPCLS5, and BnPCLS5) and Solanaceae (StPCLS5, SpPCLS5, SlPCLS5, and NtPCLS5-1) comprised 2 subgroups that included CPs encoded by RSCV1 (CP) and RSCV1 dsRNA3 (Figure 4), respectively, which are considered to be from two different partitiviruses. PCLS4s from members of the genus *Brassica* clustered together with CPs of other plant partitiviruses including white clover cryptic virus 1 (WCCV1) [32], CaCV1, beet cryptic virus 1 (BCV1) [33], and vicia cryptic virus (VCV) [34].

The tree topology shown in Figure 4 was similar to that reported by Liu et al. [24]. The current study used more PCLSs detected in various plants but not partial PCLSs such as PCLS3 and NtPCLS5-2, 6 and 7 (Tobacco Contig-2, -3 and -4) analyzed phylogenetically by Liu et al. [24].

### Detection of negative-strand RNA viral sequences in plant nuclear genomes

**Figure 5:**
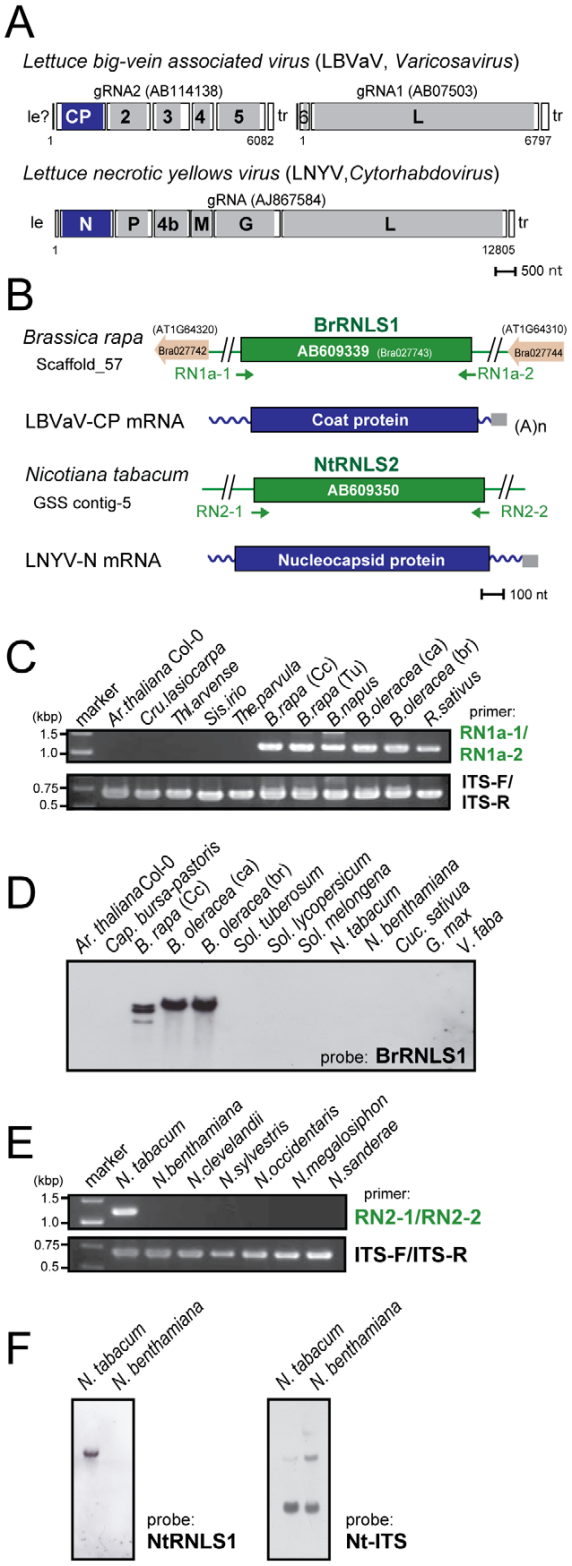
Negative-strand RNA virus-related sequences (RNLSs) from plant nuclear genomes. (A) Genome organization of a varicosavirus, lettuce big-vein associated virus (LBVaV) [67] and a cytorhabdovirus, lettuce necrotic yellows virus (LNYV) [68]. While LBVaV and LNYV have a bipartite and a monopartite genome architecture, respectively, both viruses share similarities in terminal sequence features such as leader sequences (le) and trailer sequence (tr), genome expression strategy and sequences in encoded proteins (e.g., CP vs. N and L vs. L). (B) Schematic representation of *RNLS*s and their flanking regions. *RNLS* found in the genome sequence database of *B. rapa* (*BrRNLS1*) is shown to match that of CP from LBVaV. Another *RNLS* from *N. tabacum* (*NtRNLS2*) showed the greatest similarity to the LNYV-N protein. (C, E) Genomic PCR analysis of *RNLS1* and *RNLS2*. Template genomic DNAs from plant species shown on the top of the gel were used to amplify *RNLS1* (C, top panel), *RNLS2* (E, top panel) or ribosomal RNA ITS regions (C and E, bottom panels). Primer pairs, RN1a-1 and RN1a-2, RN2-1 and RN2-2, and ITS-F and ITS-R were used to amplify *RNLS1*, *RNLS2*, and the ITS regions, respectively. Amplified DNA fragments were electrophoresed in 1.0% agarose gel in TAE. (D, F) Southern blot analyses of plant species in different families. The same DNA preparations as for Figure 1 were used for detection of *RNLS1* (D) and *RNLS2* (F) in which DIG-labeled DNA fragments spanning *BrRNLS1*, *NtRNLS2*, and *N. tabacum* ITS served as probes, respectively. See Figure 3C for hybridization with a *B. rapa* ITS DNA probe.

Because negative-strand RNA viral sequences are found in animal chromosomes, we searched for negative-strand RNA viral sequences (Table S1) in plant genomes as described in the Materials and Methods. This search identified sequences related to the N protein in members of the genus *Cytorhabdovirus* (Lettuce necrotic yellows virus, LNYV, Lettuce yellow mottle virus, LYMoV, and northern cereal mosaic virus, NCMV) and a CP of the genus *Varicosavirus* (Lettuce big-vein associated virus, LBVaV) in the genomes of a variety of plants such as *Populus trichocarpa*, *N. tabacum*, and *B. rapa* (Figures 5, S3, Table 2). While varicosaviruses have bipartite genomes replicated in the cytoplasm of infected plant cells, they are phylogenetically closely related to cytorhabdoviruses with monopartite genomes [35], [36]. Varicosavirus CP is phylogenetically and functionally equivalent to rhabdovirus N. Thus, these plant nuclear sequences were designated as rhabdovirus N-like sequences (RNLSs) and classified into 4 subgroups (*RNLS1* to *RNLS4*) based on the sequences of presently existing viruses with the highest levels of sequence similarities (Table 2). Their potentially encoding proteins were designated as RNLSs as in the case for PCLSs.

**Table 2:** Rhabdovirus nucleocapsid protein (N)-like sequences (*RNLSs*) identified in plant genome sequence databases.

*RNLS*	Plant	Sequence ID	Database	Best-matched virus (abbreviation)	e-value	Mol. analysis^a^
*BrRNLS1-1*	*Brassica rapa*	Bra027743	BRAD	lettuce big-vein associated virus (LBVaV)	9e-08	GP, GS, SQ, PA
*AqcRNLS1*	*Aquilegia coerulea*	AcoGoldSmith_v1.007196m	Phytozome	lettuce big-vein associated virus (LBVaV)	2e-20	(GP, SQ) PA
*MdRNLS1-1*	*Malus x domestica*	unassigned (wgs ACYM01021736, 2134-3297)	NCBI-wgs	lettuce big-vein associated virus (LBVaV)	8e-31	GP, SQ, PA
*MdRNLS1-2*	*Malus x domestica*	unassigned (wgs ACYM01114737, 2849–3310)	NCBI-wgs	lettuce big-vein associated virus (LBVaV)	7e-16	GP, SQ
*LjRNLS1-1*	*Lotus japonicus*	unassigned (gss BABK01031243+cDNA AK339012)	NCBI-gss,-nt	lettuce big-vein associated virus (LBVaV)	6e-12, 7e-13	GP, SQ, PA
*LjRNLS1-2*	*Lotus japonicus*	unassigned (chromosome 3 clone LjT47I22, 60953-62007)	NCBI-htgs	lettuce big-vein associated virus (LBVaV)	2e-16	GP, SQ, PA
*CsRNLS1*	*Cucumis sativus*	unassigned (wgs ACHR01010215, 16588–18054)	NCBI-wgs	lettuce big-vein associated virus (LBVaV)	1e-05	GP, SQ, PA
*TcRNLS1*	*Theobroma cacao*	unassigned (wgs CACC01021584, 28267-27932)	NCBI-wgs	lettuce big-vein associated virus (LBVaV)	1e-03	-
*MgRNLS2*	*Mimulus guttatus*	mgf014425m	Phytozome	lettuce necrotic yellows virus (LNYV)	8e-07	-
*NtRNLS2*	*Nicotiana tabacum*	GSS (Contig-5, Figure S3)	NCBI-gss	lettuce necrotic yellows virus (LNYV)	8e-35	GP, GS, SQ, PA
*NtRNLS3*	*Nicotiana tabacum*	GSS (Contig-6, Figure S3)	NCBI-gss	northern cereal mosaic virus (NCMV)	8e-08	GP, SQ
*PtRNLS4*	*Populus trichocarpa*	POPTR_0008s16330	Phytozome	lettuce yellow mottle virus (LYMoV)	1e-41	PA

a Molecular analysis carried out in this study: GP, genomic PCR; GS, genomic Southern blot; SQ, sequencing; PA, phylogenetic analysis; -, not performed.

To confirm the presence of the *RNLS*s in plant chromosomes, we conducted genomic PCR and Southern blot analyses. Interestingly genomic PCR with primers specific for an *RNLS1* from *B. rapa* (*BrRNLS1*) detected *RNLS1*s in *R. sativus* and all tested plants within the *Brassica* genus, but not in members in other genera (Figure 5C), in a pattern similar to that of *PCLS5s* from the family Brassicaceae (Figure 3B). Consistent with these results, Southern blotting detected hybridization signals in 3 *Brassica* plants (Figure 5D) with a probe specific for *BrRNLS1*.

The *NtRNLS2* sequence was detected in *N. tabacum*, while no fragments were generated from other *Nicotiana* species using genomic PCR (Figure 5E). Southern blotting results supported this detection profile (Figure 5F); *N. tabacum*, but not *N. benthamiana*, was shown to carry an NtRNLS2-related sequence (Figure 5F, left panel).

All other *RNLS*s discovered through the similarity search of genome sequence databanks (Table 2), except for *PtRNLS4* from *Pop. trichocarpa* and *TcRNLS1* from *Theobroma cacao*, were shown to be retained on respective plant genomes by genomic PCR and subsequent sequencing (Figure S3). *RNLS1*s molecularly analyzed included those from *Aquilegia flabellata* (a close relative of *Aq. coerulea*) (*AfRNLS1*), *Lotus japonicus* (*LjRNLS1*), *Malus x domestica* (*MdRNLS1*) and *Cucumis sativus* (*CsRNLS1*) (Figure S3B–H). The *AqfRNLS1* sequence defined in this article showed approximately 98% nt sequence identity to *AcRNLS1* whose sequence is available in the database (Phytozome). *LjRNLS1-1* from *L. japonicus* line B129 and *CsRNLS1* from 3 cucumber varieties (Hokushin, Suyo, and ‘Borszcagowski’ line B10) were identical to the reported *RNLS1* sequences for line MG-20 (Kazusa DNA Research Institute) and ‘Chinese long’ line 9930 [37], respectively. Approximately 97% nucleotide sequence identity was found between *MdRNLS1*s of cultivars ‘Sun-Fuji’ and ‘Golden Delicious.’ ‘Golden Delicious’ is currently used in the apple genome sequence project [38] (http://www.rosaceae.org/projects/apple_genome). These examined *RNLS* sequences are listed in Table S5.

### Phylogenetic analysis of negative-strand RNA virus sequence in plant nuclear genomes

**Figure 6:**
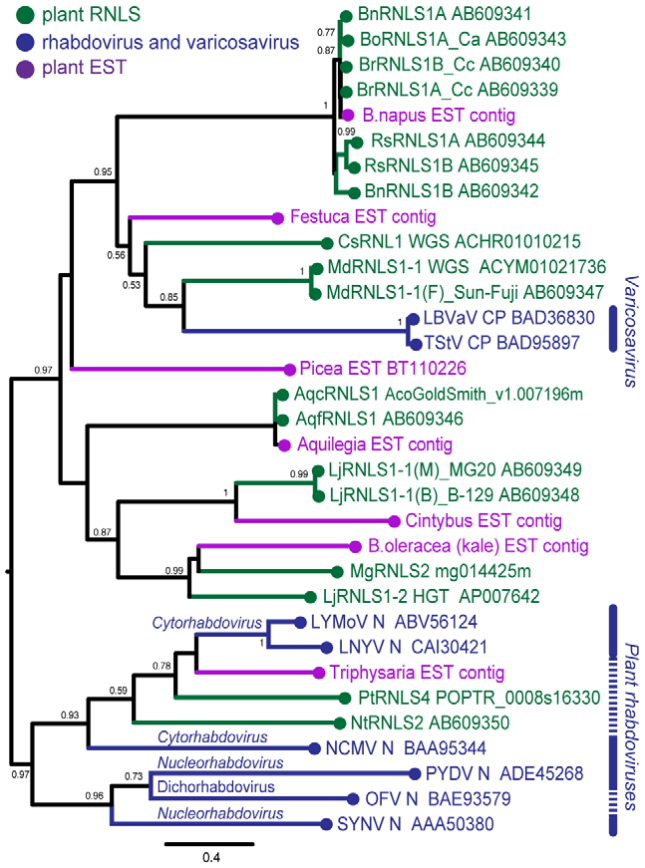
Phylogenetic analyses of the nucleocapsid protein sequences of rhabdoviruses and RNLSs. Phylogenetic relation of nucleocapsid proteins of negative strand RNA viruses and plant RNLSs. A phylogenetic tree was constructed using PhyML 3.0 based on the multiple amino acid sequence alignments of entire regions of rhabdovirus nucleocapsid protein-related sequences shown in Figure S5. Plant RNLSs, N (CP) proteins from negative-strand RNA viruses, and EST-derived sequences are shown in green, blue and purple, respectively. Viruses analyzed phylogenetically are: tobacco stunt virus, TStV; lettuce big-vein associated virus, LBVaV; lettuce yellow mottle virus, LYMoV; lettuce necrotic yellows virus, LNYV; northern cereal mosaic virus, NCMV; potato yellow dwarf virus, PYDV; orchid fleck virus, OFV; sonchus yellow net virus, SYNV. Numbers at the branches show aLRT values using an SH-like calculation (only values greater than 0.5 are shown).

Several sequences found through searching plant EST databases (Table S6, Figure S4) were included in our phylogenetic analysis. Deduced amino acid sequences of plant RNLSs, the N (CP) proteins of negative-strand RNA viruses, and related EST entries were aligned using the MAFFT program (Figure S5). Pair-wise similarities between selected RNLSs and viral N (CP) sequences are shown in Table S7. Two amino acid segments, GmH and YaRifdxxxfxxLQtkxC are relatively well-conserved among these sequences. A dendrogram generated on the basis of alignment showed 4 major groups containing plant RNLSs (Figure 6). RNLS1s are separated into two major groups. The first group includes varicosavirus CPs and RNLS1s from apple, cucumber and *Brassica* plants (MdRNLS1, CsRNLS1, BoRNLS1, and BrRNLS1) in addition to a few ESTs. The second group accommodates RNLS1s from *Aquilegia* and *Lotus* (AqfRNLS1, AqcRNLS1, LjRNLS1), together with an RNLS2 from *Mim. guttatus* (MgRNLS2) and EST sequences from *Cichorium intybus* and *B. oleracea*. The placement of MgRNLS2 in this group may be explained by low-level sequence identity to its most closely related extant varicosavirus, LNYV (Table 2). NtRNLS3, PtRNLS4, and Ns of cytorhabdoviruses (LNYV, LYMoV, and NCMV) form the third group (Figure 6). A dichorhabdovirus (orchid fleck virus, OFV) and nucleorhabdoviruses (PYDV and SYNV), replicating in the nuclei of host plants, are placed into an independent clade.

Whether most of the analyzed ESTs originated from viruses or plant chromosomes is unknown. However, an EST from *F. pratensis* is presumed to originate from a plant virus in our preliminary experiment not only because the N (CP)- but also the L (RdRp)-derived ESTs were detected in the same EST library of *F. pratensis*. This suggests a presently existing virus more closely related to RNLSs of the genus *Brassica* than LBVaV, because both N- and L-related sequences are rarely found in a single plant genome (Table 2).

### Database search for and molecular detection of plus-strand RNA viral sequences in plant genomes

**Figure 7:**
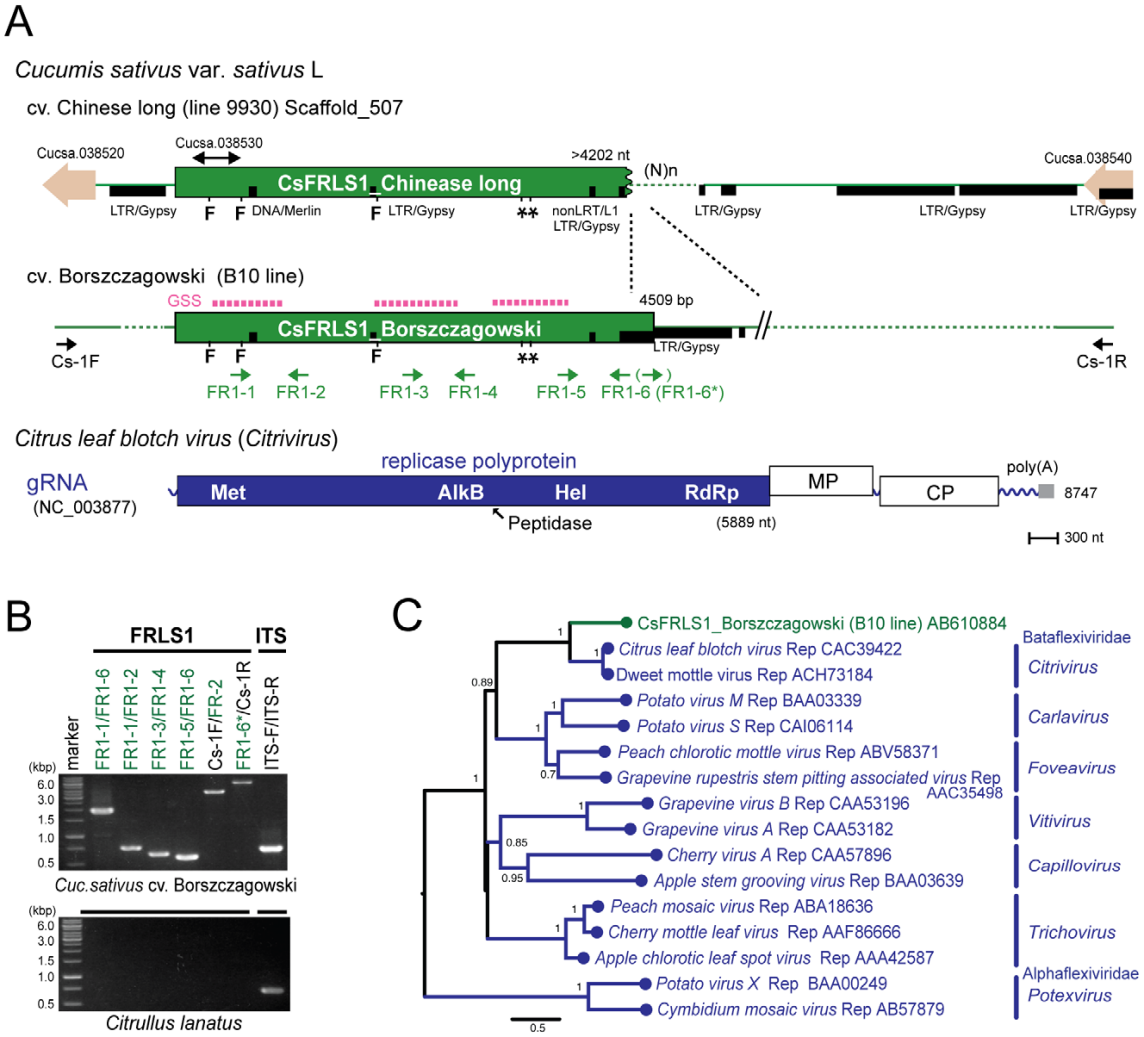
Plant genome sequence related to positive-strand RNA virus. (A) Chromosomal position of the flexivirus replicase-like sequence (*FRLS*) found in the cucumber ‘Chinese long’ inbred line 9930 and the genome structure of a positive-sense RNA virus, citrus leaf blotch virus (CLBV) [39]. A sequence related to the 5′ terminal half of the CLBV genome (*CsFRLS1*) is detected in scaffold 507. Genes for small potential ORFs (Cucsa 038520 and 038540) reside near *CsFRLS1* as well as a retrotransposon-like sequence (shown by thick black lines). Three short sequences identical to *CsFRLS1* are found in the GSS database (NCBI) from a different cucumber line, ‘Borszczagowski’ B10 (http://csgenome.sggw.pl/) (shown by dashed bars above *CsFRLS1* in red). Functional domains of the CLBV replicase polyprotein are indicated in ocher: Met, methyltransferase; AlkB, Fe(II)/2OG-dependent dioxygenase superfamily domain; peptidase; Hel, RNA helicase; RdRp, RNA-dependent RNA polymerase. (B) Detection of *CsFRLS1* from cucumber line by genomic PCR. See Materials and Methods for DNA isolation and PCR reaction. Template genomic DNA was prepared from the cucumber cultivar ‘Borszczagowski’ line B10 (top panel) and *Citrullus lanatus* (watermelon) (bottom panel). Primers (FR1-1 to FR1-6, FR1-6*, CS-1F, and CS-1R) used are shown on the top of the panel. The positions of the primers are shown by arrows below *CsFRLS1* in A, except primer pairs used for amplification of the ITS region (ITS-F and ITS-R) (Table S3). (C) Phylogenetic analysis of CsFRLS1. CsFRLS1 and corresponding amino acid sequences from plant flexiviruses including members of the genera *Citrivirus*, *Carlavirus*, *Foveavirus*, *Vitivirus*, *Capillovirus*, *Trichovirus* and *Potexvirus*, were aligned using MAFFT program (Figure S6). The alignment was then utilized to generate a phylogram. Numbers at the branches show aLRT values using an SH-like calculation (only values greater than 0.5 are shown).

Extensive searches of genome sequence databases for plant plus-strand RNA viral sequences were conducted using genome sequences of various plus-strand RNA viruses representing the major virus genera and families *Potyviridae*, *Luteoviridae*, *Tombusviridae*, and *Bromoviridae* (Table S1). Compared to searches for double- or negative-strand RNA viral sequences, the search for plus-strand RNA virus sequences yielded a much smaller number of hits. The *Medicago truncatula* database (HTGS) contains sequences of 320 and 475 nts with over 98% sequence identity to the capsid and movement protein genes of cucumber mosaic virus, a member of the family *Bromoviridae*. However, this sequence was not amplified in *Med. truncatula* line A17 used in the genome sequence project by genomic PCR with different sets of internal and external primers. A sequence similar to replication-related genes of citrus leaf blotch virus (CLBV) [39] belonging to the family *Betaflexiviridae*, is identified in the complete genome databases for the cucumber ‘Chinese long’ line 9930 [37] and termed *Cucumis sativus* flexivirus replicase-like sequence 1, CsFRLS1 (Figure 7A). The GSS database of cucumber ‘Borszczagowski’ line B10 also contains CsFRLS1 (http://csgenome.sggw.pl/), but its available sequence is fragmented (Figure 7A, dashed purple bar) and shorter than that in the complete genome sequence data base.

Two independent cucumber genome databases for 2 different lines strongly suggest the presence of *CsFRLS1* in the cucumber chromosome. We confirmed this by genomic PCR using different sets of primers corresponding to methyltransferase (Met) and RNA helicase (Hel) domains, the inter-domain region (FR1-3 and FR1-4) and the entire *CsFRLS1* region (Figure 7B). DNA fragments of expected sizes were amplified on genomic DNA from the ‘Borszczagowski’ line B10, but not from watermelon, *Citrullus lanatus* (Figure 7B). Furthermore, genomic PCR fragments covering FRLS1 and its flanking putative open reading frames (ORFs) were amplified, strongly suggesting that *FRLS1* resides on the nuclear genome as shown in Figure 7A and B. The phylogenetic tree containing CsFRLS1 potentially encoded by *CsFRLS1* and its counterparts from related viruses shows that CsFRLS1 is closely related to the genus *Citrivirus* within the family *Betaflexiviridae* (Figure 7C). The distance between CsFRLS1 and citriviruses are similar to intra-genus distances in the genera *Carla*-, *Fovea*-, *Viti*- and *Potexviruses*.

## Discussion

The finding that the CP of a novel partitivirus, RnPV2 from a fungal phytopathogen matched a plant gene product, ILR2 from *Ar. thaliana* initiated a comprehensive search of the plant genomic sequence data available as of December 10, 2010 for non-retroviral RNA virus sequences (NRVSs) in plant genomes. While this study showed a variety of sequences related to the *N* (*CP*) genes of negative-stranded RNA viruses (cytorhabdoviruses and varicosaviruses) in members in the plant families including Solanaceae, Leguminosae, Brassicaceae and Phrymaceae, only one plus-sense RNA virus-related sequence (betaflexivirus replication-related gene) was found to be present in the cucumber genome. Furthermore, this survey detected sequences related to CP from dsRNA viruses (partitiviruses) (PCLSs) in various plants in addition to PCLSs reported by Liu et al. [24]. These authors performed a thorough search of eukaryotic genomic sequences available as of September 2009 for NRVSs and showed multiple dsRNA virus-related sequences not only in plants but also animals. Importantly, many of the NRVSs revealed by BLAST searches in this study were subsequently identified in plant genomes by Southern blotting, genomic PCR and sequence analyses (Figures 1–3, 5, 7, S1, S3). These findings provide interesting insights into plant nuclear genome evolution, plant phylogeny and virus/host interactions.

**Figure 8:**
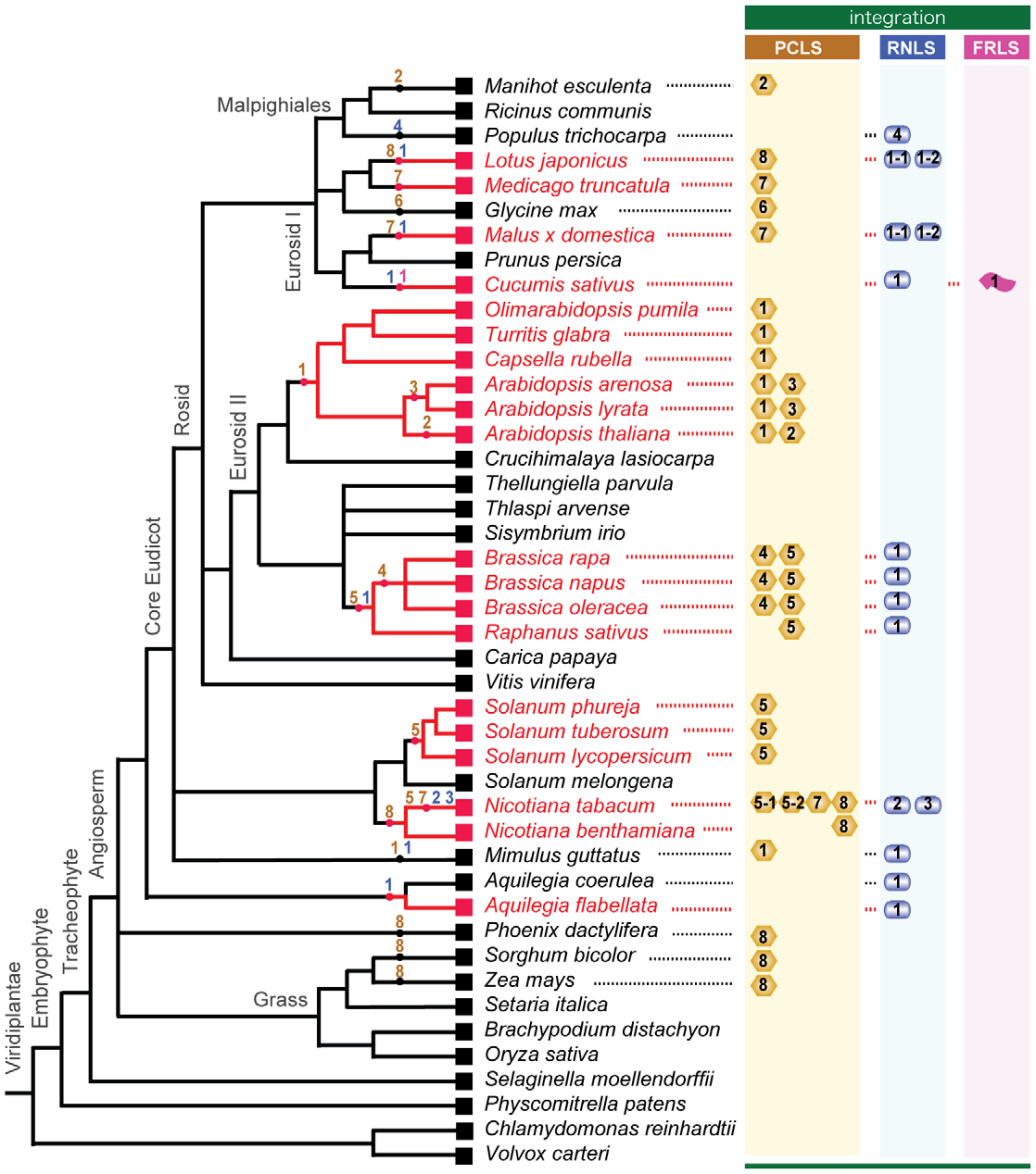
Horizontal gene transfer of genome sequences of non-retroviral RNA viruses into plant genomes. The cladogram was created based on previous reports by The Angiosperm Phylogeny Group (31) [69], Oyama et al. [43], Udvard et al. [70] and Phytozome (http://www.phytozome.net/). Plants whose integrated non-retroviral RNA virus sequences (NRVSs) were analyzed molecularly in this study are shown in red. Integrations of non-retroviral RNA virus sequences, *PCLSs*, *RNLSs*, and *FRLS* are shown next to the plant species retaining them. Presumed integration times of NRSVs are indicated by dots on the nodes. Numbers within the genome-integrated NRVSs refer to subgroups (possible different virus origins) of *PCLS* (yellow column), *RNLS* (blue column) and *FRLS* (pink column) (Tables 1, 2, S4, S5). Numbers are placed within or beneath the symbolized morphologies of viruses that are thought to be the source of integrations (spherical for partitivirus, *PCLS*; bacilliformed for rhabdovirus, *RNLS*; flexuous for betaflexivirus, *FRLS*).

Horizontal gene transfer, HGT, can occur “from virus to plant” or “from plant to virus.” A retention profile of *PCLS1* among plants strongly suggests that HGT may have involved the former direction. The family Brassicaceae of the order Brassicales includes the genus *Arabidopsis*, which is believed to have diverged after the split of the families Phrymaceae and Solanaceae, accommodates the genera *Mimulus* and *Solanum* and belong to different orders, Lamiales and Solanales, respectively (Figure 8). No *PCLS1* homologs are found in *Vitis vinifera* or *Carica papaya*, and that this gene resides on non-orthologous chromosomal positions of *Mim. guttatus* (data not shown) and *Arabidopsis*-related species (Figure 1A). This strongly suggests that independent HGT events from virus to the *Arabidopsis* and *Mim. guttatus* lineages may have occurred (Figure 8). This observation is also true for other *PCLS*s. The families Solanaceae and Brassicaceae contain *PCLS5s*, while their counterparts are not found in other plants whose complete genome sequences are available (Figure 8). The observation that a relatively widely conserved gene *PUX_4* is disrupted in *Sol. phureja* by *SpPCLS5* (Figure 3A) provides additional evidence for its insertion into the *PUX_4* locus. The HGT direction “from virus to plant” was further confirmed by phylogenetic analysis showing that plant PCLSs and partitivirus CPs are placed in a mixed way (Figure 4). Viral sequences are basal in each of the three major clades, supporting the direction of transfers from virus to plant.

The divergence time of plant lineages is estimated through a classical approach using fossils and mutations rates of some particular genes. Alternatively, if we assume that cellular genes evolve at a constant rate, their divergence time can be calculated from the genome-wide, spontaneous mutation rate determined on a generation basis in the laboratory [40]. Together with the patterns of occurrence of the non-retroviral integrated RNA virus sequences, these values allow us to estimate time of some, if not all, HGTs identified in this study. For example, the integration of *PCLS1* (*ILR2*) may have post-dated the split of the lineages containing the genera *Arabidopsis* and *Brassica* (16.0–24.1 million years ago) and pre-dated the speciation of *Arabidopsis* spp., or more accurately the divergence of *Arabidopsis* and its closely related genera (Figure 8) (10–14 million years ago) [40], [41], [42]. The phylogenetic relation among PCLS1s from *Arabidopsis* and its close relatives within the tribe Camelina (*Capsella*, *Olimarabidopsis*, and *Turritis*) agrees with the phylogeny of the family Brassicaceae deduced from systematic analyses [43]. Moreover, assuming that the *Ar. thaliana* and *Ar. arenosa* separated 10 million years ago, the mutation rates calculated for *PCLS1*s between the 2 plants are estimated to be 6.8×10^−9^ base substitutions per site per year, a value close to the genome-wide base substitution rate, 7×10^−9^, reported for *Ar. thaliana* by Ossowski et al. [40]. These observations suggest that endogenized *PCLS1*s accumulated mutations in a manner similar to those of other nuclear sequences during the course of evolution after a single HGT event in an ancestral *Arabidopsis* plant.

The genome of *B. rapa* in the family Brassicaceae retained 2 *PCLS*s (*BrPCLS4* and *BrPCLS5*) with low-level similarities to RnPV2 CP on chromosomal positions different from each other and from that of the *PCLS1* (*ILR2*) homologs of *Arabidopsis*-related genera. No *PCLS1* homolog was identified on the orthologous positions of the *B. rapa* genome, and no *BrPCLS4* or *BrPCLS5* homologs were found on the corresponding locus of the *Ar. thaliana* or *Ar. lyrata* genome. Therefore, *BrPCLS4* and *5* may have been introduced into the *B. rapa* genome separately from each other and from *PCLS1* (*ILR2*) after the divergence of the *Brassica* and *Arabidopsis* lineages (Figure 8). Similarly, the detection profile of *AtPCLS2* and *AlPCLS3* (Figure 2) shows that they may have been introduced into *Ar. thaliana* and *Ar. lyrata* chromosomes independently after the separation of 2 plant species (3.0–5.8 million years ago) (Figure 8); these are more recent HGT events than the *PCLS1* integration into the *Arabidopsis* lineage. *PCLS* integrations into the Solanaceae lineage were slightly complex. Relatively high or moderate levels of aa sequence identities (47–68%) are shared within the PCLS5s from the family Solanaceae. However, a lack of information regarding genome sequences flanking the *PCLS5*s caused difficulty in determining whether a single event or multiple HGT events may have occurred within the lineage (Figure 8).

Gene sequences related to rhabdovirus or varicosavirus *N* (*CP*) genes (*RNLS*s) are detected in many genera including *Brassica*, *Raphanus*, *Mimulus*, *Nicotiana*, *Lotus*, *Malus*, *Cucumis*, *Populus*, *Theobroma*, and *Aquilegia* (Figures 5, 8, S3). Using similar rationale for the HGT of *PCLS*s, multiple integrations of *RNLS*s into plant chromosomes are thought to have occurred (Figure 8). RNLSs are distributed in an irregular manner in the plant lineage, while rhabdovirus N proteins show similar tree topology to that exhibited by corresponding RdRps. This is consistent with the hypothesis that HGT occurred “from virus to plants.” *RNLS2* was detected in a very narrow range of plants, i.e., detectable only in *N. tabacum* but not other *Nicotiana* species (Figure 5). *RNLS1* was detected in all tested *Brassica* species, *R. sativus* and *Aq. coerulea*, while it was not detected in the genomes of *Ar. thaliana* [25] or *Ar. lyrata* (Phytozome), which are much closely related to *Brassica* than *Aq. coerulea* to *Brassica*. If these sequences were of plant origin, homologous sequences are expected to be retained at least within some members of the families Brassicaceae and Solanaceae. However, Southern blotting and genomic PCR analyses with *NtRNLS2*- and *BrRNLS1*-specific probes and primers failed to detect their related sequences in plants other than *N. tabacum*, and *Brassica* species and *R. sativus*, respectively (Figure 5C–F). A search using *NtRNLS2* and *BrRNL1* against the genome sequences of *Ar. thaliana* and *Ar. lyrata* did not yield any hits. This indicates that multiple HGTs of *RNLS*s occurred from “virus to plant.” While the *BrRNLS1* integration may have postdated the split of the *Arabidopsis* and *Brassica* lineages (43.2–18.5 million years ago), *NtRNLS2 and NtRNLS3* integration may have occurred after the divergence of *N. tabacum* (allotetraploid) and its maternal parent *N. sylvestris* (diploid) (0.2 million years ago) [44]. This hypothesis must be verified by sequence analysis of the corresponding regions of *N. tabacum* and other *Nicotiana* species.

The detection pattern of *PCLS*s within the family Brassicaceae provided an interesting insight into the phylogenetic relationship of some genera in the family. The family Brassicaceae is one of the largest families comprising over 300 genera and approximately 3,300 species that include an important plant biology model plant, *Ar. thaliana*, and agriculturally important *Brassica* species. Their phylogenetic relationships have been extensively studied and are occasionally controversial, because they rely on data sets and methods exploited for analyses. For example, placement of the genus *Crucihimalaya* is interesting to note in relation to this study. The genus is placed into a clade containing the genus *Boechera*, and is assumed to have separated from an ancestor common to the genus *Capsella* after the divergence of the *Arabidopsis* lineage based on phylogenetic analyses with a single nuclear gene (*chalcone synthase gene*) [45] or multiple data sets containing plastid and nuclear genes [45], [46], [47], [48]. However, utilization of different data sets shows different tree topologies, suggesting that the *Crucihimalaya* genus may have diverged before the split between *Arabidopsis* and *Capsella* [45], [49]. *PCLS1*s (*ILR2* homologs) were detected in relatives of *Arabidopsis* but not in *Cru. lasiocarpa* (Figure 1B, D), strongly supporting the phylogenetic relation proposed by Lysak et al. [49]. The absence of the *PCLS1* in a homologous position of the *Cru. lasiocarpa* chromosome was confirmed by sequencing of genomic PCR fragments generated with a specific primer set (Figure 1D). Therefore, these results clearly indicate that *PCLS*s have the potential to supplement phylogenetic estimates by serving as molecular markers. Furthermore, a similar insight into phylogenetic relations among *Nicotiana* species may be gained from data regarding 4 *PCLS*s identified in *N. tabacum* as more data in the genome and *PCLS* sequences of the genus *Nicotiana* become available.

Many examples of HGT from minus-sense RNA and dsRNA viruses, particularly from partitiviruses, have been found in plant nuclear genomes. Endogenization of NRVSs required 3 events to occur: (1) replication of the ancestral viral genome in the germ lines of host plants, (2) reverse transcription of genomic RNA, and (3) its subsequent integration into plant chromosomes. Many plant viruses are reported to be transmitted through pollens and seeds [50], while their transmission rates depended on virus/host combinations. Seed-transmitted viruses include positive-strand and negative-strand RNA viruses and partitiviruses with dsRNA genomes. The family *Partitiviridae* accommodates members that infect plants or fungi, and some plant and fungal partitiviruses are phylogenetically closely related ([21]; Figure 4). PCLS1 is most closely related to a novel fungal partitivirus, RnPV2, but the other PCLSs show the closest resemblance to plant partitiviruses (Table 1, Figure 4). Therefore, *PCLS1* integration occurred when an ancestor of RnPV2 acquired the ability to infect an ancestral plant during endosymbiotic [51] or parasitic interactions between its host fungus and the plant, a host of the fungus, and to invade the plant germ cells. In support of this hypothesis, an assembled EST sequence is present in *F. pratensis* that is more closely related to *PCLS1* than the RnPV2 CP gene and considered to have originated in a plant partitivirus (Figure 4). Such a virus may have been a direct source of plant *PCLS1*. Alternatively some fungal partitiviruses may be intrinsically able to infect plant cells. The expected capability of plant partitiviruses to replicate in host germ cells may be associated with their high rates (∼100%) of seed transmission via ovule and/or pollen [21], an uncommon phenomenon for plant viruses. Although germ lines are hypothesized to have the ability to eliminate virus infection, partitivirus may be able to overcome such a host mechanism. It is also likely that ancestral negative-strand RNA viruses may have invaded germ cells of host plants.

For the second required event, integration of NRVSs likely involved reverse transcription that may have been mediated by reverse transcriptase encoded by retrotransposons or pararetroviruses. However, the mechanism by which the viral RNA sequences were converted to DNA and introduced into plant genomes remains unknown. Interestingly *LjPCLS8* harbors an unrelated sequence of 1.3-kb sequence in its central region (Figure S1A, D), suggesting a recombination event of during reverse transcription or a 2-step integration of 2 distinct molecules, PCLS8 and a sequence of an unknown origin. For the third event, as suggested by Liu et al. [24], transposon-mediated integration [52] and/or double-strand-break repair (non-homologous recombination) [53] may be involved. Flanking regions of some plant genome-integrated NRVSs (e.g., *RNLS1s* and *CsFRLS1*, see Figures 7, S3) carried transposable elements or multiple repeat sequences, supporting the first type of integration. Vertebrate cultured cells are useful for experimentally monitoring *de novo* integrations of negative-strand RNA viral sequences [11]; however, the agents that facilitate the reverse transcription and integration steps remain unknown.

In contrast to the nuclear integrations of partitivirus *CP* sequences and negative-strand RNA virus *N* sequences, plus-strand RNA virus endogenizations were observed much less frequently. A level of viral transcripts in germ cells may be one of factors governing the frequency of NRVSs. This is supported by the observation (data not shown) that, whereas we searched for integrated partitiviral RdRp sequences or other non-*N* sequences of rhabdoviruses, we could seldom detect them. Partitivirus CP and rhabdovirus N coding transcripts are highly likely to be produced in cells infected by the respective viruses more than other viral transcripts. Plus-strand RNA viruses, are believed to accumulate in infected plant cells much more than plant partitiviruses. However, plus-strand RNA viruses may generally be more able to be detected by a surveillance system of host germ cells and/or less competent to escape from their defense system. A smaller number of *FRLS* integrations observed in this study (Figure 7) may be associated with a lower ability of ancestral plus-strand RNA viruses to invade host germ cells, as predicted from the low seed transmissibility of CLBV [54]. Alternatively, plus-strand RNA virus sequences are disfavored by reverse transcriptase and agents that facilitate integration of their complementary DNA in the second and third events, respectively, although this possibility may be low.

## Materials and Methods

### Fungal strains and virus characterization

A virus-infected fungal strain of *R. necatrix*, W57, was isolated in the Iwate Prefecture, Japan. Molecular characterization of genomic dsRNAs were performed according to the methods described by Chiba et al. [55], unless otherwise mentioned.

### Plant materials and gene characterization

Seeds for members of the Brassicaceae family, *L. japonicus*, *Med. truncatula* and *Cuc. sativus* cv. Borszczagowski B10 line were provided by the Arabidopsis Biological Resource Center of The Ohio State University, the Frontier Science Research Center, University of Miyazaki, and Drs. Kazuhiro Toyoda, Douglas Cook, and Grzegorz Bartoszewski, respectively. Seeds for members of the genus *Nicotiana* were originally obtained from Nihon Tabako, Inc (Tokyo, Japan) and maintained at Okayama University. Dr. Takashi Enomoto of Okayama University provided the remaining plants. Plant genomic DNA was isolated from seeds or fresh leaf materials and used in genomic PCR and Southern blot analyses as described by Miura et al. [56]. Sequences of *ILR2* homologs (*PCLS1s*) from members of the family Brassicaceae, except for *Ar. thaliana* accessions Col-0 and WS, and *Ar. lyrata*, were obtained by sequencing genomic PCR fragments. Genomic PCR fragments or clones were used to determine the sequences of other selected *PCLSs*, *RNLSs* and *FRLSs*. Digoxigenin (DIG)-labeled DNA, prepared as described by Chiba et al. [55], was used as probes in Southern blotting analyses as described by Faruk et al. [57]. Table S3 includes sequences of primers used in this study.

### Database search and phylogenetic analysis

BLAST (tblastn) searches [58] were conducted against genome sequence databases available from the NCBI (nucleotide collection, nr/nt; genome survey sequences, GSS; high-throughput genomic sequence, HTGS; whole-genome shotgun reads, WGS; non-human, non-mouse ESTs, est others) (http://www.ncbi.nlm.nih.gov/), Phytozome v6.0 (http://www.phytozome.net/), Brassica database (BRAD) (http://brassicadb.org/brad/), Potato Genome Sequencing Consortium (http://potatogenomics.plantbiology.msu.edu/), and Kazusa DNA Research Institute (http://www.kazusa.or.jp/e/index.html). The databanks covered the complete and partial genome sequences of 20 plant species. Transposable element sequences were identified using the Censor (http://www.girinst.org/censor/index.php) [59]. Obtained non-retroviral integrated sequences were translated to amino acid sequences and aligned with MAFFT version 6 under the default parameters [60] (http://mafft.cbrc.jp/alignment/server). For some non-retroviral integrated sequences with interrupted ORFs, frames were restored by adding Ns as unknown sequences to obtain continuous aa sequences (edited residues are shown as Xs). Alignments were edited by using MEGA version 4.02 software [61]. To obtain appropriate substitution models for the maximum likelihood (ML) analyses, each data set was subjected to the Akaike information criterion (AIC) calculated using ProtTest server [62] (http://darwin.uvigo.es/software/prottest_server.html). According to ProtTest results, WAG+I+G+F, LG+I+G, and LG+I+G+F were selected for PCLSs and partitiviruses, for RNLSs, plant rhabdoviruses and varicosaviruses, and for FRLS and flexiviruses, respectively. Phylogenetic trees were generated using the appropriate substitution model in PhyML 3.0 [63] (http://www.atgc-montpellier.fr/phyml/). In each analysis, four categories of rate variation were used. The starting tree was a BIONJ tree and the type of tree improvement was subtree pruning and regrafting (SPR) [64]. Branch support was calculated using the approximate likelihood ratio test (aLRT) with a Shimodaira–Hasegawa–like (SH-like) procedure [65]. The tree was midpoint-rooted using FigTree version 1.3.1 software (http://tree.bio.ed.ac.uk/software/).

### Data deposition

Two mycoviral genome sequences and a total of 73 non-retroviral integrated RNA virus sequences were analyzed. Sequence data (1 of the 2 genome segments of RnPV2, 21 *PCLS*s, 12 *RNLS*s and 1 *FRLS*) used for phylogenetic analyses in this article have been deposited into the EMBL/GenBank/DDBJ Data Library under the following accession numbers: AB569998 (RnPV2 dsRNA2), AB576168–AB576175, AB609326–AB609329 (*ILR2*-like sequences: *PCLS1s*), AB609330–AB609338 (*PCLS2*–*PCLS8*), AB9339–AB609350 (*RNLSs*), and AB610884 (*CsFRLS1*) (Tables S4 and S5). Other non-retroviral integrated RNA virus elements whose sequences were partially determined and analyzed in this study are available upon request.

## Supporting Information

Figure S1 **Detection of** ***PCLS*** **s from members of the genus** ***Nicotiana*** **, and** ***Med. truncatula*** **and** ***L. japonicus*** **.** (A) Schematic representation of genome organization of partitivirus genome segments and *PCLS*s from *N. tabacum*, *Med. truncatula* and *L. japonicus*. RSCV1 dsRNA2 encodes CP of 505 amino acids that is closely related to PCLS5s, including NtPCLS5-1 and NtPCLS55-2. NtPCLS5-1 and NtPCLS5-2 share 47% sequence identity. NtPCLS6 and NtPCLS7 show the highest levels of similarity to the C-terminal and central portions of CPs encoded by FCCV dsRNA3 and RSCV3 dsRNA2, respectively. These *NtPCLS*s were detected in contigs independently assembled with sequences in the NCBI GSS database [24]. See the Figure 1 legend for explanation of the symbols. (B–D) Genomic PCR analyses of *PCLS 5* to *PCLS8*. PCR was carried out on DNA templates from *Nicotiana* plants (B), three lines of *Med. truncatula* (C), and 2 lines of *L. japonicus* and *G. max* (D), as shown on the top of the panel. Primer sets specific for *NtPCLS5-1* (PC5-1-1 and PC5-1-2), *NtPCLS5-2* (PC5-2-1 and PC5-2-2), *NtPCLS6* (PC6-1 and PC6-2), *NtPCLS7* (PC7a-1 and PC7a-2), *MtPCLS7* (PC7b-1 and PC7b-2), *LjPCLS8* (PC8-1 and PC8-2), and the ITS region (ITS-F and ITS-R) were used for PCR and indicated at the right of the panels. Primer positions are shown in A. (E) Southern blotting of *PCLS5* to *PCLS7*. *Eco*RI-digested genomic DNA was used for detection using DIG-labeled DNA probes specific for *NtPCLS5-1*, *NtPCLS5-2*, *NtPCLS6*, *NtPCLS7* and *NbPCLS7*, and *N. tabacum* ITS. Four plant species, *B. rapa*, *Sol. tuberosum*, *N tabacum*, and *N. benthamiana* were analyzed. *NtPCLS5-2* possesses an internal *Eco*RI recognition site. No cross-hybridization was observed on Southern blots under the conditions used in this study between *NtPCLS7* and *NbPCLS7*, which share 75% nucleotide sequence identity with 6 gaps between the sequences (compare sizes of PCR fragments in lane *N. tabacum* and the other lanes of the fourth panel of Figure S1B).
(TIF)

Figure S2 **Alignment of plant partitivirus CPs and their related plant sequences (PCLSs).** The entire region of RnPV2 CP (aa 1–483) was aligned with homologous sequences from other plant and fungal partitiviruses, translated EST sequences of possible plant partitivirus origin, and plant PCLSs using the program MAFFT version 6. The alignment was used to generate a phylogenetic tree, as shown in Figure 4. For full virus names and information on PCLSs, see the Figure 4 legend, and Tables 1 and S4. Three relatively well conserved sequences, PGPLxxxF, F/WxGSxxL and GpfW domains are marked in red.
(PDF)

Figure S3 **Schematic representation of chromosomal positions of** ***RNLS*** **s and their detection by genomic PCR.** (A) Map positions of a total of 11 *RNLS*s are depicted. Their source plants, such as cucumber, apple, and *N. tabacum* are shown at the left. RNLS1s showed highest levels sequence similarities to the CP of LBVaV, while the other RNLSs are most closely related to the N protein of cytorhabdovirus, either lettuce necrotic yellows virus (LNYV), northern cereal mosaic virus (NCMV) or lettuce yellow mottle virus (LYMoV). Contigs were constructed from GSSs of *N. tabacum* as shown below the chromosomal positions of *NtRNLS2* and *NtRNLS3*. Sequences related to transposable elements or repeat sequences are shown by black thick lines. See the legend to Figure 1 for explanation of the other symbols. (B–H) Molecular detection of *RNLS*s from several plants. Representative *RNLSs* from *Aq. flabellata* (B), *Mal. domestica* (C, D), *L. japonicus* (E, F), *Cuc. sativus* (G), and *N. tabacum* (H) were detected by genomic PCR and sequencing. Primers' positions and sequences are shown in A (arrows) and Table S3. Entire regions of *RNLS*s were amplified in all PCR assays (A to F), while in panels B and E partial forms of *RNLS*s were also amplified. Most sequences of DNA fragments were identical to those available from the respective genome sequence databases.
(TIF)

Figure S4 ***RNLS*** **Contigs constructed from EST libraries of different plants.** Rhabdovirus nucleocapsid (N)-like sequences were detected by searching EST databases for *F. pratensis*, *Aq. formosa*×*Aq. pubescens*, *B. oleracea*, *B. napus*, *Cic. intybus*, *Picea glauca*, and *Triphysaria pusilla*. Multiple ESTs were used to construct contigs where overlapping regions of EST sequences show over 99% sequence identity. These ESTs are either from endogenized viral sequences or infecting viruses.
(TIF)

Figure S5 **Alignment of plant rhabdovirus and varicosavirus N proteins and plant nuclear encoded RNLSs.** The entire nucleocapsid protein (N) sequences (approximately 450 aa) of plant rhabdoviruses and varicosaviruses (approximately 450 aa) and plant rhabdovirus N-like proteins (RNLSs) were aligned using the program MAFFT version 6. The alignment was used to generate a phylogenetic tree, as shown in Figure 6. For non-abbreviated virus names and information on RNLSs, see the Figure 6 legend, and Tables 2 and S5. Two conserved motifs GmH and YaRifdxxxfxxLQtkxC are marked in red.
(PDF)

Figure S6 **Alignment of a plant nuclear encoded FRLS and betaflexivirus replicase proteins.** The partial replicase polyprotein sequences (approximately 1500 aa) from *Cuc. sativus* (cucumber) (CsFRLS1), all *Betaflexiviridae* genera (Citri-, Carla-, Fovea-, Viti-, Capillo-, and Trichoviruses) and a member of the family *Alphaflexivirdae* (potato virus X) were aligned using the program MAFFT version 6. The alignment was used to generate a phylogenetic tree, as shown in Figure 7C. Conserved methyltransferase, RNA helicase (partial), and RdRp motifs are marked in red.
(PDF)

Table S1 **Virus gene sequences used as query sequences in the search for non-retroviral integrated RNA viruses.**
(DOC)

Table S2 **Amino acid sequence identities among selected partitivirus CPs and plant Partitivirus CP-like sequences (PCLSs).**
(DOC)

Table S3 **Oligonucleotide primers used in this study.**
(DOC)

Table S4 **Partitivirus CP-like sequences (PCLSs) analyzed in this study.**
(DOC)

Table S5 **Rhabdovirus N-like sequences (RNLSs) analyzed in this study.**
(DOC)

Table S6 **Rhabdovirus N-like sequences (RNLSs) identified in plant EST collections.**
(DOC)

Table S7 **Amino acid sequence identities among selected rhabdovirus Ns/CPs and plant rhabdovirus N-like sequences (RNLSs).**
(DOC)
