# Supplementary figures and images for: Widespread Endogenization of Genome Sequences of Non-Retroviral RNA Viruses into Plant Genomes
Source: PLoS Pathog. 2011 Jul 14;7(7):e1002146. doi: 10.1371/journal.ppat.1002146 (PMC3136472; doi:10.1371/journal.ppat.1002146)

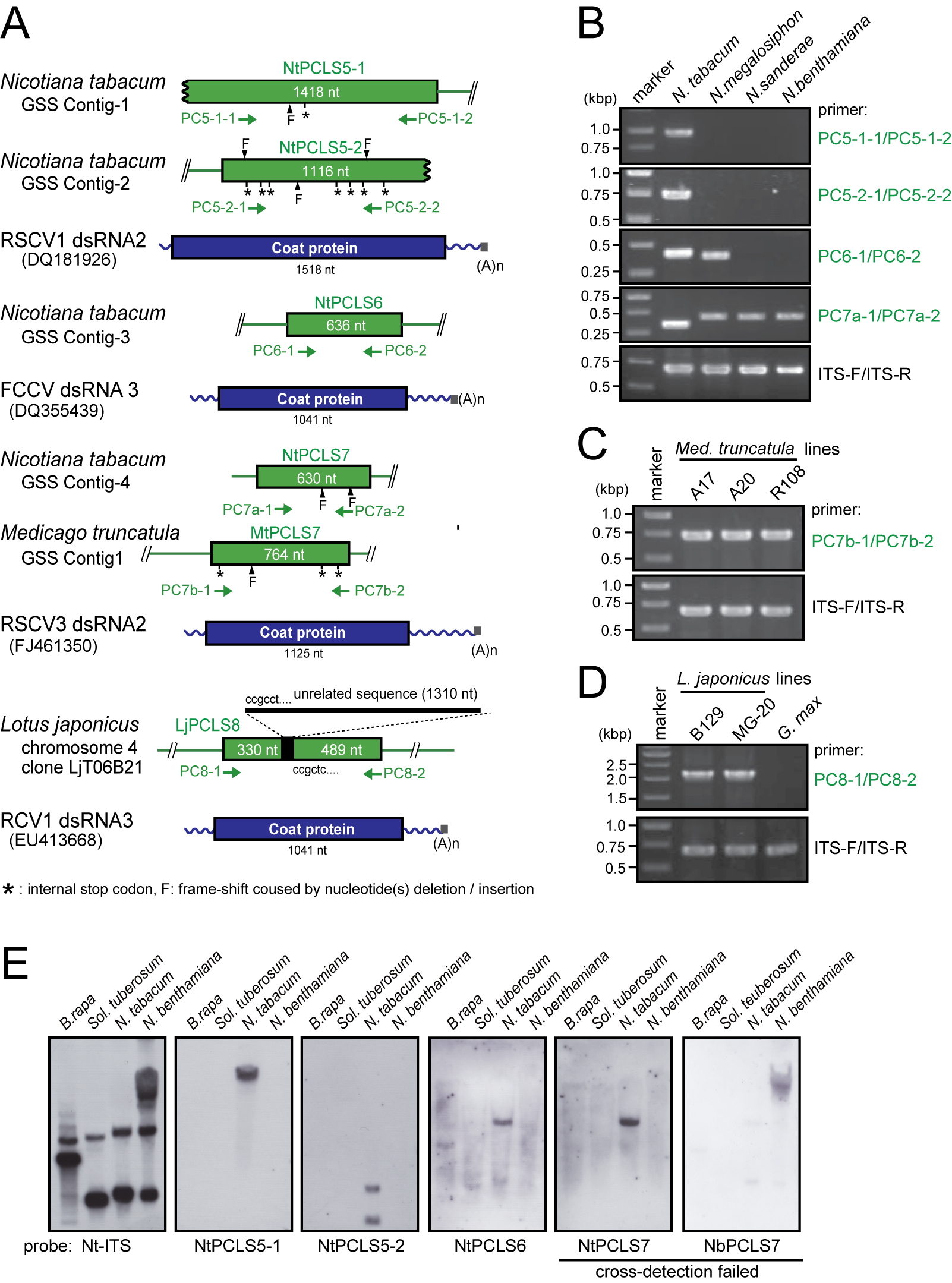

Supplement: Figure S1 — Detection of PCLS s from members of the genus Nicotiana , and Med. truncatula and L. japonicus . (A) Schematic representation of genome organization of partitivirus genome segments and PCLSs from N. tabacum, Med. truncatula and L. japonicus. RSCV1 dsRNA2 encodes CP of 505 amino acids that is closely related to PCLS5s, including NtPCLS5-1 and NtPCLS55-2. NtPCLS5-1 and NtPCLS5-2 share 47% sequence identity. NtPCLS6 and NtPCLS7 show the highest levels of similarity to the C-terminal and central portions of CPs encoded by FCCV dsRNA3 and RSCV3 dsRNA2, respectively. These NtPCLSs were detected in contigs independently assembled with sequences in the NCBI GSS database [24]. See the Figure 1 legend for explanation of the symbols. (B–D) Genomic PCR analyses of PCLS 5 to PCLS8. PCR was carried out on DNA templates from Nicotiana plants (B), three lines of Med. truncatula (C), and 2 lines of L. japonicus and G. max (D), as shown on the top of the panel. Primer sets specific for NtPCLS5-1 (PC5-1-1 and PC5-1-2), NtPCLS5-2 (PC5-2-1 and PC5-2-2), NtPCLS6 (PC6-1 and PC6-2), NtPCLS7 (PC7a-1 and PC7a-2), MtPCLS7 (PC7b-1 and PC7b-2), LjPCLS8 (PC8-1 and PC8-2), and the ITS region (ITS-F and ITS-R) were used for PCR and indicated at the right of the panels. Primer positions are shown in A. (E) Southern blotting of PCLS5 to PCLS7. EcoRI-digested genomic DNA was used for detection using DIG-labeled DNA probes specific for NtPCLS5-1, NtPCLS5-2, NtPCLS6, NtPCLS7 and NbPCLS7, and N. tabacum ITS. Four plant species, B. rapa, Sol. tuberosum, N tabacum, and N. benthamiana were analyzed. NtPCLS5-2 possesses an internal EcoRI recognition site. No cross-hybridization was observed on Southern blots under the conditions used in this study between NtPCLS7 and NbPCLS7, which share 75% nucleotide sequence identity with 6 gaps between the sequences (compare sizes of PCR fragments in lane N. tabacum and the other lanes of the fourth panel of Figure S1B). (TIF) [file ppat.1002146.s001.tif]

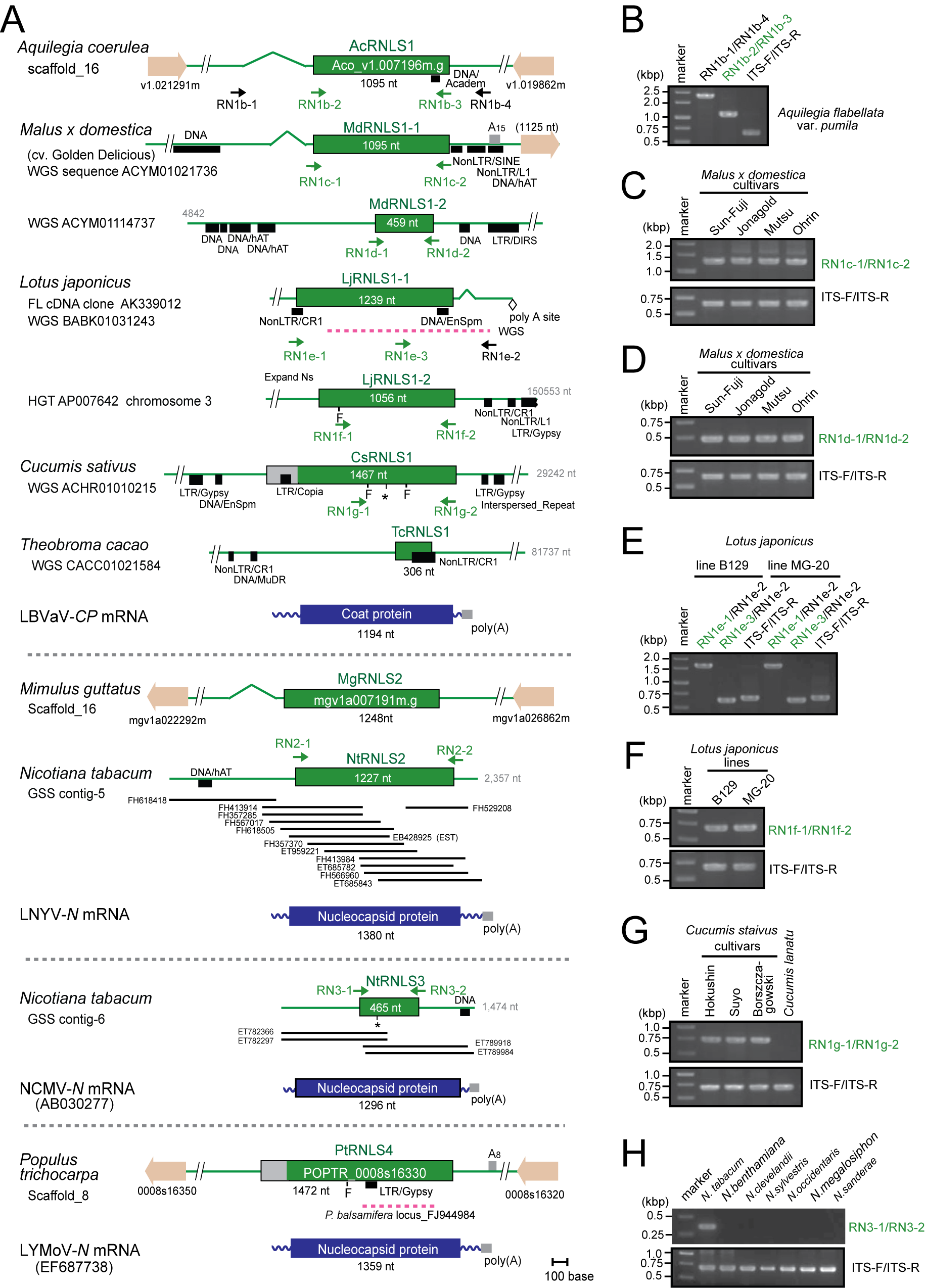

Supplement: Figure S3 — Schematic representation of chromosomal positions of RNLS s and their detection by genomic PCR. (A) Map positions of a total of 11 RNLSs are depicted. Their source plants, such as cucumber, apple, and N. tabacum are shown at the left. RNLS1s showed highest levels sequence similarities to the CP of LBVaV, while the other RNLSs are most closely related to the N protein of cytorhabdovirus, either lettuce necrotic yellows virus (LNYV), northern cereal mosaic virus (NCMV) or lettuce yellow mottle virus (LYMoV). Contigs were constructed from GSSs of N. tabacum as shown below the chromosomal positions of NtRNLS2 and NtRNLS3. Sequences related to transposable elements or repeat sequences are shown by black thick lines. See the legend to Figure 1 for explanation of the other symbols. (B–H) Molecular detection of RNLSs from several plants. Representative RNLSs from Aq. flabellata (B), Mal. domestica (C, D), L. japonicus (E, F), Cuc. sativus (G), and N. tabacum (H) were detected by genomic PCR and sequencing. Primers' positions and sequences are shown in A (arrows) and Table S3. Entire regions of RNLSs were amplified in all PCR assays (A to F), while in panels B and E partial forms of RNLSs were also amplified. Most sequences of DNA fragments were identical to those available from the respective genome sequence databases. (TIF) [file ppat.1002146.s003.tif]

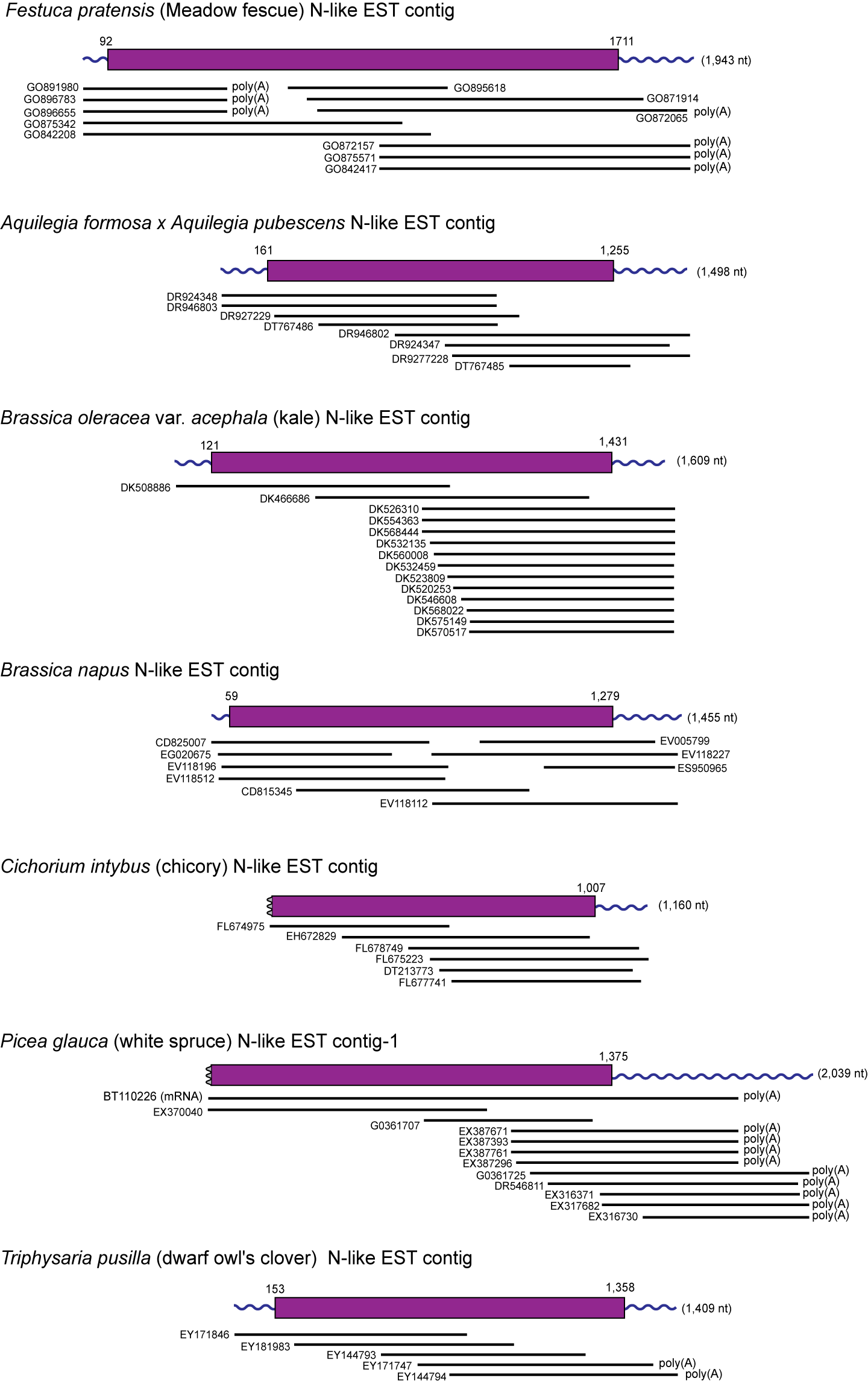

Supplement: Figure S4 — RNLS Contigs constructed from EST libraries of different plants. Rhabdovirus nucleocapsid (N)-like sequences were detected by searching EST databases for F. pratensis, Aq. formosa×Aq. pubescens, B. oleracea, B. napus, Cic. intybus, Picea glauca, and Triphysaria pusilla. Multiple ESTs were used to construct contigs where overlapping regions of EST sequences show over 99% sequence identity. These ESTs are either from endogenized viral sequences or infecting viruses. (TIF) [file ppat.1002146.s004.tif]
