# Supplementary material for: Widespread Endogenization of Genome Sequences of Non-Retroviral RNA Viruses into Plant Genomes
Source: PLoS Pathog. 2011 Jul 14;7(7):e1002146. doi: 10.1371/journal.ppat.1002146 (PMC3136472; doi:10.1371/journal.ppat.1002146)
Supplement: Figure S2 — Alignment of plant partitivirus CPs and their related plant sequences (PCLSs). The entire region of RnPV2 CP (aa 1–483) was aligned with homologous sequences from other plant and fungal partitiviruses, translated EST sequences of possible plant partitivirus origin, and plant PCLSs using the program MAFFT version 6. The alignment was used to generate a phylogenetic tree, as shown in Figure 4. For full virus names and information on PCLSs, see the Figure 4 legend, and Tables 1 and S4. Three relatively well conserved sequences, PGPLxxxF, F/WxGSxxL and GpfW domains are marked in red. (PDF) [file ppat.1002146.s002.pdf]

```
CLUSTAL format alignment by MAFFT (v6.851b)

RnPV2W57-CP     ------------MSTPS-----VTLSAFKAAVAKSGLEDQFLKLTGLTSMDELSADNFNE
FestucaEST      ------------MSSKTNASIESRLAAAKAAVRELGFEDQFLKQTGLTSLDELDPKQFDD
MgPCLS1         ------------M-----------------------------------------------
AtPCLS1         ------------M-----------------------------------------------
AtPCLS1_No      ------------M-----------------------------------------------
AsPCLS1         ------------M-----------------------------------------------
AtPCLS1_C24     ------------M-----------------------------------------------
AaPCLS1-A       ------------M-----------------------------------------------
AaPCLS1-B       ------------M-----------------------------------------------
CrPCLS1         ------------M-----------------------------------------------
CbPCLS1         MIFSLLRKRSITM-----------------------------------------------
OkPCLS1-A       ------------M-----------------------------------------------
OpPCLS1-A       ------------M-----------------------------------------------
OkPCLS1-C       ------------M-----------------------------------------------
CPCV-CP         ------------M------------------------------------SLSSGPVSQAV
FvBV-CP         ------------M-----------------------------------------------
SsPV-S-CP       ------------M-----------------------SSRSARKVSTQEKTSSQSGKNRAS
CCRS-PV-CP      ------------M---------------------------------SAPATTGSTTSTAS
ACD-PV-CP       ------------M---------------------------------SAPATTGSTTSTAA
WCCV-CP         ------------M-------------------------------NQDTPLANLNGPEVPS
CCV-CP          ------------M-------------------------------DDNTPAAQPNGPHAPA
BCV-CP          ------------M-------------------------------ENNTPLANPSGPNVPS
VCV-CP          ------------M-------------------------------EAHTPAADVNGPNIPS
BrPCLS4         ------------M-----------------------------------------------
BnPCLS4         ------------M-----------------------------------------------
BoPCLS4         ------------M-----------------------------------------------
RSCV1-dsRNA3    ------------MA--N-----PNNNQELLNVLAPHVEIPPPPPQQIGVADAIPVVAAQQ
BrPCLS5_Cc      ------------M-----------------------------------------------
BrPCLS5_tu      ------------M-----------------------------------------------
BnPCLS5         ------------M-----------------------------------------------
BoPCLS5         ------------M-----------------------------------------------
SpPCLS5         ------------MA---------QDGALPLQDAALNVNVPLNLFGFIGVADPARNMVAER
StPCLS5         ------------MA---------QDGALPLQDAALNVNVPLNLFGFIGVADPARNMVAER
SlPCLS5         ------------MA---------QDGALPXLDTALNVNLXLNLVGSIGVADPARNMVAEH
NtPCLS5-1       --------------------------------------------XGVGVANPTRDLVAER
RSCV1-CP        ------------MAHRT-----PTNAPALPQVDGINPNLPPNDPGTVAAAPNARNLHLER
HetRV3-ecl-CP   ------------M---------------------------SSIPDFASMNESDRLKYFEE
RSCV3-CP        ------------M-----------------------------------------------
MdPCL7          ------------MEDDSF------------------------------------------
BCV2ds1-CP      ------------M-----------------------------------------------
BCV2ds2-CP      ------------M-----------------------------------------------
FCCVds2-CP      ------------M-----------------------------------------ATDNSD
RoCVds2-CP      ------------M-----------------------------------------DKDAAA
RSCVds2-CP      ------------M--------------------------------------------AFE
MePCLS2         ------------M-----------------------------------------AANNPD
FCCVds3-CP      ------------M------------------------------------ASPRDSMMTTA
RoCVds3-CP      ------------M------------------------------------ASSKRTPPLVA
SbPCL8          ------------M---------------------------------KPTDATGKDAGTSA
RSCVds3-CP      ------------M-------------------------------------------DTAQ


RnPV2W57-CP     KFGQADSLTKSILA-----------------------PSAAPVPHQEQK---------KE
FestucaEST      SAPYAPTKLKPS-------------------------TSGAPKPSKELT---------RS
MgPCLS1         -----------------------------------------------------------A
AtPCLS1         ------------------------------------------------------------
AtPCLS1_No      ------------------------------------------------------------
AsPCLS1         ------------------------------------------------------------
AtPCLS1_C24     ------------------------------------------------------------
AaPCLS1-A       ------------------------------------------------------------
AaPCLS1-B       ------------------------------------------------------------
CrPCLS1         ------------------------------------------------------------
CbPCLS1         ------------------------------------------------------------
OkPCLS1-A       ------------------------------------------------------------
OpPCLS1-A       ------------------------------------------------------------
OkPCLS1-C       ------------------------------------------------------------
CPCV-CP         LTASEPAVPAPVEYRPGATQPDATG------------TMTQTASGTANAIFQSGATTAPE
FvBV-CP         -------SPPRFPKPPVTPSASQTADPLG--------TRDVDSESSKPKLVNSRPQKAVP
SsPV-S-CP       KKSGKKSAPPEYSVDTPSEQSDVESDILSELEVSDADDASRPVPASKSKKGTTRNKKKEK
CCRS-PV-CP      VPATEQTAPASVAVPPTAPATDVKY------------VAPAKKQKSFAP---------RE
ACD-PV-CP       VPATEQTAPATVAAPPTAPATDVKY------------VAPAKKQKSFAP---------RE
WCCV-CP         GNVPPANPPGRTNVAPPAQGAVQQ-------------PPAPAARRARNP---------HG
CCV-CP          AAAPHAVPPPAIPPATPAPGAVQQ-------------PPAPAPRRSRNP---------HG
BCV-CP          AAAPPTPAPPAIPQATPTPGAVSQ-------------PPAPPARRSRTP---------RG
VCV-CP          GAVSQPEIAPHNQAAPNVSGAAQA-------------IIAPTPARKRTP---------HG
BrPCLS4         --------------------------------------HPHASRHDRTP---------RG
BnPCLS4         --------------------------------------HPHASRHDRTP---------RG
BoPCLS4         --------------------------------------HPHASRRDRTP---------RG
RSCV1-dsRNA3    QQVIDQAANAAA--------------------------AQVARFEAEHA---------QA
BrPCLS5_Cc      ------------------------------------------------------------
BrPCLS5_tu      ------------------------------------------------------------
BnPCLS5         ------------------------------------------------------------
```

```
BoPCLS5          ------------------------------------------------------------
SpPCLS5          DQEAQRDRVVAI--------------------------AHCPRFEARSC----------L
StPCLS5          DQEAQRDRDVVAI-------------------------AHCPRFEARSC----------L
SlPCLS5          NQEAQRDRVIA---------------------------CHXPRFEARSC----------L
NtPCLS5-1        DQEAQRDGAEAA--------------------------AKRQRFQARRL---------QV
RSCV1-CP         EQEARQDRAAAA--------------------------FVPNTRFAVRRP---------TI
HetRV3-ecl-CP    QQKKQKELIDQSIKSKQTMSAMRVR------------PVFQQARNNVTSVNTSSSVPTKA
RSCV3-CP         ------------------------------------------------------ENGANNP
MdPCL7           ------------------------------------------------------------
BCV2ds1-CP       ------------------------------------------------------------
BCV2ds2-CP       ------------------------------------------------------------
FCCVds2-CP       AKTEQSKVTESKGSSPTKSLPAHAA------------DEKAAVGATFKLQSRQPTEPPAI
RoCVds2-CP       AQTEQSKITESHGSSPEKNAAAHTA------------DQKAAVGSTFQLQSRKPTEPPTV
RSCVds2-CP       SKTSASESTVNKDAAPAPSSA----------------SVKSPVKPSSSGLGRAKTETPHL
MePCLS2          ASADLSQETPKNTRVPSRGSA----------------AAIAPQTPSIGARKTEATTPPHL
FCCVds3-CP       QDAKKRKVSHILSPIRMIEPAAQSL------------AIIPVVPQV-------QMDDAEA
RoCVds3-CP       QQPKKRRFETVFDLGPSLGSTSLAM------------VHVPQPTPM-------LTDEAEQ
SbPCL8           DQSKRQATRQTLAMGPPTANDNPML------------ETLPELLDSVEP-----------
RSCVds3-CP       TDADKAAGKRGATVPPEGEPAAKTM------------RFSDAIVPAGPSKDASTVATAAL


RnPV2W57-CP      DADAKRVSAPVSASDF----------LYGFKHMT-IQYASRKKNNTFLPSAFMMMY-IIH
FestucaEST       DVDTSDDYTPESASDM----------LMPYLGLN-LRYVSRQRPSRYAPSSHMMDY-IVH
MgPCLS1          EASTGLNSTAAASDVM----------LRPYAGLHIITSASKKKPSTYRPSSLMMDY-IIH
AtPCLS1          -------ASESSTHKTKLEKKTCLSFLSSFTRSQ-IVYPTQG-ISHYYPSCHMM----VH
AtPCLS1_No       -------ASESSTHKTKLEKKTCLSFLSSFTRSQ-IVYPTQG-ISHYYPSCHMM----VH
AsPCLS1          -------ASESSTHKTKLEKKTCLSFLSSFTRSQ-IVYPTQG-ISHYYPSCHMM----VH
AtPCLS1_C24      -------ASESSTHKTKLEKKTCLSFLSSFTRSQ-IVYPTQG-ISHYYPSCHMMDY-IVH
AaPCLS1-A        -------ASESSTAKTKLEKKTSLSFLHGFTRGR-IVYPTQGRLSYYSPSCHMMDY-IVH
AaPCLS1-B        -------ASESSTAKTKLEKKTSLSFLHGFTRGR-IVYPTQGRLSYYSPSCHMMDY-IVH
CrPCLS1          -------ASESSAAKM-------LSFFYGVTGNR-IVHPTHGRLSHYYPSCHMMDY-VVH
CbPCLS1          -------ASESSAAKM-------LSFFYGVTGNR-IVHPTHGRLSHYYPSCHMMDY-IVH
OkPCLS1-A        -------ASESSIAKM-------LSLFYG-SGRR-FGYPNQDRLSHYYPSCHMMDY-IVH
OpPCLS1-A        -------ASESSIAKM-------LSLFYG-SGRR-FGYPNQDRLSHYYPSCHMMDY-IVH
OkPCLS1-C        -------ASQSSTAKM-------LSLFYG-SGSR-IVHPTQGGLSHYYPSCHLMDY-IVH
CPCV-CP          QPPVLIHVSDDAKSNE---KMSAIDITRIAICYP-MSTDSVLVPSWFLPSSRYLYR-IVQ
FvBV-CP          RAGPTSAPSVSDANPI----------YHAVSNFP-YYPHQRLDTSTFVPCTQVLFA-VLA
SsPV-S-CP        SVASSAGSSIPPYMMM----------LTALNFFS-LTEIEHVTISTYTPSCWSMYA-VLD
CCRS-PV-CP       PTATSGGPKNPGLSMM----------LSGVSDLP-FFGIKHNDVSYVVPDTTQLFY-VLS
ACD-PV-CP        PTASSAGPKNPGLSMM----------LSGVSDLP-FFGIKHNDISYVVPDTTQLFY-VLS
WCCV-CP          PVPPAGPSRSAGAPAM----------LELSAAYP-MYTEQRRAPNYYVPDAQLLFH-TLG
CCV-CP           PTPISSRVSAPSAPAL----------LELPAATP-MYTEQRRSANFFVPDAQMLFH-VLS
BCV-CP           PVPQATPGSTSGAPAL----------LELSAGLP-MYTVPRRGVNTFVPDAQMLFH-VLG
VCV-CP           PIPAATSSGNTSAPAL----------LELAAAYP-MYTEQRRSFNFFIPDSQMMFH-SLG
BrPCLS4          PVPQSPNHNIAPKNAM----------LEIAASQP-YITVHPPRFNSFVPDAQMLFH-VLG
BnPCLS4          PVPQSPNHNIAPKNAM----------LEIAASQP-YITVHPPRFNSFVPDAQMLFHSVLG
BoPCLS4          PVPQSPNHNIAPPNAM----------LEIAASQP-YTTVHPPRFNSFVPDAQMLFH-VLG
RSCV1-dsRNA3     QT--RAATAKNYSTLI----------CGPAAMIH-PVKDQYPEVSTFTPNFSTSYQ-YLN
BrPCLS5_Cc       ---------DNIHNAM----------CATAANFR-LTKVIYPAISTFTPCFISIFY-YLN
BrPCLS5_tu       ---------DNIHNAM----------CATAANFR-LTKVIYPAISTFTPCFISIFY-YLN
BnPCLS5          ---------DNIHNAM----------CATAANFP-LTKVTYPAISTFTPCFISIFY-YLN
BoPCLS5          ---------DNIHNAM----------CATAANFP-LTKVAYPAISTFTPCFISIFY-YLN
SpPCLS5          APPFRGLADPNIETAI----------LASASNFP-IQRITYPGESTFIPCFISVLY-YLN
StPCLS5          APPFRGLADPNIETAI----------LASASNFP-IQRITYPGESTFIPCFISVLY-YLN
SlPCLS5          APPFRGLADPIIETAF----------LAYASNFR-ILRITYPGESSFIPCFISILY-YLN
NtPCLS5-1        EP--PARHDPAIETAI----------AATAVNFP-LTRMYNPRVSTYIPCFATILY-YLN
RSCV1-CP         RP-SPAHSAPDLETAL----------FDSAVNHP-PTPVSYPSTSSYIPNFTSAFY-YLN
HetRV3-ecl-CP    VPPQPFSGTSSSANNM---------LRLAFGENP-FRPKQRFGQNGFVPDSQHLFA-LLS
RSCV3-CP         PAAGAIVAAVQPALPPGLDASKLNRATEYLYDAD-KIRPHRARSPKYRRYMQLKKNEIYV
MdPCL7           -------------------KDSLSRATKYALEAG-CIRLRRKE--RVTRCVNLHLQTIFD
BCV2ds1-CP       -TGPTQAHLIEATSFTGLNRDELDSIVDALQEEN-QIGTFRDRPKAYRRYVHVRSQELFD
BCV2ds2-CP       -----APNDELAANLQGLKRPALDEIVHYLEDRN-QIGISHDKNLSYNRTCVLLTNELHA
FCCVds2-CP       PS----VFAQSQSLNDSVGNPDFIAQLRDEGMYG-LFLPRDRARRTPTPVTLRPLT-YFQ
RoCVds2-CP       PS----AFAQSTALNVSVGNADYIAEQRDEGKYG-LFLPRDRAHREPEPVTMIVGN-FFD
RSCVds2-CP       PASNYEIPRYTRDTQPVVGNKNYEFSLRMDGRLG-HSMDRSKTARPEVNCSLNRAN-FHE
MePCLS2          FT----AFEVASEEEVVVGNPKLVFSCKQEGVLG-VFFDRDDSVVDPTPIIINGNN-MRN
FCCVds3-CP       MAELLYRERARIELDI---NPAYISRLESRREIG-FYPPVASKNVPFTTFNIDVAE-FRR
RoCVds3-CP       MAELLFRERSRIELDI---NPAYVSELELRREVG-FSPMIANKTLAFTTYDLRVVE-FRR
SbPCL8           ----------------------------------SYLNTATKELARQILEINFTE-LRR
RSCVds3-CP       PKDLIQWPIERLATQV---NQVFLDTTIQNGEGG-YEVPDDQIVPTLRVYEINVTN-LRN


RnPV2W57-CP      EMNVL------LCEHFYF--KREAPNY-----------------------HPLLLRTYFG
FestucaEST       LINDN------LCDNFYF--KRSCPDY-----------------------HPYILRLYYG
MgPCLS1          QTNDL------LVDHPHF--RRSSPDY-----------------------HPYILRLYYG
AtPCLS1          AMNIT------LCDNFDF--KRANPNY-----------------------HPYILRLYCG
AtPCLS1_No       AMNIT------LCDNFDF--KRANPNY-----------------------HPYILRLYCG
AsPCLS1          AMNIT------LCDNFDF--KRANPNY-----------------------HPYILRLYCG
AtPCLS1_C24      AMNST------LCDNFDF--KRANPNY-----------------------HPYILRLYCG
AaPCLS1-A        AMNIT------LCDNFEF--QRANPNY-----------------------HPYILRLYCA
AaPCLS1-B        AILLT------LHFVIILISNGPTPNY-----------------------HPYILRLYCG
CrPCLS1          SMNIT------LCDNFDF--KQANPYY-----------------------HPYILRLYCG
CbPCLS1          SINIT------LCDNFDF--KQANPYY-----------------------HPYILRLYCG
```

```
OkPCLS1-A        AMNTT------LCDNFDF--KWANPNY-----------------------HPYILRLYCG
OpPCLS1-A        AMNTT------LCDNFDF--KWANPNY-----------------------HPYILRLYCG
OkPCLS1-C        AMNIT------LCDNFDF--KWANPNY-----------------------HPYILRLYCG
CPCV-CP          EMDII------MLNTHRF--TQSVHAW-----------------------SPLVSRVYIA
FvBV-CP          HMDNI------MCTTYSW--TQSQPAW-----------------------HPCVSRLYFS
SsPV-S-CP        AMHDL------VGDNASL--RRFCPYY-----------------------HVALSNIYYG
CCRS-PV-CP       IMDTQ------MARTKRF--TDANPDW-----------------------HPFVSQLYIA
ACD-PV-CP        IMDTQ------MVRTKRF--TDANPDW-----------------------HPFVSQLYIA
WCCV-CP          VCDQL------MTTTDRF--LRSMPSW-----------------------LPIVSQLYVS
CCV-CP           ICDQM------MNSTERF--LRSSPAW-----------------------MPIVSQLYIS
BCV-CP           ICDQM------MLSTDRF--TRSSPAW-----------------------IPIVSQLYIS
VCV-CP           ICDSM------MNSTDRF--LRSSPAW-----------------------LPIVSQLYIS
BrPCLS4          ICDQL------MLTTTQF--IRSSPSW-----------------------LPIVSQLYIS
BnPCLS4          ICDQLMLTTTSMLTTTQF--IRSSPSW-----------------------LPIVSQLYIS
BoPCLS4          ICDQL------MLTTTQF--LRSSPSW-----------------------LPIVSQLYIS
RSCV1-dsRNA3     LMDQS------MISTKRW--TDNCLGW-----------------------APPYSQIYIG
BrPCLS5_Cc       SMDHL------MTSTKRW--TNNCMDW-----------------------VPPYSQIYIA
BrPCLS5_tu       SMDHL------MTSTKRW--TNNCMDW-----------------------VPPYSQIYIA
BnPCLS5          SMDHL------MTSTERW--TNNCMGW-----------------------VPPCSQLYIA
BoPCLS5          SMDHL------MTSTERW--TNNCMGW-----------------------VPPCYQLYID
SpPCLS5          QMDHL------MVSTKRW--TDNCMGW-----------------------VPPYSQIYIS
StPCLS5          QMDHL------MVSTKRW--TDNCMGW-----------------------VPPYSQIYIS
SlPCLS5          QMDHL------MVSTXRW--TDNCMGW-----------------------VLPYSQIYIS
NtPCLS5-1        CMDFL------MAATKRW--TDNCLGW-----------------------VSPYSQIYIS
RSCV1-CP         KMDSL------MVQTLNW--TNNCSGW-----------------------VPPYSQIYIS
HetRV3-ecl-CP    VMDAK------MASTRKF--VEGCEFW-----------------------TPLVSQYYLS
RSCV3-CP         QLIDI------YRASFSV-------TWPDFRHLI-HAFPPPANHEP----QTYYACCYIS
MdPCL7           QLVSL------YIPLFQC-------QWRRLKYLFEESALLPSGVTP----WIHATRCYIS
BCV2ds1-CP       RLSEL------YADLFTS-------NWSAFKHAYITEPTGREDANSISTKASFMAQCYLT
BCV2ds2-CP       RLVTL------YTNLFTT-------SWRHFKSAV-RKEMPRTEANP----QRWLANCYIT
FCCVds2-CP       SIERT------ISQSMFH-------LLQTKTEFNNDDAIQRINTIAEQLAIGACLATHMK
RoCVds2-CP       GIVRT------TERAMVY-------LLQKKSEFNDIDVLQRVHAVAEQLAIGAMLATYFK
RSCVds2-CP       GVLKV------LRQILSE-------MARLKQDLSDAEIQERVESVSLTLAYGVATMAYLK
MePCLS2          AVIAT------LTTVLTR----------ALKDFGVTSVHTMALETAATLANGVMIATFLK
FCCVds3-CP       CITHV------LTAALKV-------RYYDRSNLRNAEINTEINTIVPRTVSACMCAVYAK
RoCVds3-CP       CITAV------LTGALKA-------RYFDRSNLRQAEITAEINTIVPLTVSACMCALYSK
SbPCL8           CMANV------LNHSLTI-------LYFDSGSMRDADINTELKVIVPVTIVCAXTALYAK
RSCVds3-CP       AFRSV------LYTILRA-------KFIERGDRTVTAATTLATNVSNLTADACMAALYAK

                                                                          .
                                                              PGPLx   x xF
RnPV2W57-CP      ILFIIQTLRAQHAANN--LFGDQF--EFLEKFLDAYP---VDTLPIPAPLI---H-IFK-
FestucaEST       VLFWIQCLRAARDVRV--LEDEEY--QFLMRFLDAYP---LESLTISSPLL---V-IFK-
MgPCLS1          ILFWIQCLRAAAAVQA--LNSDQH--QTLTRFLSAFP---PDSLTISSPLL---Y-LFK-
AtPCLS1          VLFWIQCLRAGNDVND--LTDVQH--RFLNRFLDNHP---LETLAVPGPLL---G-LFK-
AtPCLS1_No       VLFWIQCLRAGNDVND--LTDVQH--RFLNRFLDNHP---LETLAVPGPLL---G-LFK-
AsPCLS1          VLFWIQCLRAGNDVND--LTDVQH--RFLNRFLDNHP---LETLAVPGPLL---G-LFK-
AtPCLS1_C24      VLFWIQCLRAGNDVND--LTDVQH--RFLNRFLDNHP---LETLAVPGPLL---G-LFK-
AaPCLS1-A        VLFWIQCLRAGNDVNA--LTDVQH--RFLNRFLDNHP---LETLAVPSPLL---R-LFK-
AaPCLS1-B        VLSWIQCLRAGNDVNA--LTDVQH--RFLNRFLVNHP---LENSRCSRALS---SDSFK-
CrPCLS1          VLFWIQCLRAGNNVNA--LTDVQY--RFLNRFLNNHP---LETLAIPGPLL---E-LFK-
CbPCLS1          VLFWIQCLRAGNNVNA--LTDVQY--RFLNRFLDNHP---LETLAIPGPLL---E-LFK-
OkPCLS1-A        VLFWIQCLRAGNDVHA--LTDDQH--KFLNRFLDNYP---VETLVVPCPLL---E-LFK-
OpPCLS1-A        VLFWIQCLRAGNDVHA--LTDDQH--KFLNRFLDNYP---VETLVVPCPLL---E-LFK-
OkPCLS1-C        VLF-IQCLRAGNDVHA--LTHDEH--KFLNRFLDNYP---METLVVPGPLLGRPE-FPKH
CPCV-CP          VLFWIQTIRCMQATGF--LPREGI--AFINELLQDFP---ARTLVVPGPLV---S-LFR-
FvBV-CP          TLVYYQILRAMDAARI--ITVLQK--ELLNFLEHTIP---ASALPIPGPLA---P-FLR-
SsPV-S-CP        IIFIIQVLRANQVANN--LSQADF--QFLRFFESNFA---LEELPVAGPLV---L-FFQ-
CCRS-PV-CP       VLFYYQVLKNQTHGGM--ISNDQR--LFVDFLDTQFK---AEHLKIPGPIA---I-FFQ-
ACD-PV-CP        VLFYYQVLKNQSHGGM--ITNDQR--LFVEFLDSQFK---AEHLKIPGPIA---I-FFQ-
WCCV-CP          VLWNVMILKVYVNTGY--GAAYAH---DLDVLLNHLQ---INECMIPGPLV---P-FFQ-
CCV-CP           VLWNVMTIHVFVNSGY--GSAFSS---FLTELYSVLR---IDECMIPGPLV---P-FFQ-
BCV-CP           VLWMVAILRVFVASGY--GALYSS---LINDLIGHLR---IDECMIPGPLV---P-FFQ-
VCV-CP           VLWNVMIIRILSHTGY--APSFTD---LLNTLTTDLQ---IEECMVPGPLV---P-FFQ-
BrPCLS4          LLWNFTILRNFVSSRL--GSFSQY-YQILNELEF------LKHCIVPGPLV---S-FFQ-
BnPCLS4          LLWNFTILRNFISSRQ--GSFFQY-YQILNELEF------LKHCIVPGPLV---S-FFQ-
BoPCLS4          LLWNFTILRNFVSSRL--GSFFQY-YQILNELEF------LSXCIVPGPLV---S-FFQ-
RSCV1-dsRNA3     ILGYIQTMRAMNAAGL--LEPNSEISRLLGNFMKVFP---LKSLWIPGPLV---S-FFK-
BrPCLS5_Cc       MLLYIQTMRAMEASGY--LSRGSELSLLLGDFCNQFP---LQNLWIPGPLV---Y-AFK-
BrPCLS5_tu       MLLYIQTMRAMEASGY--LSRGSELSLLLGDFCNQFP---LQNLWIPGPLV---Y-AFK-
BnPCLS5          MLLYIQTMRAMQASGY--LSPGSEISSLLGDFCYLFP---LQNLWIPGPLV---D-AFR-
BoPCLS5          MLLYIQTKRAMQASGY--LSPGSEISSLLGDFCYLFP---LQNLWIPGPLV---D-AFR-
SpPCLS5          ILLYIQVIRAMDSAGI--FRFGSRLSIFLREFETLFP---FTNLWIPGPLV---S-AFK-
StPCLS5          ILLYIQVIRAMDSAGI--FRFGSRLSIFLREFETLFP---FTNLWIPGPLV---S-AFK-
SlPCLS5          ILLYIQVIRGIDSAXI--FRIGSRLPIFLRELETLFP---FTNLWIPGPLV---S-TFK-
NtPCLS5-1        VLFYIQVMRAMESSDT--LRLGSDISLFLREFLILYP---LEDLRVPGPLV---Y-GFK-
RSCV1-CP         MLLYLQVMRAMKKAGV--LRPNSELSHLFNEMSTIFP---FESLMVPGPLV---N-LFE-
HetRV3-ecl-CP    VLIFIQVARAKHEAGL--LSGELA--DVYDLFCGHSPAINLNALPVPGPFL---N-MLS-
RSCV3-CP         GWFRDLYYHIRKALKS--LSGLAE----LEYYRDPIP-----------QMSFEYDNFLA-
MdPCL7           AWIHDLYVSNREAVQT--LCSLTF----LKHYSHEEV-----------YCSSEYDAFLI-
BCV2ds1-CP       AWFWDTQYCIRESVRK--LSGTAY----NQHFTEDLN-----------LISPKYDPFLQ-
BCV2ds2-CP       AWFWDLQASIQEATKT--LSGKIY----QDYFRDNIH-----------PILDRYDPFLQ-
FCCVds2-CP       -LRALIHLDFPDRYNIGSLKRPKT-----------------ITDLPVVAPFA---F-AIQ-
RoCVds2-CP       -LRALIYLDLPDRYTLGSLKRPKA-----------------IADLPVIAPFA---F-AIQ-
```

```
RSCVds2-CP      -LRAINLFRPNEASKF--LTKPKV----------------PDHFEIPTPFA---F-AIS-
MePCLS2         -LRSVGYLNSSLMSRF--YVVPRT----------------PQPFELPLPYA---L-AIS-
FCCVds3-CP      -LRSIHRTHGTLRNRY--VTTPTY----------------NKDIELPLPLA---I-AIQ-
RoCVds3-CP      -LRSIHRTHGRLSARY--AAAPHY----------------NKDIELPLPLA---V-AIQ-
SbPCL8          -LYNIHHDYGVHPSRY--LTPPSF----------------AKEIELPLPLA---L-LIE-
RSCVds3-CP      -LRSLHRQIYTHKGRF--TDQPTY----------------TKDVELPLPFA---V-AID-


RnPV2W57-CP     -SLCSS-R-PEIP--QYGYV--TPFVPEEL-WGTQLKDSPANALY-A---RNFFPNIPYI
FestucaEST      -TLCSS-Q-PEFP--TYGKV--YPALPSLP--GPTRRDEFMHEHV-D---NFFVPNIPGI
MgPCLS1         -TLCSSPPPPENP--SYGKV--YPLLPSPP--GPTARSAFMSNSP-Y---HYIIPNIPGI
AtPCLS1         -TLCSS-Q-PEFPHNHNGKV--YPRIPAQP--GPARRDAFMRDLL-E---SHFLPNVPGI
AtPCLS1_No      -TLCSS-Q-PEFPHNHNGKV--YPRIPAQP--GPARRDAFMRDLL-E---SHFLPNVPGI
AsPCLS1         -TLCSS-Q-PEFPHNHNGKV--YPRIPAQP--GPARRDAFMRDLL-E---SHFLPNVPGI
AtPCLS1_C24     -TLCSS-Q-PEFPHNHNGKV--YPRIPAQP--GPARRDAFMRDLL-E---SHFLPNVPGI
AaPCLS1-A       -TLCSS-Q-PEFPHNHNGKV--YPRIPAQP--GPARRDAFMRDLL-E---SHFLPNVPGI
AaPCLS1-B       -TLCSS-Q-PEFPHNHNGKV--YPRIPAQP--GPARRDAFMRDLL-E---SHFLPNVPGI
CrPCLS1         -TLCSS-Q-PEFP--HNGKV--YPQIPAQP--GPARRDAFMGNLL-E---SYFLPNVPGI
CbPCLS1         -TLCSS-Q-PEFP--HNGKV--YPQIPAQP--GPARRDAFMGNLL-E---SYFLPNVPGI
OkPCLS1-A       -TLCSS-L-PEFH--YSGKV--YPRILAQP--GPARRDSFMRCHL-E---SYFLPNVPGI
OpPCLS1-A       -TLCSS-L-PEFH--YSGKV--YPRILAQP--GPARRDSFMRCHL-E---SYFLPNVPGI
OkPCLS1-C       GHSCSS-Q-PEF-----------PCIPSQP--GPARRDSFMRCHL-E---SYFLPNVPGI
CPCV-CP         -SLCAS-S-PSFG--TYGDV--FPAFETAG-IGASPANCFTLNSARFPQWYMLMPNVALL
FvBV-CP         -SPFLLFN-------WHGWL--LQCMSNHTXWPHHQKIKYNISSA-F---NNLLPNLVHA
SsPV-S-CP       -NLAAF-K-PDGN--RFNWV--VPHYNNYG--PANGANSPRVNAQ-----STALPQLPQM
CCRS-PV-CP      -SLAAN-A-GPNE--NFGNL--VFGIPNAH---DISCTSYLWQDK-V---HTILPNVIFI
ACD-PV-CP       -SLAAN-A-GPNE--NFGNL--VFGIPNAH---DINCTSFLWQDK-V---HTILPNVIFI
WCCV-CP         -SLAAV-N-GPFD--WIGDI--IPAMPSFT--ELW-TDEFAPHAA-Y---ARQIPIPAIL
CCV-CP          -ALGAV-N-GPFD--WIGDI--VPGLPNFS--ALWNEAGFHANNN-Y---VRQVPIPAVI
BCV-CP          -SLGAV-C-GPYE--WIGDI--VAAFPDFL--TLWDAENFCPTAD-L---ARTCPVPAIM
VCV-CP          -SLAST-N-GPFD--WIGDI--TPALPGFD--SLWDATNFTPHAS-Y---TRFVPIPAIL
BrPCLS4         -SLSSF-N-GRFF-----DI--TPIIPDFT--SLWNASAFHINAD-Y---ARQIPITAII
BnPCLS4         -SLASF-N-GRFF-----DI--TPIIPDFT--SLWNASAFHINAD-Y---ARQIPITAII
BoPCLS4         -SLASF-N-GRFF-----DI--TPIIPEFT--SLWNASAFHINAD-Y---ARQIPIPAII
RSCV1-dsRNA3    -SIACF-KPSASE--KHGMV--SPTLPTRP--GWSRNRRYRIVTD-A---SSHLPNINIF
BrPCLS5_Cc      -SVSCF-SPSASG--RFGNV--TPALPATP--GWSRARRYRIADA-A---TTHLPNINIF
BrPCLS5_tu      -SVSCF-SPSASG--RFGNV--TPALPATP--GWSRARRYRIADA-A---TTHLPNINIF
BnPCLS5         -SVACF-SPSASR--RFGNI--TPALPSSP--GWSRARRYRIADA-A---TTHLPNINIF
BoPCLS5         -SVACF-SPSASR--RFSNV--TPAIPSSP--GWSRARRYRIADT-G---TTHLSNINIF
SpPCLS5         -NLSCF-WPSATG--QFGNV--SPSLPATP--EWTQANRFSFANR-F---ASHLPNISIF
StPCLS5         -NLSCF-WPSATG--QFGNV--SPSLPATP--EWTQANRFSFANR-F---ASHLPNISIF
SlPCLS5         -NLSCF-WPSATG--QFGSV--SPSLPXTP--KWTQAHRFSFGSR-F---ASHLPNISIF
NtPCLS5-1       -NLSCF-WPSADN--SFGNV--SPPLPAGP------DRRYAFNNA-F---ASHLPNISLQ
RSCV1-CP        -NITAF-RPLQTD--SFGNV--TPFLPAEP--GWSNATFFAPNGS-L---VRHLPHIPAL
HetRV3-ecl-CP   -QVAAH-I-PHLV--DMDNI--CPIVPNNL--EATNTSYYLYTGQ-CQNLRGRLPNVPYI
RSCV3-CP        -LLGAA-IRPTHIVGNPEDVMYVPLISEAT---DVNQVNPFGINN-Y---VHQPELFSAI
MdPCL7          -HLNAD-IRPTHIKGTPEDTVYVPVLSDSP---AWGTANPFNIEN-F---VLDRNLVRGI
BCV2ds1-CP      -HLNMV-IRPTLIHQSTEDTLYIPLLGQTF-NYNEDAYNFLNLQG-C---GTEIRQVYAI
BCV2ds2-CP      -HLNTI-IKPTHIVNATEDVLYFPIISADY-RRADADMNIHRITG-A---FTRPNVVMDL
FCCVds2-CP      -QLGYV-NIANLT--EERRY--VPVLPET-------------------------------
RoCVds2-CP      -QLGYV-NVANLT--RERRF--VPVFPED-------------------------------
RSCVds2-CP      -QLGVV-EVSSLS--RRMIC--YPTADLAD------------------------------
MePCLS2         -QLGAV-KTHGSP--QEGYY--CPTIPAT-------------------------------
FCCVds3-CP      -GLGAF-RTESIL--ANRVI--APTYPE--------------------------------
RoCVds3-CP      -EFGIF-KTESMI--QNRIM--APTYPE--------------------------------
SbPCL8          -SFGAF-ETSCLT--TNYLY--IPTYPG--------------------------------
RSCVds3-CP      -GIGMF-RTSAMS--TRFNV--VPVYPE--------------------------------


RnPV2W57-CP     LSLYATLNSSTIKGS-------VKEMNSYLGNGTDDRTFAGHAYDKDPAQW---------
FestucaEST      IALLAHLNSIINTADPRNAVFPRKGKHIPVTANAAQATVFGHHSFPIAAER---------
MgPCLS1         LALLSDLNSTINDSNN-NPVYPGKGKHVPVADNRNEPVVFAHHRFPPTVDM---------
AtPCLS1         FALLEDLTXLFTQXPP---VYPKRGRHIPVTQNT--VSNFGHKTFGLHASR---------
AtPCLS1_No      FALLELNRXLFTQXPP---VYPKRGRHIPVTQNT--VSNFGHKTFGLHASR---------
AsPCLS1         FALLELNRXLFTQXPP---VYPKRGRHIPVTQNT--VSNFGHKTFGLHASR---------
AtPCLS1_C24     FALLEDLTXLFTQXPP---VYPKRGRHIPVTQNT--VSNFGHKTFGLHASR---------
AaPCLS1-A       FALLEDLNRLLSQYPP---VYPKRGRHIPVTENA--VSTFGHKTFGLHASR---------
AaPCLS1-B       FALLEDLNRLLSQYPP---VYPKRGRHIPVTENA--VSTFGHKTFGLHASR---------
CrPCLS1         FALLEDLNRLFSQYPP---VYPKRGRHIPVTEDA--VSTFGHKTFGSHANR---------
CbPCLS1         FALLEDLNRLFSQYPP---VYPKRGRHIPVTEDA--VSTFGHKTFGSHANR---------
OkPCLS1-A       FALLEDLNRLFSQYPP---VYPKRDRHIPATEDA--VSTFGHKTFGPHAVR---------
OpPCLS1-A       FALLEDLNRLFSQYPP---VYPKRDRHIPATEDA--VSTFGHKTFGPHAVR---------
OkPCLS1-C       FALLEDLNRLFSQYPP---VYPKRGRHIPVTEDA--VSTLGHKTFGPHAVR---------
CPCV-CP         LDQLTCFTQACVNNPA-----------------TIGDFVIGDDLFGTPFDG---------
FvBV-CP         VDSAYSFATDATY------VFRSAVNDLNVKMEATDPDMPAAET----------------
SsPV-S-CP       ISLLNLFGASNAALLT---AMDTAGQWEPFTFAAGGTIAGFAYGAGFATDA---------
CCRS-PV-CP      LDQFMRLISLISPVN-----------SGPVQANASHTDTVYTTIFGAPASK---------
ACD-PV-CP       LDQFMRLISLISPVN-----------SGPVQANASHTDTVYTTIFGAPASK---------
WCCV-CP         LDQLYRFATLAFDAQLQTNYATFEWYSNIFNQDVN-------------------------
CCV-CP          LDQLHYFSQYAVPANQQSTYTTFQWYRNVFQQGLG-------------------------
BCV-CP          LDQLHYFATWTIPA-EQILYTNFQWYRNIFSLGLG-------------------------
VCV-CP          LDQLYYCATYVGVN-QGDLYPTFTWYRNIFTRTGA-------------------------
BrPCLS4         LDQLHHFAT-----SDDTDSFQFQWYGNVFSQSSQ-------------------------
```

```
BnPCLS4          LDQLYHFAT-----SDDTDSFQFQWYGNVFSQTIQ-------------------------
BoPCLS4          LDQLHHFAT-----SDDTDSFQFQWYGNVFSQSIQ-------------------------
RSCV1-dsRNA3     ISRLRSICAAASRNGA---TEDSFLRDID-------GPQYLATLFAQPCSH---------
BrPCLS5_Cc       ISRLNSVCAAATRPNV---ASQLFLSDVD-------GPNYMANLFNQPCDQ---------
BrPCLS5_tu       ISRLNSVCAAATRPNV---ASQLFLSDVD-------GPNYMANLFNQPCDQ---------
BnPCLS5          ISRLNSICAAATRPNV---TPEIFFRDVD-------GPYYMANLFSQPCDQ---------
BoPCLS5          ISRLNFICAAATRPNV---TPKIFFRDVD-------GPNCMASLFSXPCDK---------
SpPCLS5          ISRLHSICSIATRPAV---TEHLICNDVD-------GPRFMATLFSQACVH---------
StPCLS5          ISRLHSICSIATRPAV---TEHLICNDVD-------GPRFMATLFSQACVH---------
SlPCLS5          ISRLHSICSITTRPAV---TEHLFCN-VD-------GPRFMATLFSQACVH---------
NtPCLS5-1        ISKLRSTAAAXLRAAT---TETLFFNDVD-------GPCRIATLFGTACNH---------
RSCV1-CP         ISRLRRICETASENGL---NDISFSAHHH-------GPEFISELFGHICDN---------
HetRV3-ecl-CP    LDQLHQLGVLLTANPV---GIDVANQGRVLHHSFLGVPMFDATVAPGNAAQWNSRVAFDA
RSCV3-CP         VATMKDRKKFN-------------------------------------------------
MdPCL7           IANMKRYWR---------------------------------------------------
BCV2ds1-CP       TDVMDSRRTEWS------------------------------------------------
BCV2ds2-CP       VSLMDDPNSGWS------------------------------------------------
FCCVds2-CP       ------------------------------------------------------------
RoCVds2-CP       ------------------------------------------------------------
RSCVds2-CP       ------------------------------------------------------------
MePCLS2          ------------------------------------------------------------
FCCVds3-CP       ------------------------------------------------------------
RoCVds3-CP       ------------------------------------------------------------
SbPCL8           ------------------------------------------------------------
RSCVds3-CP       ------------------------------------------------------------


RnPV2W57-CP      --T-EERAFFLRSPGMEHALEGTSSFNKDFFENRFDNLE--VPIPSTND--KVK------
FestucaEST       --S-NFEKWSLVSSGLQYPCEADQRLNETFAE-RYESFN--FPATLATD--DLR------
MgPCLS1          --T-DKDRWMLASSGLQYPCEADEKLHKGFAQ-RYENFS--FPTAVADDDEDLS------
AtPCLS1          --T-EAEKWSLVLPGLQYPCEADQSLNEAFAD-CYSNFN--FQVTSAAD--NLE------
AtPCLS1_No       --T-EAEKWSLVLPGLQYPCEADQSLNEAFAD-CYSNFN--FQVTSAAD--NLE------
AsPCLS1          --T-EAEKWSLVLPGLQYPCEADQSLNEAFAD-CYSNFN--FQVTSAAD--NLE------
AtPCLS1_C24      --T-EAEKWSLVSPGLQYPCEADQSLNEAFAD-CYSNFN--FQVTSAAD--NLE------
AaPCLS1-A        --T-EAEKWSLVSPGLQYPCEADQKLNEAFAD-RYSDFD--FQVTTAAD--NLE------
AaPCLS1-B        --T-EAEKWSLVSPGLQYPCEADQKLNEAFAD-RYSDFD--FQVTTAAD--NLE------
CrPCLS1          --T-EAEKWSLVSPGLQYPCEADQKLNEAFAE-CYNDFD--FQVTAATD--NLE------
CbPCLS1          --T-EAEKWSLVSPGLQYPCEADQKLNEAFGE-CYNDFD--FQVTAATD--NLE------
OkPCLS1-A        --T-EDEKWSLVSPGXQYPCEADQKLNEAFAE-RYSDFD--FQVTAATD--NLE------
OpPCLS1-A        --T-EDEKWSLVSPG-QYPCEADQKLNEAFAE-RYSDFD--FQVTAATD--NLE------
OkPCLS1-C        --T-EDEK-SLVSPGLQYPCD-DQKLNEAFSE-RYNGFD--FQVTAATD--NLE------
CPCV-CP          --TIGSHRQQRVAPGMTEPVYFNTKAAENHAS-MRNLMA--LPTRLNYDPQNATP-----
FvBV-CP          ----RHWNSAMMDPVYRNDMYIPTRVAQNFAA-FSAPLG--LPLRYNYGPDTDI------
SsPV-S-CP        ----AASDQTLRAPGINVPWQHDTAIHRKLVP-ISRRMS--VPTMNGAKD----------
CCRS-PV-CP       --D-EATRFAMLTPSARSDFQTTVGLLNGLST-SSNVWRNTLPFANDGDSQYVITEDDEL
ACD-PV-CP        --D-EATRFAMLTPSARSDFQTTVGLLNGLAT-SSNVWRNTLPFANDGDSQYVISDDDDT
WCCV-CP          ----THNARLRLGPQLCGSLFTTQAQCDSARAFWNPAFANGFTRIDAANG--PLM-----
CCV-CP           ----AMNRLNRIGPQLCGSLFTTQAQYDAARNFWNASLNAGITRANANEGQPAFS-----
BCV-CP           ----AGNANNRIGPQLCGSLYSPRAQVDSARAFWNAALSSGITRTNAAEANGAFY-----
VCV-CP           ----NTPALLRMGPNLCGSLFTTSNQFDAARTYWRACFGTGFTRVNVTAA--AFT-----
BrPCLS4          ----GYNKLNRIGPQLCGSLFSTPQQTASARAFWSSVFA-GATRVNAADETARFT-----
BnPCLS4          ----EYNKLNRIGRQLCGSLFATPQQTASARAFWSSVFS-GATRVNAADKTATFT-----
BoPCLS4          ----EYEPH---GPQLCGSLFSTPQQTASARAFWSSVFV-GATRVNAAGETAPFT-----
RSCV1-dsRNA3     --T-ASEIANLSSPGSNLSYSGDLRLWKLASA-RLSFVG--PLALLDSAVTDVSD-----
BrPCLS5_Cc       --S-ENIQANLTSPGASLTYSGSLRLWQDANH-QLPFLG--IPKSLYVNDTEVDD-----
BrPCLS5_tu       --S-ENIQANLTSPGASLTYSGSLRLWQDANH-QLPFLG--IPKSLYVNDTEVDD-----
BnPCLS5          --S-ENEQANLTSPGACLTYSGSLRLWQDANY-QLLFLG--SPQSLDVNATEVDD-----
BoPCLS5          --S-DHELANLTSPGACLTYSGSLRLWQDAND-RLLFLG--SPQSLDVNATKVGD-----
SpPCLS5          --D-ENXLINVASPGASLTYSGGLRLWQNAAA-NLSFYD--LPSPLDISVDHVEN-----
StPCLS5          --D-ENXLINVASPGASLTYSGGLRLWQNAAA-NLSFYD--LPSPLDISVDHVEN-----
SlPCLS5          --D-ENNXISVASPGASLTYAGGLHLWXNAAA-NLSFYD--LPSPLDISVDHVEN-----
NtPCLS5-1        --S-ANELINLASPGFSFQATGGLRLWQDAAN-SLSFLN--LPANLNITEDAVQD-----
RSCV1-CP         --D-LPEQLLLLTPGLATSYSGTLYLWRQARS-QLQRSL--FPEALTVN-DVVPN-----
HetRV3-ecl-CP    GFT-ASGSFIAVDPAARHPFFLNRGLLSQITD-YAALIN--VTRPARQNAAAVTP-----
RSCV3-CP         --MVPLPSNYSGRPSWLFDWHAEDQICAP------------FQPEGNFNNEDIAM-----
MdPCL7           --TSTVGSDNLGRPFXLLDWHEDCECCSW------------FPREGNYTMEDVMV-----
BCV2ds1-CP       --TIPLATNVLGRASWLLDFKQGNAYAW-------------FPFESNFTELDLVA-----
BCV2ds2-CP       --TVPLNTNVFGRPGWLLDYDGTDAYAW-------------FPMENNYNMCDLIA-----
FCCVds2-CP       ----GHTFGIPTGHNWNPNLYA--QAVDYARK---------FGLHFNVVDYTKKQ-----
RoCVds2-CP       ----DHTFGVPLEHHWNPNLYS--QAVDYART---------LGLHFNVVDYKKKQ-----
RSCVds2-CP       ----ASNHLVGNKRNWSQTAYA--EAVRYAKY---------LGMSFSTVDLDIKV-----
MePCLS2          ----TSNFLLPAGISWNPPAYS--RAVEYAKS---------IGLKFTQPDLDVQM-----
FCCVds3-CP       ----ATQYEGRAQDAFNMTEYQ--NYIPTLKD---------LGIPCKSVEIHDKK-----
RoCVds3-CP       ----ATQYEGRAQDNFNITDYQ--TYIPTFKD---------LGIPCKSVDPHIKS-----
SbPCL8           ----NTQHEGYANAEYPIQLLGICHMLQPSEN---------XKVPMKAFEPHHKS-----
RSCVds3-CP       ----NTKNEGRSHDSINFLEYK--SYLAYFAE---------IGIPTRTIDTRVTP-----

                                                                    F/WxGSxxL
RnPV2W57-CP      -FVDQFCYL------S-KNLAW-----------------FRDLVEIASAAAKFFEGSGTL
FestucaEST       -FLSSFLSM------N-GDLSW-----------------FAQVRDVAAAEADRFNGSGTL
MgPCLS1          -TLERFFST------F-DSLSW-----------------FARVREVAAVEAAYFEGSGTL
AtPCLS1          -KISSFLHM------K-HSMAW-----------------FNQVKGVADDVAASFEGSGTL
AtPCLS1_No       -KISSFLHM------K-HSMAW-----------------FNQVKGVADDVAASFEGSGTL
```

```
AsPCLS1         -KISSFLHM------K-HSMAW-----------------FNQVKGVADDVAASFEGSGTL
AtPCLS1_C24     -KISSFLHM------K-HSMAW-----------------FNQVKGVADDVAASFEGSGTL
AaPCLS1-A       -KISSFLHM------K-RSMAW-----------------FSQVKGVADDVAASFEGSGTL
AaPCLS1-B       -KISSFLHM------K-RSMAW-----------------FSQVKGVADDVAASFEGSGTL
CrPCLS1         -SISSFLHL------K-HRMTW-----------------FTQVKGVADDVAASFEGSGTL
CbPCLS1         -SISSFLHL------K-HRMTW-----------------FTQVKGVADDVAASFEGSGTL
OkPCLS1-A       -SISSFLHM------K-HNMAW-----------------FTHVIGVANDVAASFVGSGTL
OpPCLS1-A       -SISSFLHM------K-HNMAW-----------------FTHVIGVANDVAASFVGSGTL
OkPCLS1-C       -SISSFLHM------K-HNMAW-----------------FTQVIGVANDVAASFEGSGTL
CPCV-CP         LTLNEMFRF-------NTDKRW-----------------FSRIISTMTVYSKFFDGSVTL
FvBV-CP         -GLSHFLYL------SGGKHDY-----------------LNRLAPVFARYSQYFVNSKSL
SsPV-S-CP       -SPRTYCGL-------DGQFHW-----------------FHKAIGAVSQEAKYFAGSTTL
CCRS-PV-CP      LDLDQVFGFRGIGPYADRPYGW-----------------FAQVIRVMQPYSDFFKDTVSL
ACD-PV-CP       LDLDQVFGFRGIGNHADRPYGW-----------------FAQVIRVMQPYSDFFKDTVSL
WCCV-CP         -AFPQLLGFISQDGAL--QSNW-----------------FMHISLIMHKYAQYFNGSVPL
CCV-CP          -NYRQLFGFESQTGVL--QVNW-----------------FTSVSIAMNKYTQFFNGSVPL
BCV-CP          -TYAQLLGFISQNGTL--QLDW-----------------FQQVAVVMQKYTQYFNGSTPL
VCV-CP          -NYLQLLGLRSQTGEP--QTAW-----------------FQNVTMVMQKYAQHFNGSVPL
BrPCLS4         -SILNLFGFENS------QTNW-----------------FQY---XMHNYSKYFNGSVPL
BnPCLS4         -SVLNLFGFEXS------QTNW-----------------FQHTCMVMHNYSKYFNGSVPL
BoPCLS4         -SILNLFGFENS------QTNW-----------------FQYTCIVMHNYSKYDYGSVPL
RSCV1-dsRNA3    -NWTAFLGF------S-ERSDW-----------------FGSINAMMVKYCQFWKGSTTL
BrPCLS5_Cc      -NWLTFLRL------S-EDATW-----------------FGSLAAMMAKYCQFWKGSVPL
BrPCLS5_tu      -NWLTFLRL------S-EDATW-----------------FGSLAAMMAKYCQFWKGSVPL
BnPCLS5         -NWSNFLRL------S-GHSTW-----------------FGKVAAMMGKYCQFWKGSVPL
BoPCLS5         -NWSTFLRL------S-EHSTW-----------------FGSVATMMGKYCPFWKGYVPL
SpPCLS5         -DWISALRM------N-NSSSW-----------------LGPLAAMMAKYCQFWKGSCSL
StPCLS5         -DWISALRM------N-NSSSW-----------------LGPLAAMMAKYCQFWKGSCSL
SlPCLS5         -DWISALRM------N-SSSFW-----------------LGPLAAMMSKYCQFWKGSCSL
NtPCLS5-1       -SWISALRL------K-EDSTW-----------------FGPLAAMMSKYCQFWKGSCSL
RSCV1-CP        -TWTSFLCL------D-NDDSW-----------------FSPLAAMMNKYCQYWHGSAPL
HetRV3-ecl-CP   -SWTQFLGL------D--NMPF-----------------FLQVVRIMSWYSKFWQGSSDL
RSCV3-CP        AFVLGTACTSLIGMRD--RDDWQFYPNANVPAH------FNPHTAQRITPRTMY-GSYEV
MdPCL7          AYILGAACTPNLGFRD--VDDWQNGIVPEN---------VNPLSYDRVKYXHFY-GGYEV
BCV2ds1-CP      PYILGIPCTPRLGPRD--EDHYQHWTHNDPPLSTDGNIILNPLAYVRSSERKFF-GNAEY
BCV2ds2-CP      PHILATPCTAKLGIYD--ADIWQNCPGNIPITA------TTARTARRESERRFY-GSAET
FCCVds2-CP      ------------------GTAWWLLRQHFEDGIFELQLPLPEV--------NFTSSMALT
RoCVds2-CP      ------------------GTAWWLLRQHYEDHVFELQVPLPEV--------NFTSAMAIT
RSCVds2-CP      ------------------GSSWWLFKPDVTDGLLSIRCPLPED--------NYTLAGATV
MePCLS2         ------------------ASTWWLYRPVLTGLHFCLECPLPEE--------NFTLNTAVL
FCCVds3-CP      ------------------GTAWWSYKIHNDHGTSDLVCLLPPT--------NYSPLAVAT
RoCVds3-CP      ------------------GSAWWTYKLNNAHGTTDLVCTIPPT--------NYSDLGVAL
SbPCL8          ------------------GMPWWTYRVSTVYSTHDLICALPPT--------LCTYFSAEL
RSCVds3-CP      ------------------GNAWWTYRTSYDGDVYDLKINFPPL--------HFNDHLANL
                                     :

RnPV2W57-CP     ADC-------------------SPV-GPPVCQ---------------VQTTISNVDLH--
FestucaEST      ADC-------------------PPT-GIVSNQ---------------ICVEYIAPRTDVT
MgPCLS1         ADC-------------------SPSGGIVSNQ---------------IVVAYQGTSTPPI
AtPCLS1         ADC-------------------SPH-GLVANQ---------------VMVVLSTPEHLPE
AtPCLS1_No      ADC-------------------SPH-GLVANQ---------------VMVVLSTPEHLPE
AsPCLS1         ADC-------------------SPH-GLVANQ---------------VMVVLSTPEHLPE
AtPCLS1_C24     ADC-------------------SPH-GLVANQ---------------VMVVLSTPEHLPE
AaPCLS1-A       ADC-------------------SPH-GLVSNQ---------------VMVCLSTPEQVPG
AaPCLS1-B       ADC-------------------SPH-GLVANH---------------VMVCLSTPEQVPG
CrPCLS1         ADC-------------------PPH-GLVANQ---------------VMVCLSKPKNLPD
CbPCLS1         ADC-------------------PPH-GMVANQ---------------VMVCLSKPKNLPD
OkPCLS1-A       ADC-------------------SPH-GPVANQ---------------VMVCLSNPEHLPD
OpPCLS1-A       ADC-------------------SPH-GPVANQ---------------VMVCLSNPEHLPD
OkPCLS1-C       ADC-------------------LPH-GLVANQ---------------VMVCLSNPEHLPD
CPCV-CP         YEC-------------------PPV-GPPAAQ---------------VSMVRTQPMRFEA
FvBV-CP         QEC-------------------SPV-GSAPGQ---------------INGTLTDAIPVPT
SsPV-S-CP       ANI-------------------NPS-TGPSAV---------------VETTVA-------
CCRS-PV-CP      GSV-------------------TTT-GTGISY---------------IRTKYVDTKQNKN
ACD-PV-CP       GSV-------------------TTT-GTGISY---------------VRTKYVATRQNKN
WCCV-CP         KSI-------------------SPV-GIGASV---------------IYGTPLEDTNVRD
CCV-CP          KSI-------------------LPT-GIGAVV---------------IYGTPMNNPSTRT
BCV-CP          KSI-------------------STI-GIGAVA---------------VIGAPTPDPATRD
VCV-CP          KSI-------------------DLT-GIGAVA---------------LTGTPVNNTAVRD
BrPCLS4         SSI-------------------LPT-GLGAVA---------------VRGVPSVNEATRS
BnPCLS4         SSI-------------------LPT-GLGAVA---------------VRGVPSVNEATRS
BoPCLS4         SSI-------------------LPT-ELGAVA---------------VRGAPSINEATRS
RSCV1-dsRNA3    YEC-------------------SPA-SSAAGS---------------VRCIAT-DTNIFT
BrPCLS5_Cc      YDC-------------------SAS-DSAAGA---------------VRCTAT-DTDVFD
BrPCLS5_tu      YDC-------------------SAS-DSAAGA---------------VRCTAT-DTDVFD
BnPCLS5         YDC-------------------SAS-SSAAGA---------------VRCTAT-DTDVFD
BoPCLS5         YDC-------------------SAS-SSGAGV---------------VRCTAT-DTDVFD
SpPCLS5         ADC-------------------SPD-GSATGS---------------VRCVATGGTNVFQ
StPCLS5         ADC-------------------SPD-GSATGS---------------VRCVATGGTNVFQ
SlPCLS5         ADC-------------------SPD-CSATGS---------------VRCVSTGGTNVFQ
NtPCLS5-1       AEC-------------------SPN-SSAAGS---------------VRCLLSGHSNLFQ
RSCV1-CP        SSI-------------------PAD-GSAAGS---------------MICNELNGSSIYR
HetRV3-ecl-CP   MSF-------------------TPS-GHTGGQ---------------NTFELVNPPAS--
```

```
RSCV3-CP          RSY------------ETDHDYYIPSNSAGVTA---------------FLSGPSTSRKRSR
MdPCL7            RTIERRQIVVKLDEETLDRVLEDXRFYSGYAVGDGKQRXIPIFVRGXTQGPPPNKRKKTK
BCV2ds1-CP        RTMEYNSYTQNFEAY-------MPRVTTSTQA--------------------KNKRKKDA
BCV2ds2-CP        RTIEQRNYTVNFPELL------KPR-DTGARA----------------------------
FCCVds2-CP        LSLFLNA---------------EEV-NATSEI---------------FDLTPVGADI---
RoCVds2-CP        HTLFLEG---------------EAA-DPSNAV---------------FDLTPVGADT---
RSCVds2-CP        FMLFYHD---------------VGS-DPAIDL---------------FNIDSLGNDD---
MePCLS2           RTLFCND---------------ASG-GFMVNL---------------FDLTPVGTDT---
FCCVds3-CP        RMLFLAT---------------QDD-SDECTD---------------FIEWPEDLGD---
RoCVds3-CP        RSLFLAT---------------AAD-SDECSE---------------IIAWPEGTAD---
SbPCL8            RSLLLIP---------------DPDHNNEVQE---------------IVTHPQTVAHHGF
RSCVds3-CP        AIMFLGN---------------SGA-DRNNAS---------------IITTKTDDQD---


RnPV2W57-CP       ------TAPTSISKGKSFY-----------------AEAHMKLTTTSRT-MDQLSMIMAS
FestucaEST        -------APTHNADPLSLF------------------PFTIKLHSTARN-LPALSEVMAC
MgPCLS1           -------APTHSFDTRALF------------------PFSIRLHTTVRS-IPELAEAMAA
AtPCLS1           -------SPNCIADKRATY------------------EFGYQLKSTVRN-LPPLAEALAA
AtPCLS1_No        -------SPNCIADKRATY------------------EFGYQLKSTVRN-LPPLAEALAA
AsPCLS1           -------SPNCIADKRATY------------------EFGYQLKSTVRN-LPPLAEALAA
AtPCLS1_C24       -------SPNCIADKRATY------------------EFGYQLKSTVRN-LPPLAEALAA
AaPCLS1-A         -------SPTCIADKRATY------------------EFSYQLKSTARN-LPPLAEAVAA
AaPCLS1-B         -------SPTCIADKRATY------------------EFSYQLKSTARN-LPPLAEAVAA
CrPCLS1           -------PPTCIADKRATY------------------EFSYKLKSTAFN-LSPLTEAVAA
CbPCLS1           -------PPTCMADKRATY------------------EFSYKLKSTAFN-LPPLTEAVAA
OkPCLS1-A         -------APTCIADKRATY------------------EFSFKLKSTARN-LPPLAQAVAA
OpPCLS1-A         -------APTCIADKRATY------------------EFSFKLKSTARN-LPPLAQAVAA
OkPCLS1-C         -------PPTCIADKRATY------------------EFSYKLKSTALN-LPPLAETVAA
CPCV-CP           SNTAQAMETESFESTAISV-------------------------NRSLSLHDEKVALAAL
FvBV-CP           ISGTTVYTPSTLA------------------ALVTPWSSTTIYTHVNLS-LPEFFEHQAA
SsPV-S-CP         --VPAAAIPQATAWYEGYP-----------------FNLNLTVNTRVRS-ISETDVKIGT
CCRS-PV-CP        ILVHAVTTRKVRYQTGSTL----------RYHIPEFTGITTIHLHAEEF-LDLVTEQAGI
ACD-PV-CP         ILVHAVTTRKVRYQTGSTL----------RYDIPEFTGITTIHLHSEEF-LDLVTEQAGI
WCCV-CP           WLYPAAAAIAPFRSTRFLP------------RRELPATLAVRFAHADHE-IEEQAEQYSI
CCV-CP            WVYPPDTGIEPFTTTRYPP------------RREIPDALSIQFSHADHE-LEEQAEQYAM
BCV-CP            WFYPAATGIEPFLCSRFAP------------RREIPNTLGMIFSHADHE-LEEQAEQYAI
VCV-CP            WLYPPNANIEPFTTGRFNP------------RREIPAQLRIRFSHSDHE-LELQAEQYAI
BrPCLS4           FLYPTNAEIEPFTSSRFNP------------RRLIPLAMSVTFQHCEYEGLDEEAERYAI
BnPCLS4           FLYPTNAEIEPFTSSRFNP------------RRLIPLAMSVTFQHCEYEGLDEEAERYAI
BoPCLS4           FLYPTDAEIEPFTSSRFNP------------RRQIPLAMSVTFKHCENDGLGEEVERYAI
RSCV1-dsRNA3      PPEWNAQQGAHTSFTHGDA--NQAGHYNMRHN----AHLTFQATTSVHE-LPLAHYYSAL
BrPCLS5_Cc        PPHWIAQAGNRTATQHANP--HQVGHYSMRSN----LSLVFKAATSVED-ISRAHIFAAN
BrPCLS5_tu        PPHWIAQAGNRTATQHANP--HQVGHYSMRSN----LSLVFKAATSVED-ISRAHIFAAN
BnPCLS5           PPHWTAQAGNHNATQHANA--HQVGHYSTRSY----LSLVFKAATSVED-ISRAHISAAN
BoPCLS5           PPHWTTQARNHNSTQHANP--HQVGHYSMRSN----LSLVFKAATSVED-ISMANIFTAN
SpPCLS5           GPVWTNXTGSHNNFQHGNA--NQVGHSXMRSN----LHLMFDASTTIHD-IPDAHVYPAL
StPCLS5           GPVWTNXTGSHNNFQHGNA--NQVGHSXMRSN----LHLMFDASTTIHD-IPDAHVYPAL
SlPCLS5           GPVWTNEAGMHNNFQHGNA--NQVGHYTMRSN----LHLIFEASTTIHD-IPYSHVYPAL
NtPCLS5-1         GPVWSAQTGAHTNTQCDHA--DEAGHYCLRST----ANTRFTATTSIVD-LPNAHYYAAS
RSCV1-CP          LAQWTAPIMDPPADEDAPPVIAEPGYYYPNPN----PVLIFDARTCIED-ISSAHMFSAM
HetRV3-ecl-CP     -------LNAMTAGGRFYT-----------------LDLKSKGYSRNPL-APDADCFDAA
RSCV3-CP          TTSSSTPGSLVICK----------------------------------------------
MdPCL7            DKVSVSNAAIQTGSEYSEAITIQTGTESSKATAIQTGSESFKAEASTTV-DQFQIVDWTY
BCV2ds1-CP        APEQLSTGQGDGSSSSKDA--------EMSTEESEAPPPPKIPRPRQI--HQFRLIDWVY
BCV2ds2-CP        ---LVTTTQRASTSGSVEP-------------TTGAQPEPETPTFQITF-RQFRILDYCY
FCCVds2-CP        ------------------------------------------------------------
RoCVds2-CP        ------------------------------------------------------------
RSCVds2-CP        ------------------------------------------------------------
MePCLS2           ------------------------------------------------------------
FCCVds3-CP        ------------------------------------------------------------
RoCVds3-CP        ------------------------------------------------------------
SbPCL8            RMKALHTXNRHQNVSCTLA-----------------------------------------
RSCVds3-CP        ------------------------------------------------------------

                                                  GpfW
RnPV2W57-CP       ASHL-NLRYAHATH----------------SIQSRTSGPFW--SLNPIESSVTDESSLIQ
FestucaEST        TAQT-HIRM-FPTH-----PYFGQFGNHRG------CGPFW--EIRPIESSPKDESSYLS
MgPCLS1           FAQT-NISM-FATH-----PWLHAIDENSRD------GPFW--QIRPVEKSSIDQTSYLS
AtPCLS1           FSQT-YIRM-FPNH-----PFFGTFGSKTLD-----HGPFW--KIRPIGSSLTDNSSYLT
AtPCLS1_No        FSQT-YIRM-FPNH-----PFFGTFGSKTLD-----HGPFW--KIRPIGSSLTDNSSYLT
AsPCLS1           FSQT-YIRM-FPNH-----PFFGTFGSKTLD-----HGPFW--KIRPIGSSLTDNSSYLT
AtPCLS1_C24       FSQT-YIRM-FPNH-----PFFGTFGSKTLD-----HGPFW--KIRPIGSSLTDNSSYLT
AaPCLS1-A         FSQT-HIRM-FPNH-----PFFGTFGSKTLD-----HGPFW--KIRPIGSGLPDNSSYLT
AaPCLS1-B         FSQT-HIRM-FPNH-----PFFGTFGSKTLD-----HGPFW--KIRPIGSGLPDNSSYLT
CrPCLS1           FSQT-HVRM-FPNH-----PFFGTFGSKTLD-----HGPFW--NRRPIGSSLTDDSSNLT
CbPCLS1           FSQT-HVRM-FPNH-----PFFGTFGSKTLD-----HGPFW--NRRPIGSGLTDDSSILT
OkPCLS1-A         FSQT-HIRM-FPNH-----PFVGTFGSKTLD-----HGPFW--SRRPIESSLTDNSSYLT
OpPCLS1-A         FSQT-HIRM-FPNH-----PFVGTFGSKTLD-----HGPFW--SRRPIESSLTDNSSYLT
OkPCLS1-C         FSQT-HIRM-FPNH-----PFFGTFGSKT--------------------SLTDNSSYLT
CPCV-CP           INET------------------LVSTGDQPIYAAQRLGPFW--NISPTRRQAYRFDPSFV
FvBV-CP           LTNV-NVTE-------------HGAAPTFGTTATTLNGSFW--RMKPHYRESPPINVTKN
SsPV-S-CP         FAATCNII--------------TGGNFTRWNGSSVFAGDFFVAATNPINFENPTEVNAIA
CCRS-PV-CP        LTQL-NSDWTDINK--------DVTDTSGPDYTSTHVGPIF---SIPDSGRTSQYKVSNT
```

```
ACD-PV-CP       LTQL-NSDWTDINK--------DVTDTSNPDFKSTHVGPIF---SIPDSRRTSQYNVSNT
WCCV-CP         LCHT-NMKWYVNNA--------TQNNHTAIEGNYIHQGEYW--NFTPFRYSPP-VSLKTQ
CCV-CP          LTHT-NIKWYQNLE--------TQNNWTAIAAGGLYLGDYW--NMMPYRFSAP-LHYKSQ
BCV-CP          LTHT-NIRWSPSVV--------AQNAWTAVNDGASRNGDYW--IMMNYRFSTR-ISLKTQ
VCV-CP          AAHT-NIRWAANIA--------TQHERTAINPAHLHQGDYW--NMTPFRHTGH-LGLKTQ
BrPCLS4         VAHT-NLRWPLENG--------DQNEWTLVNSCVTHRGDVW--SFMCHRFSNPVVSLHFQ
BnPCLS4         VAHT-NLRWPLENG--------DQNEWTLVNSCVTHRGDVW--SFMCHRFSNPVVSLHFQ
BoPCLS4         VAHT-NLRWPLENG--------DQNEWTRVNSCVTHRGDVW--SLMCHRFSNPVVSLHLQ
RSCV1-dsRNA3    TYNI-NL---------------AESEAAM--AATC-RGTFW--NIFPDAYTRSGIQIYPG
BrPCLS5_Cc      TYNI-YL---------------SPNDDGH--GALR-RGVFW--SIHPDAFTKSGIEIYPG
BrPCLS5_tu      TYNI-YL---------------SPNDDGH--GALR-RGVFW--SIHPDAFTKSGIEIYPG
BnPCLS5         TYNI-YL---------------SPNDDGHAGRAQYWRGMFW--SIYPDAYTRSGIKT-PR
BoPCLS5         TYNI-YL---------------SPNDDGH--RALH-RGMFW--NIYPDAYTRSGIETYPD
SpPCLS5         TYHF-NL---------------SPNDAGR--TVLN-EGPFW--DVHPPAFQNSGIQVYQG
StPCLS5         TYHF-NL---------------SPNDAGR--TVLN-EGPFW--DVHPPAFQNSGIQVYQG
SlPCLS5         TYRF-NL---------------SPSVAGL--TVLN-EGPFW--DVHPPALQKSGVQVYQG
NtPCLS5-1       TYQF-NL---------------AATAAEQ--ANLL-QGPFW--TVVPDAWTKMDISVFQG
RSCV1-CP        TFHP-NL---------------IPYGANR--ANFL-KGQFW--NCHPPSYSAPAHQVYPA
HetRV3-ecl-CP   LGPI-NLSWTPADMFAGKLARNGAIADETTLSNTRTAGPYF--SETIVDITGE-INPSSA
RSCV3-CP        -----------------------------------------YICLYPYRLHYLPVHINIS
MdPCL7          SHRV-ILSM----------------------DSDTRSQSLW-------------------
BCV2ds1-CP      HSRVILGA-----------------------DKAMQRRALW-------------------
BCV2ds2-CP      LAKVIHKS-----------------------NSQMINKALR-------------------
FCCVds2-CP      -----------------------------------------YGYIMREPHLGINVSTFEV
RoCVds2-CP      -----------------------------------------YGYILREPHFGINVTTYQV
RSCVds2-CP      -----------------------------------------YGSFIRNPRDGFNASSYYA
MePCLS2         -----------------------------------------YGSMLRNPQYEVDVTTYYA
FCCVds3-CP      -----------------------------------------YGTQLKEAPPNSNVRAFLA
RoCVds3-CP      -----------------------------------------FGTNLRENPPNSNIRAFLA
SbPCL8          ------------------------------------TSLMYFGSSLRDSIEAENLKAPVS
RSCVds3-CP      -----------------------------------------YGWYVKDIREGEQARAFAA


RnPV2W57-CP     V--RQTVKKMMKSKV---------
FestucaEST      Y--KGVVRKLLKPK----------
MgPCLS1         L--KDIIRGLIRHKSTLIL-----
AtPCLS1         I--PSIVKQAFK--GPAT------
AtPCLS1_No      I--PSIVKQAFK--GPAT------
AsPCLS1         I--PSIVKQAFK--GPAT------
AtPCLS1_C24     I--PSIVKQAFK--GPAT------
AaPCLS1-A       I--PSIVKQAFKANGPAT------
AaPCLS1-B       I--PSIVKQAFKANGPAT------
CrPCLS1         I--PSIVKQAFKSKVPAT------
CbPCLS1         I--PSIVKQAFKSKVPAT------
OkPCLS1-A       I--PSMVEQAFKSKGPAA------
OpPCLS1-A       I--PSMVEQAFKSKGPAA------
OkPCLS1-C       I--PSMVKQAFKSKGTAT------
CPCV-CP         L--PSVIRDHYALTTATR------
FvBV-CP         L--FNIISNEMFAEPTS-------
SsPV-S-CP       MAQPTMTLSLYRPKGGEANSDD--
CCRS-PV-CP      I--APLISGYYHTPSALRFE----
ACD-PV-CP       I--APLISGYYHTPSALRFE----
WCCV-CP         F--AQVIASRYHQQAANRAE----
CCV-CP          Y--AQIIASRYHQQAANKAE----
BCV-CP          F--AQVIASRYHQQAANRVD----
VCV-CP          Y--AQLIASRYHQLAANRVD----
BrPCLS4         L--GQVIVSRYHLHELYLDD----
BnPCLS4         L--GQVIVSRYHLHELYLDD----
BoPCLS4         L--GQVLVSRYHLHELYLIY----
RSCV1-dsRNA3    I--PALIARDYHSETRIESERQNA
BrPCLS5_Cc      V--FSSLARDYHSHSPIYPD----
BrPCLS5_tu      V--FSSLARDYHSHSPIYPD----
BnPCLS5         V-----------------------
BoPCLS5         V--IPSLARDYHSYSRIDSDGL-S
SpPCLS5         V--TLTIARDYHSDTRIDSEHQ--
StPCLS5         V--TLTIARDYHSDTRIDSEHQ--
SlPCLS5         V--TLTIARDYYSGTRIDSEHQ--
NtPCLS5-1       V--LTSIARDYHSDVRIDAERE--
RSCV1-CP        V--SALIAREYHSETNLSSDK---
HetRV3-ecl-CP   Y--AGILNQYYYSSVALKN-----
RSCV3-CP        LFN---------------------
MdPCL7          ----------------EIVQNLDC
BCV2ds1-CP      ---------DFIHAGATPSHQPRP
BCV2ds2-CP      ---------NFIQKGASKKTE---
FCCVds2-CP      IDESAKE--IVSNV----------
RoCVds2-CP      IDGSAQE--IVSNV----------
RSCVds2-CP      ISSEGTDEMWKSSA----------
MePCLS2         IEDAGVNLYSLG------------
FCCVds3-CP      LCHGPLEEWSNGHA----------
RoCVds3-CP      LCHGPLEEWSNGHA----------
SbPCL8          L-----------------------
RSCVds3-CP      LCQWDPRYWNEKNNV---------
```
