# Supplementary material for: Widespread Endogenization of Genome Sequences of Non-Retroviral RNA Viruses into Plant Genomes
Source: PLoS Pathog. 2011 Jul 14;7(7):e1002146. doi: 10.1371/journal.ppat.1002146 (PMC3136472; doi:10.1371/journal.ppat.1002146)
Supplement: Figure S5 — Alignment of plant rhabdovirus and varicosavirus N proteins and plant nuclear encoded RNLSs. The entire nucleocapsid protein (N) sequences (approximately 450 aa) of plant rhabdoviruses and varicosaviruses (approximately 450 aa) and plant rhabdovirus N-like proteins (RNLSs) were aligned using the program MAFFT version 6. The alignment was used to generate a phylogenetic tree, as shown in Figure 6. For non-abbreviated virus names and information on RNLSs, see the Figure 6 legend, and Tables 2 and S5. Two conserved motifs GmH and YaRifdxxxfxxLQtkxC are marked in red. (PDF) [file ppat.1002146.s005.pdf]

```
CLUSTAL format alignment by MAFFT (v6.847b)


OFV_N           MANPSEIDYM--------------------------------------------------
PYDV-N          MNNANTAQFVQLLKGG--------------------------------------------
SYNV_N          MSTTPTITLADLERIREP------------------------------------------
LNYV_N          M-TTSAEKLAKLEQLRKERAAVIQKPTTQVSSEPVVTEKPR-------------------
LyMoV_N         MAETMAEKLARLQALRGGKSEV--KSTPQPQEQPKIAR----------------------
Triphysaria_EST MEL---------------------------------------------------------
PtRNLS3         MEAAAAKGKGIQINKPQAQASQPMARSAEAEM----------------------------
NtRNLS2         MAANTIDLNTM-------------------------------------------------
NCMV_N          MANEHKSPLDKR------------------------------------------------
LBVaV_Cp        MAHPKL------------------------------------------------------
TStV_CP         MAHPKL------------------------------------------------------
MdRNLS1_1       M-----------------------------------------------------------
MdRNLS1_1FUJI   M-----------------------------------------------------------
Festuca_EST     MKTQRMKKRTDEA------------------------------------PKMPRVSAKVA
MgRNLS2         MADNMSALRKAMDKLILHIEKHEAEQSSSSTSGQRLVIPDKWKALFESIDGPT-------
Boleracea_EST   MDALSDLRRATALLEKRKRVRDDVEDDVEEVQ----------DASDAEEEVIPGPEMNVV
LjRNLS1_2       MF----------------------------------------------------------
AqcRNLS1        MGTYQY------------------------------------------------------
Aquilegia_EST   MGTYQY------------------------------------------------------
AqfRNLS1        MGTYHY------------------------------------------------------
LjRNLS1_1       MNVEDLLREIAKARIRNAENVDPPKDAA--------------GIIIPENVMPLQPTEQTS
LjRNLS1_1B129   MNVEDLLREIAKARIRNAENVDPPKDAA--------------GIIIPENVMPLQPTEQTS
Cichirum_EST    M-----------------------------------------------------------
BrRNLS1         MATSPN------------------------------------------------------
BrRNLS1-1A      MATSPN------------------------------------------------------
BrRNLS1-1B      MATSPN------------------------------------------------------
BnRNLS1-1       MATSPN------------------------------------------------------
BoRNLS1-1_Ca    MATSPN------------------------------------------------------
Bnapus_EST      MATSPN------------------------------------------------------
BnRNLS1-2       MATSPN------------------------------------------------------
RsRNLS1-1A      MATSPN------------------------------------------------------
RsRNLS1-1B      MATSPN------------------------------------------------------
Picea_EST       XPRPSSIIGSPPSSWHCRGWNVSPYDTSPQTHQGSQFYQINWKTNILYFLLSSDLFDKDL
CsRNLS1         MANVNSGSSTADDFARRIEASLRAAISQHEAAIQSLPAPVRQEVETAATEQAENSVGAIV


OFV_N           ----------------TPLSA---------------------------------------
PYDV-N          ----------------KEYED---------------------------------------
SYNV_N          ----------------YKVLS---------------------------------------
LNYV_N          ----------------VRNTA---------------------------------------
LyMoV_N         ----------------VANKL---------------------------------------
Triphysaria_EST ----------------KMISK---------------------------------------
PtRNLS3         ----------------ELNRR---------------------------------------
NtRNLS2         ----------------NVPAA---------------------------------------
NCMV_N          ----------------VTDLF---------------------------------------
LBVaV_Cp        ----------------KMLDA---------------------------------------
TStV_CP         ----------------KMLDA---------------------------------------
MdRNLS1_1       ----------------DLREM---------------------------------------
MdRNLS1_1FUJI   ----------------DLRGM---------------------------------------
Festuca_EST     PKKKRRKMRHLRVMDEMKLSA---------------------------------------
MgRNLS2         ----------------DRTRY---------------------------------------
Boleracea_EST   ETTDLESRKRAFLAKPIRRAV---------------------------------------
LjRNLS1_2       ------------------------------------------------------------
AqcRNLS1        ----------------RVNEK---------------------------------------
Aquilegia_EST   ----------------RVNEK---------------------------------------
AqfRNLS1        ----------------RVNEL---------------------------------------
LjRNLS1_1       IEARLEKYSSTTFTIPPISER---------------------------------------
LjRNLS1_1B129   IEARLEKYSSTTFTIPPISER---------------------------------------
Cichirum_EST    ------------------------------------------------------------
BrRNLS1         -EPDD-TLSASSFFNPHLMKK---------------------------------------
BrRNLS1-1A      -EPDD-TLSASSFFNPHLMKK---------------------------------------
BrRNLS1-1B      -EPDD-TLSASSFFNPHLMKK---------------------------------------
BnRNLS1-1       -EPDD-TLSASSFFNPHLMKK---------------------------------------
BoRNLS1-1_Ca    -EPDD-TLSASSFFNPHLMKK---------------------------------------
Bnapus_EST      -EPDDGTVSASSFFNPHLMKK---------------------------------------
BnRNLS1-2       -EPDDGTVSASSVFNPHLMKK---------------------------------------
RsRNLS1-1A      -EPDD-TVSVSRMPKLKNLKR---------------------------------------
RsRNLS1-1B      -EPDD-TVSASRIPKLKNLKR---------------------------------------
Picea_EST       SPEFRDLFDKDDIKVDHEFRD---------------------------------------
CsRNLS1         RLRNEERGKGTAADTSVKPQASQKAPQVAPAVINPVPAPIAAQPATVGNIIPRIVGPLEA


OFV_N           ------------YEGVPAEYQEATSSPTPKEYTRDAAKA--IPICILP---APPGNEVEV
PYDV-N          ------------WNSRDDIPAA-DGKLVQIEYSDADFWSKLTTIYSL----DDLGPS-DL
SYNV_N          ------------KTARPENPSG---QCTYREYLFSDAVK--YPIYKR----ATMTNE-EI
LNYV_N          ------------YDSLAGVTIG---KRASRKWSDADIKS--IPIYDV----HQVPAA-QV
LyMoV_N         ------------YDAVDDVTVG---KRASKRWDDNDLAN--VVCYDV----AQLTAG-NM
Triphysaria_EST ------------YADLDDSVL----FNAGAKWSDDEIKN--IKVHKI----PRLTIN-QI
PtRNLS3         ------------YGHLAGVVIA-SGNLLPKTWSDSELSK--INTYNV----KQLDSA-QS
NtRNLS2         ------------YAAAANLIPL-TQGLPEENWSDDKKGA--IPIWHV----IPLDDN-NT
NCMV_N          ------------KDIPTDISTH---LITGETFSNESFQA--IPVYTL----TNVTVK-QA
LBVaV_Cp        ------------FSDVVEITGK---TAGKESWDDESTIA--MPSYKL----SVLSDA-DA
TStV_CP         ------------FSDVVEITGK---TAGKESWDDESTIA--MPSYKL----SVLSDA-DA
MdRNLS1_1       ------------LKGLEHITF---VVETKTDWVDAKCLA--GDGIHL----KVLENE-VL
MdRNLS1_1FUJI   ------------LKGLEHITFR--VVETKTDWVDAKCLA--GDGIHL----KVLENE-VL
Festuca_EST     ------------DDNRKMRATF---GKTTTRWTDNDMMR--RPTFTL----KVVRGE-KL
MgRNLS2         ------------LQLMENSLDG---TTSMNDWKDLQPGQ--KPVIKL----HRLTVD-QI
Boleracea_EST   ------------NQNYYESKTP---MPCKRVWRDKEPGQ--MAVVNL----RRLTEE-EI
LjRNLS1_2       ----------------------------------------------------------EL
AqcRNLS1        ------------YSGLDSL------FDGRMDWDDTMLCG--KKGVRV----YTLTNN-EM
Aquilegia_EST   ------------YSGLDSL------FDGRMDWDDTMLCG--KKGVRV----YTLTNN-EM
AqfRNLS1        ------------YSDLDSL------FDGRVDWDDTMLCG--KKGVRV----YTLTNN-EM
LjRNLS1_1       ------------WLNLPDLF-----TPTTHEFSDTKLLN--ESGYVI----RKTTTE-NL
LjRNLS1_1B129   ------------WLNLPDLF-----TPTTHEFSDTKLLN--ESGYVI----RKTTTE-NL
Cichirum_EST    ----------------------------KYDFVDREWIG--KSGIML----ERFTDE-KI
BrRNLS1         ------------WEALSSVRHK--PNPNKVVWTNEMLKD--RKVINLDAWNQVGTFD-ER
BrRNLS1-1A      ------------WEALSSVRHK--PNPNKVVWTNEMLKD--RKVINLDAWNQVGTFD-ER
BrRNLS1-1B      ------------WEALSSVRHK--PNPNKVVWTNEMLKD--RKVINLDAWNQVGTFD-ER
```

```
BnRNLS1-1        ------------WEALSSVRHK--PNPNKVVWTNEMLKD--RKVINLDAWNQVGTFD-ER
BoRNLS1-1_Ca     ------------WEALSSVRHK--PNPNKVVWTNEMLKD--RKVINLDAWNQVGTFD-ER
Bnapus_EST       ------------WEALSSVRHK--LNPNKVVWTNEMLKD--RKVINLDAWNQVGTFD-ER
BnRNLS1-2        ------------WEALSSVRHK--LNPNKVVWTNEMLKD--RKVINLDAWNHVRTFE-ER
RsRNLS1-1A       ------------YEDVSFI-NE--LNVNKVVWSNEMLKD--SKVINLDAWNQVGTFE-ER
RsRNLS1-1B       ------------YEDVSFI-NE--LNVNKVVWSNEMLKD--RKVINLDVWNQVGTFE-ER
Picea_EST        ------------LKSFEIIRTG---PTPTLDWSDDKFLQ--YSMYNI----KQLSND-EI
CsRNLS1          DVIGIRGMQLVHREELPDVGYLHPGFIRELEWSDGILRE---MGGRL----SILPDA-DL


OFV_N            AEAFREA-----TQGTET-VLSKLQLAQIMSL-----GFMIQMSGDPEALM---------
PYDV-N           VAAWTRI-----RESIDNSTISESIIGEIFRV-----AAHIKKVEDKTSRIIGSF-----
SYNV_N           VTFFGKI-----TSDKHT-HMTESDMWTFVQC-----ALSLKDPVDRSSIFDKGFWDANH
LNYV_N           IALGKDL-----LTQIQNNSVNTVTVDYCLVL-----AVSIPKPAMTSFEHLLTP-----
LyMoV_N          ISIGKTL-----LSNIASGAITSKTIDACLAL-----AVSIPKPATSKFSAMLSP-----
Triphysaria_EST  LTKGQWF-----INQVNSNAIT-ATVANCLEL-----ALHIGCMGLTRWSPLLLP-----
PtRNLS3          MIVGSAL-----LMEIRNDKVSRDLLYKILLL-----AISLPLPGGDVDAHLLTP-----
NtRNLS2          VTVGAEF-----LQQISNNTISVKTAINCFKL-----AMSLKSPDTGVADPLLSQ-----
NCMV_N           VHLSYKL-----FESMKTTKCAAGYQYAIYLL-----AQRLLTPAAGSNDLVFKD-----
LBVaV_Cp         VREVKIF-----LTGLFV-RSSPRAIAAALIM-----TWNMRSVDPVAVRIFPAK-----
TStV_CP          VREAKIF-----LTGLFV-RSSPRAIAAALIM-----TWNMRSVDPVAVRIFPAK-----
MdRNLS1_1        LKMINEM-----LKGLTT-GMTERIAGYILIL-----VWNMRSASDNSRRIFPEK-----
MdRNLS1_1FUJI    LKMINEM-----LKGLTT-GMTKRIAGYILIL-----VWNMRSASDNSRRIFPEK-----
Festuca_EST      MKLCDAV-----IDGLTD-KVTEKVAKRIMQA-----AYNLHGLAKDS-RVFKNV-----
MgRNLS2          CELGVEV-----FRGLKT-GVSHKTVGGLMLL-----AFNLTDKFDLPLFADPKF-----
Boleracea_EST    NILGKEV-----FEAISS-EISDQTVAGLFLL-----AFNSKDRDGSNIYSDIPA-----
LjRNLS1_2        KNTGRSLYTLLHFEEMHT-CLTTQTVAKLLML-----SYNLRDVEGRELFGPTEP-----
AqcRNLS1         VELGSRV-----LRNVMQ-SVNEETVGAIILL-----AWNLRNPENLAQPIFPNH-----
Aquilegia_EST    VELGSRV-----LRNVMQ-SVNEETVGAIILL-----AWNLRNPENLAQPIFPNH-----
AqfRNLS1         VELGSRV-----LRNVMQ-SVNEETVGAIILL-----AWNLRNPENLAQPIFPNH-----
LjRNLS1_1        AGMGRIV-----IPMLCS-GFTRTGVVGLFTL-----AYNLKDSESANWEDAIDD-----
LjRNLS1_1B129    AGMGRIV-----IPMLCS-GFTRTGVVGLFTL-----AYNLKDSESANWEDAIDD-----
Cichirum_EST     AALGRVV-----IPMLSS-GFTGESCSDFCSR----------------------------
BrRNLS1          IQIIREA-----LTSLTI-EVSFRSVGALMVC-----AWGLVSCENPNESVFGGL-----
BrRNLS1-1A       IQIIREA-----LTSLTI-EVSFRSVGALMVC-----AWGLVSCENPNESVFGGL-----
BrRNLS1-1B       IQIIREA-----LTSLTT-EVSVRSVGALMVC-----AWGLVSCENPNESVFGGL-----
BnRNLS1-1        IQIIREA-----LTSLTT-EVSVRSVGALMVC-----AWGLVSCENPNESVFGGL-----
BoRNLS1-1_Ca     IQIIREA-----LTSLTT-EVSVRSVGALMVC-----AWGLVSCENPNESVFGGL-----
Bnapus_EST       IQIIREA-----LTSLTI-EVSFRSVGALMVC-----AWGLVSCENPNESVFGGL-----
BnRNLS1-2        IQIIREA-----LTSLTT-EVSVRSVGALMVC-----SWGLVSCENPNQSVFGGL-----
RsRNLS1-1A       TKIIREA-----LTCLTN-EVSIRSVGALMVC-----AWGLVSSENPNQSVFGGL-----
RsRNLS1-1B       TQIIREA-----LSCLTT-EVSIRSVGALMVC-----AWGLVSSENPNQSVFGGL-----
Picea_EST        KVTGVEL-----IKDLKE-HITWRTGYNILCL-----AFSIQSRIPGKKYAISDT-----
CsRNLS1          KEHAERL-----WRGLTV-QCTNRVTGIAMLYISLEPPFTIKSAGNRIXNAI--------


OFV_N            ---------PGVAEGSFL---------ETMTMPDIKTRQDSRITAAIAAMALDPTEEGPD
PYDV-N           -------APPTARGVVTK---------TGLDTAPIVFGQSVTVGADITVQNTAP------
SYNV_N           LCADYATAQPANTGKVAM---------SHHNPGVVVTQLVPKYDTGGSS-----------
LNYV_N           -------PSPEQGTRLDF---------TQPQAGVSNRSGLTAIEKMTLNATRKNLLT-ET
LyMoV_N          -------PPDGVGKKITF---------EQPSASSVARVGLTQLQQKQLVKSREAFAA-ET
Triphysaria_EST  -------LEKDVGFDLGW---------ESPKVTGTS-------HQLTKDLIQRERMT---
PtRNLS3          -------PPDGYGTQSME---------AKIDTENNQDDELAEAEAELLAMSKEREKK---
NtRNLS2          --------------------------ADRPAAATANPTVLAREEMPASDTFPNTPYTLQA
NCMV_N           -------YIPQGGSDLPQEIKNKIYVTTELSDSQTAGKSEAEKKKATADLIFGDNTDHDL
LBVaV_Cp         -------DKGKDTADVDV---------KNLEVPGVDYIDAMVET----------------
TStV_CP          -------DKGRDTADVDV---------KNLEVAGVDYIDAMVET----------------
MdRNLS1_1        ---------SPSNQGIDV---------EALSDGVVSPRGTTPIKLSAA------------
MdRNLS1_1FUJI    ---------SPSNQGIDV---------EALSHGVVSPRGTTPIKLSAA------------
Festuca_EST      --------NPCLPVPMEE---------GYLNSRIAFEGSIREYE----------------
MgRNLS2          -------ELVDKSKEMDI---------IELSATPINNLTTSSASMNFGE-----------
Boleracea_EST    -------DLIDEDNLVHQ------------DKMTLRIKKKVDGADMIKVK----------
LjRNLS1_2        -------ELIDKAQQRQL---------EHLDGIMLFCKELMVYAPSIVIYYGN-------
AqcRNLS1         -------SPCSSPEDVEL---------CTLSKRVLNDNPRVRGT----------------
Aquilegia_EST    -------SPCSSPEDVEL---------CTLSKRVLNDNPRVRGT----------------
AqfRNLS1         -------SPCSSPEDVEL---------CTLSKRVLNDNPRVRGT----------------
LjRNLS1_1        --------IPAESSFLDT---------ELDRDAEIHYKSSGQHKP---------------
LjRNLS1_1B129    --------IPAESSFLDT---------ELDRDAEIHYKSSGQHKP---------------
Cichirum_EST     --------LSSKAPGSRL---------GLLSSTQMKVYSDDRSTVQRPFGQDKGDKC---
BrRNLS1          -------WESSKARELEE--------YTRLSDGILCKSSRWRSPPDDKDLIL--------
BrRNLS1-1A       -------WESSKARELEE--------YTRLSDGILCKSSRWRSPPDDKDLIL--------
BrRNLS1-1B       -------WESSKARELEE--------YTRLSDGILCKSSRWRSPPDDKDLIL--------
BnRNLS1-1        -------WESSKARELEE--------YTRLSDGILCKSSRWRSPPDDKDLIL--------
BoRNLS1-1_Ca     -------WESSKARELEE--------YTRLSDGILCKSSRWRSPPDDKDLIL--------
Bnapus_EST       -------WESSKARELEE--------YTRLSDGILCKSSRWRSPPDDKDLIL--------
BnRNLS1-2        -------WESSKARELEE--------YTRLPDGILCKSSRWRSPPDNEDLIL--------
RsRNLS1-1A       -------WESSKARELEE--------YTTLPDGVRCTSSRWRSPPD--------------
RsRNLS1-1B       -------WESSKARELEE--------YTTLPDGVRCTSFRWRSPSD--------------
Picea_EST        -------GNFQDTQALPQ---------DARDQIFFSPSAPFTA-----------------
CsRNLS1          -------DYFTTPRDQHV---------NSLSEQILGVGNARNP-----------------


OFV_N            DLEDRSH---------------------QADAESAMSKARAMVYICLSLMRLAVKPAESF
PYDV-N           ----------------------------TVRTTDAGDAVVSAPYLCMALLRLMTKPVESF
SYNV_N           ----------------------------SQETESMASKAEAISFYFAWLTRFSVKQAPNT
LNYV_N           DEEKRARYEAIIKKMEDQEAGLGTS---KATVVTSETEAAAYGFLAATLLKLYAKSTESY
LyMoV_N          DEERKTALDKIIKGLEAQEAGTPST---SRAQVVDETEAAAYGFLAAIIIKLCAKTAESF
Triphysaria_EST  --------NMIESQFAQIKISTGEVSEEEIPRGMNEMDANAICYLAAYCLRLYGKTEQAW
PtRNLS3          KNKKKATSSIANVLLENLDVKGDDE---GEDNRKRDMSACAYCYLLPIFXRLAIKTPESF
NtRNLS2          PQVVPGPAPAVILPVANAAAAAHVAVVPQAGEDTQEEKRYAYCFIAAYLMKLLVKSADNV
NCMV_N           Q---------------------------QVRSQDDETIIRFGSFISAFLMKLIVKQQSNI
LBVaV_Cp         ----------------------------NVKDASDIEIIRAGAFIAASTLKMFAKSFTGW
TStV_CP          ----------------------------NVKDASDIEIIRAGAFIAASTLKMFAKSFTGW
MdRNLS1_1        ----------------------------GGNSYSLNECVRVGCFLAASLLKLFRKPAYSW
MdRNLS1_1FUJI    ----------------------------GGNSYSLNECVRVGCFLAASLLKLFRKPAYSW
Festuca_EST      ----------------------------APDDIDEEDYTRACCYIAASTLRLFTKSTDNF
MgRNLS2          ----------------------------RFTDYQKGRFAKSCCFIACSYLRLYSKPVDNY
Boleracea_EST    ----------------------------EELINETQTMRDCYCFLAGTYMRMFTKSAENY
LjRNLS1_2        ----------------------------LNTAPGRARIANALCFLAASFLRLFIKSSDNF
AqcRNLS1         ----------------------------LRFPTSVSEAAASVSYVCASLLRLFTKSVNNY
Aquilegia_EST    ----------------------------LRFPTSVSEAAASVSYVCASLLRLFTKSVNNY
```

```
AqfRNLS1        ----------------------------LRFPTSVSEAAASVSYVCASLLRLFTKSVNNY
LjRNLS1_1       ----------------------------FPATQDVRTEAAYYCYIAASVLRLFTKPPENY
LjRNLS1_1B129   ----------------------------FPATQDVRTEAAYYCYIAASVLRLFTKPPENY
Cichirum_EST    ----------------------------DRCNRDPTTEAAVYSYVAATLFRLFTKPASYY
BrRNLS1         ----------------------------FTTTGNGLNWVRTACFYCAAVLRLATKEHDAL
BrRNLS1-1A      ----------------------------FTTTGNGLNWVRTACFYCAAVLRLATKEHDAL
BrRNLS1-1B      ----------------------------FTTTGNGLNWVRTACFYCAAVLRLATKEHDAL
BnRNLS1-1       ----------------------------FTTTGNGLNWVRTACFYCAAVLRLATMEHDAL
BoRNLS1-1_Ca    ----------------------------FTTTGNGLNWVRTACFYCAAVLRLATMEHDAL
Bnapus_EST      ----------------------------FTTTSNGVNWVRTACFYCAAVLRLATKEHDAL
BnRNLS1-2       ----------------------------FTTTGNALNWVRTACFYCAAVLRLATKEHDAL
RsRNLS1-1A      ----------------------------SVTPGNALNWVRTACFYCAAVLRLATKEHDSL
RsRNLS1-1B      ----------------------------FVTAGNALNWVRTACFYCAAVLRLATKEHDSF
Picea_EST       ----------------------------KWDFGDDDVAVKAYCYVAFSLLRLLTKSHVAY
CsRNLS1         ----------------------------ELSDVPLADLIKSSCYIAASLLRLFTXKTGSW
                                                           :      ::

OFV_N           MKGVH--QIKQAYSVLVGEHSEFLFNYSYSEGMCRNIADMFNQC-DDLKATLCHHCAIAD
PYDV-N          NRSLT--TIRTSYGRFYGMQSAEVTNFSAPLNSLQQLSTGLDTY-PTCNSTMAWMMGFAE
SYNV_N          INVLYD-RVRATYLKFYSTSSSIFDTFRPSNTWLQGLKDAFDTF-PRVKNTLILHVAHAE
LNYV_N          VAGLA--QIRNRFAAWYDCPKAVLDAFQPTEAALVSLRAAFARR-PEVLSTWTLWVAVNE
LyMoV_N         IEGLP--RVRDRFSSWYDTSSQVIKTFNPTESVLNTLRTGFGRR-PEILSTWVLWVAYNE
Triphysaria_EST VGKLQ--LAKTRYRSWYKHDSSVMYLFSPSEYQAKKLRDAFNRK-PELGSTWVLTIAYNE
PtRNLS3         LRSIE--NFKARYQGWYDDGSDILVNLGLTDEFATSLREALGRD-PPLVNTWVMWVAYNE
NtRNLS2         VTGLT--AMRTRYEGFYGPARTV-ATFSLSQAQAIALKKGLASR-EKIMVTYTMALAHTQ
NCMV_N          VDGWN--GMLERYTNFYGESPIT-AVPKPNADWLAGLKNYLISD-PKIGHTWVRVISAAE
LBVaV_Cp        TQAWEHKHIQKRYADFCKTEYPF-KEFTTNNKCAETMYEAYQGQ-KLYQGTLGRILYALG
TStV_CP         TQAWEHKHIQKRYADFCKTEYPF-KEFTTNAKCAETMYEAYQGQ-KLYQGTFGRILYALG
MdRNLS1_1       DKAKY--HIQRAYTNYTKEPWPL-KDLNVIASHVGTLSLLWERK-TIFRDTLGSILYSIG
MdRNLS1_1FUJI   DKAKY--HIQRAYTNYTKEPWPL-KDLNVIASHVGALSLLWEGK-NIFRDTLGSILYSIG
Festuca_EST     MKALS--HIKAKYYRFYKDEFPL-KGFAVDKKCIEGLKEIYTNK-HVFKNALAPFLYSFS
MgRNLS2         LRIGH--LLRNSFAKFYGFQLAL-QNFHPEKEGIQAVKTVMDLQ-SVIKNTFYVLLYAGE
Boleracea_EST   VNIEN--KLIERFEHFYMTNFPF-EDFHPNLESARAIKSIFELS-TIMKNTFYTLLYAGE
LjRNLS1_2       SKIGQ--QLQNRFADFFSYAFPV-SRFMPPTTVIESIRDCFSRQCSIIKETFYVLLYSGE
AqcRNLS1        LRALP--YLNSSFEDFYHFKFPL-TWYNPSQESLEAISDKFRSN-SLFKYGMAGMIYLHN
Aquilegia_EST   LRALP--YLNSSFEDFYHFKFPL-TWYNPSQESLEAISDKFRSN-SLFKYGMAGMIYLHN
AqfRNLS1        LRALP--YLNSSFEDFYHFKFPL-NWYNPSQESLEAISDKFRSN-SLFKYGMAGMIYLHN
LjRNLS1_1       SKAWN--HIHTNFTRFYSRSPPV-IPNSPNIEVLRGLHSVFEMQ--KYKVTLFKLLYASS
LjRNLS1_1B129   SKAWN--HIHTNFTRFYSRSPPV-IPNSPNIEVLRGLHSVFEMQ--KYKVTLFKLLYASS
Cichirum_EST    VKTWS--HVLNGFSNSYGEPMKV-ILPVPTEAAVQGLKWWFLRE--REKVTLYRFLYMSN
BrRNLS1         VKAWS--YLPEHYQSFYKAPLEF--SLSLDHECLKCLRRLLQKS-TTIRNSVAPFLLAFQ
BrRNLS1-1A      VKAWS--YLPEHYQSFYKAPLEF--SLSLDHECLKCLRRLLQKS-TTIRNSVAPFLLAFQ
BrRNLS1-1B      VKAWS--YLPEHYQSFYKTPLEF--SLSLDHECLKCLRRLLQKS-TTIRNSVAPFLLAFQ
BnRNLS1-1       VKAWS--YLPEHYQSFYKAPLEF--SLSLDHECLKCLRRLLQKS-TTIRNSVAPFLLAFQ
BoRNLS1-1_Ca    VKAWS--YLPEHYQSFYKAPLEF--SLSLDHECLKCLRRLLQKS-TTIRNSVAPFLLAFQ
Bnapus_EST      VKAWS--YLPEHYQSFYKTPLEF--SLSLNHECLKCLRRLLQKS-TTIRNSVAPFLLAFQ
BnRNLS1-2       VKAWS--SLSYHYQSFYKAPLEF--SLSLDHECLKRVRRIFQNS-TIIRNSIAPFLLAFQ
RsRNLS1-1A      VKAWP--GLSDHYQSFYKAPLEF--SLTLDHECLKCVRRLFQNN-TITRNSVAPFLLAFQ
RsRNLS1-1B      VKAWP--GLSDHYQSFYKAPLEF--SLSLDHECLKCVRRLFQNN-TLIRNSVAPFLLAFQ
Picea_EST       YRAQQ--HIQTSFLKFYGVDFPG-LNFSIDEETINGIQSTLRNV-TLLRNASARIFYNVI
CsRNLS1         EKAYAG-DLKENYLKIYKEQFPI-DAPVLSKACIDGFVWAFQNV-SILKGTLGRFLYNFS
                            :                       .

                                          GmH
OFV_N           ETHH--TNRKRHGLLRFLILQHVDLTGMIPYGMYIDMRRYFTLLTPGQLLTWLHDNQ--V
PYDV-N          GSIL--RNNKNHGFMRFLIFQHAEMRGMQIYKMILTALAGLPAITPAQFLRAIEIPD--A
SYNV_N          TYFR--PTPKIFNVLRFLFFQNLEFMGLHAYVSIVTIMSKV-ALPPSQVLSWLRVSG--S
LNYV_N          NRTPG-LLVTQQGLLNYLACQQFAYPGMHAYTLLIEIHEHT-GMKFSDLLVEMDCPA--T
LyMoV_N         NREDP-LLITQQGLLTYLAGQQFSYPGMHAYTLLIEIHEQT-GIKFGQLLREMDCPA--T
Triphysaria_EST NRGNS-ISKDHLGMLTYLACQMFSYTGMHAYSFIMQIHEKS-GIEYSPLLKQLDCPG--T
PtRNLS3         NEGN--MDRRNSSMLDYLAGQVFSYMGMKGYQLTMDIQKST-MCDMSTLLSELKCRL--T
NtRNLS2         SQAAGTLGTREMWILNYLGFLPFSYNGLHAYTLLIDLKNFS-KVPLGKLMSLFHVNI--T
NCMV_N          NALS--VGDKSFQMVRYLASLPLSLTGMHAYKLFLEVQKQS-NLGMQWLLEEMVSPK--T
LBVaV_Cp        DVADP-------RQTEMLFDQHLANTGMHIIPQFTNAQLSI-GATTAGLLSALNYGQ--N
TStV_CP         DVADP-------RQTEMLFDQHLANTGMHIIPQFTNAQLSI-GATTAGLLSALNYGQ--N
MdRNLS1_1       DL------EEGRGMVTMLFAGHLAYTGMHVVDLFCRAVTGL-QCQPTDLMKALHTHTHTS
MdRNLS1_1FUJI   DL------EEGRGMVTMLFAGHLAYTGMHAVDLFCRAVTGL-QCQPTDLMKAL--HTHTS
Festuca_EST     SL------QDPKGMCKLLYEQHLALTGMHAVNLFVKVCLFT-HCLAEELSSGLWHRT--T
MgRNLS2         EE------KNGREIKDFLYVMHVAYIGLHPYTLFLKCVEDL-KTSDVIFMQAIYSGR--M
Boleracea_EST   ST------EIGKNVKSYLYVTHISFTGLHPLVLFLKCMAAF-KIGSGKLAGSIHTNA--Y
LjRNLS1_2       ET------ETGMDLKEFLYKYHISYTGMHCYRLFIRCMEVF-KVSHEHLVECLHSGTRIR
AqcRNLS1        ET------PLARELREMLYEEHLRFTGMHAYTLFVEVQRAL-EVTIENFGQLLTGTM--Y
Aquilegia_EST   ET------PLARELREMLYEEHLRFTGMHAYTLFVEVQRAL-EVTIENFGQLLTGTM--Y
AqfRNLS1        ET------PLARELREMLYEEHLRFTGMHAYTLFGEVQRAL-EVTIEDFGQLLTGMM--Y
LjRNLS1_1       SN------NRALGLKRFLYDLHLANPGLHVVNISIRLCKVL-NVPSQKLIDIMNVGE--F
LjRNLS1_1B129   SN------NRALGLKRFLYDLHLANPGLHVVNISIRLCKVL-NVPSQKLIDIMNVGE--F
Cichirum_EST    SD------ERYKGFKAFLYDSVLKNTGLHIISLMEQLSDVL-NCPLGSIMITMVHRS--M
BrRNLS1         ELNGC---SKNRAICKTLFESHLGFTGLHAYTLFISNASKL-AVPHHVFRHVLRHHA--A
BrRNLS1-1A      ELSGC---SKNRAICKTLFESHLGFTGLHAYTLFISNASKL-AVPHHVFRHVLRHHA--A
BrRNLS1-1B      ELSGS---SKNRAICKTLFESHLGFTGLHAYTLFISNATKL-AVPHHVFRHVLRHHA--A
BnRNLS1-1       ELSGC---SKNRAICKTLFESHLGFTGLHAYTLFISNATKL-AVPHHVFRHVLRHHA--A
BoRNLS1-1_Ca    ELSGC---SKNRAICKTLFESHLGFTGLHAYTLFISNATKL-AVPHHVFRHVLRHHA--A
Bnapus_EST      ELSGS---SKNRAICKTLFESHLGFTGLHAYTLFISNASKL-AVPHHVFRHVLRHHA--A
BnRNLS1-2       ELSGS---SKNQVICKTLFENHLGFTGLRAYTLFVSNASKL-YVPYHVFRHFLRHHA--T
RsRNLS1-1A      EISG----SKNRAICKTLFESLLGYTGLHAYTLFISNASKL-AVPHHVFRHVLRHHR--A
RsRNLS1-1B      EISG----SKNRAICKTLFESHLGYTGLHAYTLFISNASKL-AVPHHVFRHVLRHHR--A
Picea_EST       DCA-----PADIGMLDMLALIHCAYTGLHSYRHFSTICRVS-NIKYSNLLEWLRYGR--T
CsRNLS1         SLGDG---NVGKGLATMLFEQHLGFTCMH-XSLFIRVASSL-NLSPKDLTKACWHKL--T
                                 *         :                    .

                                                  YaRifdxxxfxxLQtkxC
OFV_N           ---SRPLSVIADINTRYDVS------NGSDRFWRYSRGLDPGFFIALQQSKCVTLIARMA
PYDV-N          ---VKAIKTVMKIATTLDKPGR----QDPTYWWKYGKYIEPSYFVDLSVGRNTKFAYLMA
SYNV_N          ---EMAIDEAFMIMNTLDNGMIDNG-HNAERLWKYARCLDQGYFNRLQSSYSAELIAMLA
LNYV_N          ---RAGVREALELIRDYEITKDH---PKRTTYFRYARNWDPKYFGALQSTECKTLVYVAA
LyMoV_N         ---RAGVKEVLDIIKDFEITKLH---PKRTTYFRYARNWDPKYFGTLQSTQCKTLVYVAA
Triphysaria_EST ---RKAVIEAENIITNFELTPGK---EGRTTYFRYARFFDPGYFLSLQTKNCTMLAYLTA
PtRNLS3         ---RQAVLTIEDVLRHEKL-------PNRKTYFRYSRVFDPGYFSNLQTKKCPALVYLLA
NtRNLS2         ---APALSKIAYIVQNLERTTDN---PNRDVYFRYCSSWGPQYFQSLRSRHCTHLLYTVA
NCMV_N          ---LPALEGIAKILKNFESRTS----TRKPPYFRYARIMSPAYFQELQTKNCPELVYLLV
LBVaV_Cp        ---FGTLMQLKKLINESLSKPPG---PDNRATWRFARIFDPSVFQTLQTKYCADTVAILA
```

```
TStV_CP         ---FGTLMQLKKLINESLSKPPG---PDTRATWRFARIFDPSVFQTLQTKYCADTVAILA
MdRNLS1_1       MRKKSILDELRIIIESYIGSEEE---DKKRRTFQYARLFDSQMFGNLQSKNCAHLVCILA
MdRNLS1_1FUJI   MHKKSILDELRIIIESYIGSEEE---DKKRRTFQYARLFDSQMFGNLQSKNCAHLVCILA
Festuca_EST     ---RDALREIKDILVNHYLSEET---NQQKQTWMYARLMDDARFTPIQTSYCKNLVCALA
MgRNLS2         ---KRDVESLLHVFENLSGKIEDE--EYRLQMWKYGRIFDSRFLSVLQTKKCVYFTWVLV
Boleracea_EST   ---ESELREICLFIERFIDSEDE---EMSFQMWRFARIFDDTFFSTLQTRHCKRLTLALS
LjRNLS1_2       ---NCELKSLGKLFQNCVTNASD---GNRLQMWKYARIFDKSFFVELQTKNCTTLTAVLA
AqcRNLS1        ---VTPFRYMENVIRTFEGDNLSE--NKRRMTWKYARIFDSSFFTGIQTKNCKFLVYLLA
Aquilegia_EST   ---VIPFRYMKNVIRTFEGDNLSE--NKRRMTWKYARIFDSSFFTAIQTKNCKFLVYLLA
AqfRNLS1        ---VTPFRYMENVIRTFEGDNLSE--NKRRMTWKYARIFDSSFFTGIQTKNCKFLVYLLA
LjRNLS1_1       ---RRQAVALAEMIRLVVIKAD----DHKRKMWRFGRIFDSTFMAELQTKACSKLVYILA
LjRNLS1_1B129   ---RRQAVALAEMIRLVVIKAD----DHKRKMWRFGRIFDSTFMAELQTKACSKLVYILA
Cichirum_EST    ---QVLVKSLLHVLKILTENDE----NHKRNMWRYGRIFDEAFLADLQTKSCVEVVYILA
BrRNLS1         ---EEGLETIMEILKRYEDPSLDEGDRKSKCTWIYSRIFDSDMFGSLQTKRCVFLAALLA
BrRNLS1-1A      ---EEGLETIMEILKRYEDPSLDEGDRKSKCTWIYSRIFDSDMFGSLQTKRCVFLAALLA
BrRNLS1-1B      ---EEGLETIMEILKRYEDPSLDEGDRKSKCTWIYSRIFDSDMFGSLQTKRCVFLAALLA
BnRNLS1-1       ---EEGLETIMEILKRYEDPSLDEGDRKSKCTWIYSRIFDSDMFGSLQTKRCVFLAALLA
BoRNLS1-1_Ca    ---EEGLETIMEILKRYEDPSLDEGDRKSKCTWIYSRIFDSDMFGSLQTKRCVFLAALLA
Bnapus_EST      ---EEGLETIMEILKRYEDPCLDEGDRKSKCTWIYSRIFDSDMFGSLQTKRCVFLAALLA
BnRNLS1-2       ---EEGLETIMEILKRYEDPSLDEGDKKSKCTWIYSRIFDSDMFASLQTKRCTFLAALLA
RsRNLS1-1A      ---EEGLETIMEILKRYEDPSLDEGDKKSKCTWIYSRIFDSDMFGSLQTKRCTFLAALLA
RsRNLS1-1B      ---EEGLETIMEILKRYEDPSLDEGDKKSKCTWIYSRIFDSDMFGSLQTKRCTFLAGVLA
Picea_EST       ---RSQVEALDKIMKEYECTQPAQ--NLSRRTWRYGRIYEDGFMKALQTKNCTELCYILT
CsRNLS1         ---KMPLNSLVTILSKVTAESNIAGAPNVRGTWKYARVFGSEYFIGIQSKRCPILFCLLA
                            .                   : :        :  :

OFV_N           HILVKG----G---AVAVNEYSDPRKAKSLEN-----KPGLAAEA------D-KFATEFV
PYDV-N          CILNEM----S---LINGPEYANPKNIKALESIKNNIELTNYYEG------LSRNFSILY
SYNV_N          YIEINM----G---ISTEVGYNSPLNIYAIAN-NKAVKEVGRMKADVFIQCKNSVVSLTQ
LNYV_N          SVSKKV----S---AQGAN--GDPMEIFAIKNLDATIKARLDPVA------E-NMAGKIL
LyMoV_N         SVCKKI----S---AQGEK--GDPTEIYAIKTLDATIKERLEAVA------N-RMAHKII
Triphysaria_EST KTLQKL----T---PQTAG--SNPVDVYALRNIGPGLRAILDEVA------V-NLYEYIL
PtRNLS3         KTYKEI----S---TSTSEA-SDPLSIYGIRNIGDGLKESLDEVS------A-RLVDMIS
NtRNLS2         WAWKTI----A---PGHPN--ADPENIAAISSLSRTMKTTLKQAG------I-IIGESLK
NCMV_N          CLLQKY----E---AFGAG--QEPTKIVGIERVPANIRAEMSRAA------G-YIFSVAP
LBVaV_Cp        NINSMG----K---LSTET--SNPLNIAVLKQMAPERKRYTRQVA------K-NIYHHFM
TStV_CP         NINSMG----K---LSTET--SNPLNIAVLKQMAPERKRYTRQVA------K-NIYHHFM
MdRNLS1_1       YLCQQC----G---SAGA---GDATQIAVIANMGQGAKDNCKAEA------I-NIYNYIV
MdRNLS1_1FUJI   YLCQQC----G---SAGA---GDATQIAVIANMGQGAKDNCKAEA------I-NIYNYIV
Festuca_EST     FMAGQM----G---IAGN---QNVMNIVHIQKMTAAARAIPIRWA------E-RLIQNLN
MgRNLS2         YVLYFN----N---PGSN---EGLLKIAQIQDISEGNKSEAKAHA------K-RALELIA
Boleracea_EST   YLLKLV----A---PDEN---QNVLKIAQIAHVSHVERVLAETFA------Q-RAYQVIV
LjRNLS1_2       YMLKDE----K---AEANG--EDLLKITQISSLSEEAKKFAMAYA------K-KTRSVIQ
AqcRNLS1        YLSILV----G---IHD----ESVLQIVQIRDINELMKTTTQDKA------Q-VIYDSIL
Aquilegia_EST   YLSILV----G---IHD----ESVLQIVQIRDINEFMKTTTQNKA------Q-VIYDSIL
AqfRNLS1        YLSILV----G---IHD----ESVLQIVQIRDINEFMKTTTQNKA------Q-VIYDSIL
LjRNLS1_1       YALKSE----Q---PHGN---ENILDIVQLQNFSPDMKHKLSAAA------Q-KVIKSLR
LjRNLS1_1B129   YALKSE----Q---PHGN---ENILDIVQLQNFSPDMKHKLSAAA------Q-KVIKSLR
Cichirum_EST    EALRSE----S---PEKH---SGIMKITQFADINKNDKREYGESA------Q-RLLTHLK
BrRNLS1         VIADTI----GGT-SSSGGGSGQASNIKQLQGYIQNNQDDLHLWA------G-RIIAFCK
BrRNLS1-1A      VIADTI----GGT-SSSGGGSGQASNIKQLQGYIQNNQDDLHLWA------G-RIIAFCK
BrRNLS1-1B      VIADTI----GGT-SSSGGGSGQASNIKQLQGYIQNNQDDLHLWA------G-RIIAFCK
BnRNLS1-1       VIADTI----GGT-SSSGGGSGQASNIKQLQGYIQNNQDDLHLWA------G-RIIAFCK
BoRNLS1-1_Ca    VIADTI----GGT-SSSGGGSGQASNIKQLQGYIQNNQDDLHLWA------G-RIIAFCK
Bnapus_EST      VIADTI----GGT-SSSGGGSGQASNIKQLQGYI-NNQDDLHLWA------G-RIIAFCK
BnRNLS1-2       VIADCNTLGGGGL-ASSSGGSGQASNI--LQQYIRNNQDDLHLWT------G-RIIALCK
RsRNLS1-1A      VIADTI----GGTSSSSGGGSGQASNIKQLQGYIQNNQDDLHLWA------G-RIIAYCK
RsRNLS1-1B      VIADTI----GGTSSSSGGGSGQASNIKQLQGYIQNNQDDLHLWA------G-RIIAYCK
Picea_EST       QLVILL----G---QKGD---SDPMEVAALKGMGQFSKDFSDRFA------R-LIFEKIL
CsRNLS1         MICERI----G---DMGN---QDPPETRAI----------YRRWA------N-RICDFYM
                                         .   :

OFV_N           EAYNGLSGSSANAGPVSRKLYNQGRGIPTRRGLFTPPSARPAPVVNVHVPAASSSLTGAL
PYDV-N          RSLETESGIGIGIAMQMGGAPAPKARAQKRANEEAPPAAQKRATPAAQQDQQAAGTSGTA
SYNV_N          DASVIDKVYAAAQQKHIRSEEAARPSEQNKEDEVVAMDTDAPSRKRRSDALTTEKPKKAL
LNYV_N          DQMLMDEMSGASWATKASTQ----------------------------------------
LyMoV_N         DQMLVDAMSGEAWVGGN-------------------------------------------
Triphysaria_EST TEGKGKAIEDVI------------------------------------------------
PtRNLS3         KSTVNHKSHHLQPPHHHQHQPFSPASSASNRLQHYSSSHTANTRKPTSRNPITRSITRHH
NtRNLS2         KN----------------------------------------------------------
NCMV_N          QRNMGMYSDSMRKALVHQEKASTSRAGKEKADEVFGM-----------------------
LBVaV_Cp        VVARALNNDMFDTDKYKFVESDDEEEHVANEGETPVKE----------------------
TStV_CP         VVARALNNDMFDTDKYKFVESDDEEEHVANEGEAPVKE----------------------
MdRNLS1_1       TKNEIEK-----------------------------------------------------
MdRNLS1_1FUJI   TKNEIEK-----------------------------------------------------
Festuca_EST     ESDKAADGNELAMESDDDYDDEDEEDNEDDEEE---------------------------
MgRNLS2         KVAKR-------------------------------------------------------
Boleracea_EST   ESDYTGNYKGLEELVV--------------------------------------------
LjRNLS1_2       GSHPISTLAEQDKKKRVREMRDDASVKGKRNIKKKLVRL---------------------
AqcRNLS1        ASTISRLKLK--------------------------------------------------
Aquilegia_EST   ASTISRLKLK--------------------------------------------------
AqfRNLS1        ASTISRLKLK--------------------------------------------------
LjRNLS1_1       SNV---------------------------------------------------------
LjRNLS1_1B129   SNV---------------------------------------------------------
Cichirum_EST    NQNAPRI-----------------------------------------------------
BrRNLS1         EFNEKESTKK--------------------------------------------------
BrRNLS1-1A      EFNEKESTKK--------------------------------------------------
BrRNLS1-1B      EFNEKESTKK--------------------------------------------------
BnRNLS1-1       EFNEKESTKK--------------------------------------------------
BoRNLS1-1_Ca    EFNEKESTKK--------------------------------------------------
Bnapus_EST      EFNEKESTKK--------------------------------------------------
BnRNLS1-2       EFHEKESTTNK-------------------------------------------------
RsRNLS1-1A      EFNEKESTKK--------------------------------------------------
RsRNLS1-1B      EFNEKESTKK--------------------------------------------------
Picea_EST       AAKDGIGEQSAQGSNGEQSAQVKTNKQINV------------------------------
CsRNLS1         PTLFFSFKFAYD------------------------------------------------


OFV_N           DAMNSD--------------
PYDV-N          SATPVDAMNAALQSGVLDQLP
SYNV_N          PAIIKLPNIPDF---------
```

```
LNYV_N           ---------------------
LyMoV_N          ---------------------
Triphysaria_EST  ---------------------
PtRNLS3          PSV------------------
NtRNLS2          ---------------------
NCMV_N           ---------------------
LBVaV_Cp         ---------------------
TStV_CP          ---------------------
MdRNLS1_1        ---------------------
MdRNLS1_1FUJI    ---------------------
Festuca_EST      ---------------------
MgRNLS2          ---------------------
Boleracea_EST    ---------------------
LjRNLS1_2        ---------------------
AqcRNLS1         ---------------------
Aquilegia_EST    ---------------------
AqfRNLS1         ---------------------
LjRNLS1_1        ---------------------
LjRNLS1_1B129    ---------------------
Cichirum_EST     ---------------------
BrRNLS1          ---------------------
BrRNLS1-1A       ---------------------
BrRNLS1-1B       ---------------------
BnRNLS1-1        ---------------------
BoRNLS1-1_Ca     ---------------------
Bnapus_EST       ---------------------
BnRNLS1-2        ---------------------
RsRNLS1-1A       ---------------------
RsRNLS1-1B       ---------------------
Picea_EST        ---------------------
CsRNLS1          ---------------------
```
