# Supplementary material for: Widespread Endogenization of Genome Sequences of Non-Retroviral RNA Viruses into Plant Genomes
Source: PLoS Pathog. 2011 Jul 14;7(7):e1002146. doi: 10.1371/journal.ppat.1002146 (PMC3136472; doi:10.1371/journal.ppat.1002146)
Supplement: Figure S6 — Alignment of a plant nuclear encoded FRLS and betaflexivirus replicase proteins. The partial replicase polyprotein sequences (approximately 1500 aa) from Cuc. sativus (cucumber) (CsFRLS1), all Betaflexiviridae genera (Citri-, Carla-, Fovea-, Viti-, Capillo-, and Trichoviruses) and a member of the family Alphaflexivirdae (potato virus X) were aligned using the program MAFFT version 6. The alignment was used to generate a phylogenetic tree, as shown in Figure 7C. Conserved methyltransferase, RNA helicase (partial), and RdRp motifs are marked in red. (PDF) [file ppat.1002146.s006.pdf]

```
CLUSTAL format alignment by MAFFT (v6.847b)
                                            methyltransferase; pfam01660→
CsFRLS1         MALLSN--KTPIECMLGN----------FENKR---IYEPAVNSIVSNSLFRNSHFAFYM
CLBV            MALMSN--KTAIESILGN----------FEKKHVDAIYNAAAQTILSHSEFRNKHFAYSL
DMV             MALMSN--KTAIESILGN----------FEKKHVDAIYNAAAQTILSHSEFRNKHFAYSL
ACLSV           MAFSY---RTPQEELLSR----------LPQSQQEVISGFQYERIQKEEEKKVENFSFYL
CMLV            MAFSY---RTPQEELLNR----------LPQTQQEIIGNLQFERLQKEEERRVVSFSYAL
PeMV            MAFSY---RTPQEELLNR----------LPQTQQEIIGNLQFERLQKEEERRVVSFSYAL
RuSPaV          MALSY---RPAVEEVLAK----------FTSDEQSRVSATALKALVDLEESQHNLFSFAL
PChMoV          MLKAT---SGPISHGSHSQNASRGGNRPFTSEEQSRVSSTAVISLTEVEKSKHRLFSFAL
PVS             MALTY---RSPIEEVLTL----------LEPNAQSLISNVATSSFQESEKDNFAWFCYHV
PVM             MAVTY---RTPMEDIVNC----------FEPATQAVIANSAATLYKNFEEQHCQYFNYYL
GVA             MSISVSSQRVAVSNLYTN----------GSEESVKAIKELKSKRLLETETRLDGLFDYYI
GVB             MSISVSSQRVASAALYQN----------GSTDQIEEIKKIKSSRLLNSEKHSDGLFDYHV
ChVA            MAFVA---KFAEENYFNS----------LPSNVTDAFLRDGFNAEHNRFEILSRHFAFAL
ASGV            MAFTY---RNPLEIAINK----------LPSKQSDQLLSLTTDEIEKTLEVTNRFFSFSI
PVX             MAKV----REVYQSF-------------TDSTTKTLIQDEAYRNIRPIMEKHKLANPYAQ
CymMV           MARV----RDTLDRL-------------RDPSVLTSINEEAHRHIRPVLASALVNCPYAL
                *                                   .                    :

CsFRLS1         DPFLKKKLTNAGVELFPNGYVVHSHPFSKTLENHLLFDVLPGIIQSEKFLFC-SIKESRS
CLBV            NSYQKKIASKVGIELYPNGYLPHSHPLSKIFENHLLFDVLPGVVNTSRLVMC-SIKESKV
DMV             NSYQKKIASKVGIELYPNGYLPHSHPLSKIFENHLLFDVLPGVVNTSRLVMC-SIKESKV
ACLSV           PEKTREWFTKSGVYLSPFAYVNHSHPGCKTLENHLLFNVVASYISKYSYVACLSIKSNKM
CMLV            PEKTKEWFTKSGVYLSPFSFEVHSHPGCKTLENHILYNIVAPHISKYPYIACLSIKANKM
PeMV            PDKTKEWFTKAGVYLSPFSFEVHSHPGCKTLENHILYNIVAPHISKYPYIACLSIKANKM
RuSPaV          PDRSKERLISSGIYLSPYSFRPHSHPVCKTLENHILYNVLPSYVNNSFYF--VGIKDFKL
PChMoV          PDLAKERLSGAGIYLSPYSYQVHSHPCCKTLENNILYNVLPSYLDNSFYM--VSIKKNKV
PVS             SASAKEHLSRAGIYLSPYSGYPHSHPVCKTLENYLLYKVLPPLVNNTFYF--VGIKEFKL
PVM             SPLAKRKLSMAGIYLSPYSAVVHSHPVCKTLENYILYSVLPSYINSSFYF--VGIKERKL
GVA             PDTLREILTGYGMEFSVHSFQGHAHPVSKMIENHMLYRVAPNYFSSNTLV--VSCKESKI
GVB             PDFLRDYFAKKGVHTSVHSFQAHPHPCSKMIENHILYNIVSQYVDDSTLF--VSCKESKL
ChVA            KPSQRTYLNDCGIQLAPIASKTHPHPVSKIIENHLLYCVVSNMISNFKFLVFLSIKEVKA
ASGV            TPEDQELLTKHGLTLAPIGFKSHSHPISKMIENHLLYICVPSLLSSFKSVAFFSLRENKV
PVX             TVEAANDLEGFGIATNPYSIELHTHAAAKTIENKLL-EVLGSILPQEPVT-FMFLKPRKL
CymMV           TEEEADCLENLGVTVNPFAIQTHTHAAAKTVENRML-EIVGTHLPKEPST-FIFLKRSKL
                           *:     .   *.*. .* .** :*       .           :  :

CsFRLS1         -KLFRQ----------DQKE-----EVTXRFLNRLIDGRDITRYPEVGFEGNFGLNAVKG
CLBV            -LVFKGIRDKSRRQVSDLNALNSLNNSHTSFINRLVASKDVSRYTE---EADAFFQSKKG
DMV             -LVFKGIRDKSRRQVSDLNALNSLNNSHTSFINRLVASKDVSRYTE---EADAFFQSKKG
ACLSV           -SKMER------LGPNSVK------TYD--ILNRLVTAKDKARYG---------------
CMLV            -SKMER------MGAHSVK------NYD--IFNRLVTSRDKSRYG---------------
PeMV            -SKMER------MGPHSVK------NYD--IINRLVTSRDKSRYG---------------
RuSPaV          -QFLKR------R-NKDLS------LVA--LINRFVTSRDVSRYGS-----EFVISSSDK
PChMoV          -DFLKQ-------RHKDLQ------LIE--CINRYVTSLDKVRYGS-----EFHISPSKK
PVS             -NFLKK-------RIKQMS------MIQ--AINRYVSSADKLRYGN-----EFVIKFGAA
PVM             -QLLKS-------KCKNLD------SVQ--VVNRYVTSADRMRYTN------DFVPYGSY
GVA             -KRLRL------KNANNRNL-----NFT--QYNRLVHANHHHRYENAFRELDV-------
GVB             -RTLFM------KRPKSLS------NVT--QYNRLVHAKDVLRYTDPVRELDM-------
ChVA            -EYIWN---------KNTA------DTVREISNRILDIKDAFRYGP--------------
ASGV            DSFLKMHSVFSHGKIKSLG-----------MYNAIIDGKDKYRYGD--------------
PVX             -NYMRR-----NPRIKDI------------FHNVAIEPRDVARYPK--------------
CymMV           -RYLRR-----AANNKDI------------FRNQHIEPKDLLRYDD--------------
                   :            .               *  :   .  **

CsFRLS1         NF-----------------KFSRQFIRTIGKRKNIFFHDEVHHWDQECVFKFL-DELEPR
CLBV            SPEL----------------FSRNFIKSLENKEAVFFHDEVHHWTKAQMFSFL-KSTKVK
DMV             SPEL----------------FSRNFIKSLENKEAVFFHDEVHHWTKAQMFSFL-KSTKVK
ACLSV           -------------------PLAKPERSPCPKKTNIFIHDEIHYWSRDQLETFL-QVHRPK
CMLV            -------------------DLTQPTRVGGPKGSNYFIHDEIHYWSRTQLETFL-DIKKPR
PeMV            -------------------DLTEPSRVGGPKSTNYFIHDEIHYWTRKQLETFL-ELKSPK
RuSPaV          SSQVVSRKGIGDSN--TLRRLVPRVISTGAR--NLFLHDEIHYWSISDLINFL-DVAKPS
PChMoV          NPHFNCARKVGFENDPSIKDLLPQCVSDSSR--RFFLHDEIHYWSKEALINFL-VSVQPE
PVS             SPELKRHHGYSLD--PALRDLLPNIKRDS----NLFFHDEMHYWEKNQLIHFL-EQCRPN
PVM             EHECLVHKGVGLDN-EALRGLVGPLRRHKAK--NLFFHDELHYWSSKVLIDFL-DVMRPD
GVA             -------------------GNLTNLINKEDQSECIFIHDEVQYWSLDEMQRFLGSLSKVD
GVB             -------------------EHLVKLSELSKRAKTAFIHDEVHYWSLKDFQSFLGHLGGTQ
ChVA            ---TNTVNGGINNFSFFCANLNRRFNNRAIKPDCFFIHDEVHFWSPAELCEFL-FTVEPK
ASGV            --------VEFSSFRDRVIGLRDQCLTRNKFPKVLFLHDELHFLSPFDMAFLFETIPEID
PVX             ---------------ETIIDKLTEITTD-----TAYIGDTLHFLDPSYIVETFQNCPKLQ
CymMV           ----------------ESCEVMPECSTS-----TAYISDALHFLSYAQLGKIFQNSPKLK
                                                   :: * ::.     .   :

CsFRLS1         RLVFSVVYPVELLGNYASSQNPKLYTFKIVGKKLFFFPDGITSEGYEQQV-NLNWLFRSN
CLBV            RFIFTVVYPPEILKKFANSQNPKVYDFKVDKGRLFFFPDGVKTEAYEQKL-NMEWLFSAS
DMV             RFIFTVVYPPEILKKFANSQNPKVYDFKVDKGRLFFFPDGVKTEAYEQKL-NMEWLFSAS
ACLSV           NLWATLVFPPEILAGYKSSVLPFLYQFEIHGKDLVYMPDGVRSESYTQPL-ENGFLLSSS
CMLV            NLWVTMVFPPEILKGYKSSVLPFLYEFEIDKGNLIYMPDGSRSESYTQSI-ENGYLLSTN
PeMV            NLWVTMVFPPEILKGYKNSVLPFLYKFEIEGDNMVFMPDGVRSESYTQPI-ENGYLLSTN
RuSPaV          MLLATAVIPPEVLVGSPESLNPWAYQYKINGNQLLFAPDGNWNEMYSQPL-SCRYLLKAR
PChMoV          MILATVVYPPEILAGAKESLNPWCYKFQINGDDLIYAPDGCMQEAYIQPL-SGCYLLKTS
PVS             TCLCTIVYPTGIFVGARRSLNPWAYEFEIKRDKLLFYPDGVRSEGYEQLV-NCGYLLRTR
PVM             KLLGTVVYPPELLFKPTRSLNEWCYTYDIVGDTLMFFPDGVQSEGYQQPL-KGGYLLGAR
GVA             RVVYSIIYPSEVEAGYSQSLFPEAYTFDLKDRRLVWYPDGKAEGAYTQPV-N-PWLLRCS
GVB             RLLYSVIYPAELHIGFEQSLFPEAYEFDLLGEYITWYPDGKADGSYKQPV--NPWLLTTS
ChVA            NVLATVVIPPELLEGLDYSFNSVAYDFKKVDGNLYYFPDKSKGKPYQQPM--DPWLLKCN
ASGV            RVVATTVFPIELLFGDKVSKEPRVYTYKVHGSSFSFYPDGVASECYEQNLANSKWPFTCS
PVX             TLYATLVLPVEAAFKME-STHPNIYSLKYFGDGFQYIPGNHGGGAYHHEFSHLQWL-KVG
CymMV           ILLATLVLPVEALHRHP-SLYPAIYTLNYHKDGFEYIPGNHAGGAYFHEYSTLQWL-TLG
                    : : *         *     *  .     : : *.      * :      :

CsFRLS1         YLVDRK------------G-----RKWTIIRHCSKFSHHMFEIV-PGEFQTESKFFFKDF
CLBV            HLRSGD------------------CVWTVTRHKSIYAHHLFEIS-IGELVTDSKLFFSDY
DMV             HLRSGD------------------CVWTVTRHKSIYAHHLFEIS-IGELVTDSKLFFSDY
```

```
ACLSV           SIIIKN------------RVTGVEIRYQVSLVYSLGSHHLFHIYPAEDLMKEEVRRFGPY
CMLV            CISFKN------------K-DGLRKQYSVTLVYTLGSHHVFHIFPNMGLIEDEIRRFGPY
PeMV            CISFTD------------S-NKRKRQYSVTLVYTLGSHHVFHIFPKLGTMEDEVRRFGPY
RuSPaV          SVVLPD------------G-----SRYSVDIIHSKFSHHLLSFTPMGNLLTSNMRCFSGF
PChMoV          GIELPD------------G-----SFYSLDLVKSTFSHHLICIT-KGKLINQKMRPFSGF
PVS             KILLRD------------G-----TMYSVDLVCSKFAHHLIAIT-KGDLITPTYRSFGPF
PVM             SLKLPD------------G-----TVYMVDVLCSKFPHHLISIT-KGEAAAPTHRAFGPF
GVA             KTEDSK------------G-----RSWTITKLQTIGAHHLFSAI-KGSYLTEESYKYDNF
GVB             KTVDVH------------G-----QTWTLTKVLTMGAHHLFLAT-RGSTLTEEEYHYDDF
ChVA            KISMIK--------------NGEIFSYSIGLLESVGANHLFSF--QRNKVVESTRFFNDF
ASGV            GIQWAN------------------RKIRVTKLQSLFAHHVFSFD-RGRACNE-------F
PVX             KIKWRDPKDSFLGHLNYTTEQVEMHTVTVQLQESFAANHLYCIR-RGDLLTPEVRTFGQP
CymMV           KLIINDPLKVKKP-----------LTLTVQLIESLGANHLLLIT-RGDLRTPKLRTFAKD
                     .                      :    :  .:*:
                                                       methyltransferase ←
CsFRLS1         D-VVDMSVMFR-NRFRYY-ELFP---VNFQRLYKVYSYLLCLKKPDLKSGLAKLRQIMGD
CLBV            N-SIDMSKIFL-DRFRSY-EVFP---ISIEHLYKVYSYLLCLKKPDLESGLAKLRQIIGD
DMV             N-SIDMSKIFL-DRFRSY-EVFP---ISIEHLYKVYSYLLCLKKPDLESGLAKLRQIIGD
ACLSV           D-LFDVGSLFV-KPVRVPIQDFP---LS--VFKKIFIYMSSLKKPDVQSAVAKLRQLSDA
CMLV            D-LFDVGTLFK-KPVRVPIQGFP---LS--TFKKIFIYMSSLKKPDEQSAVAKLRQLCDA
PeMV            D-LFDVGVLFK-QPVRVPIQDFP---LS--TFKKIFIYLSSLKKPDEQSAVAKLRQLCDN
RuSPaV          D-AIGIKDLEP--LSRGMHSCFP---VHHDVVTKIYLYLRTLKKPDKESAEAKLRQLI-E
PChMoV          E-ATSSKGISP--LMRKVGSCFP---IHPNLILRIYRYLRTLKKPDLQSSMAKLSQVL-P
PVS             E-AIKSAGLQG--ISKGRPKFYP---VPCHMISRLYRYLRSLKKPDKQSAMAKFSQMC-P
PVM             E-AVASEALKA-TLSPDYPCAFP---VSYEVVNKIYRYLRTLKKPDEQSAIAKLSQII-A
GVA             T-IINPNDVLK---GKRGGKPLY---LRARMIKPTLLYLLALKKSDSNSAVAKLRMLSSR
GVB             T-VILPKKLLQ---GRRRNKMPY---LRSRFITSVLLYLLALKKPDPSSAVAKLRQLTNG
ChVA            D-CLDMRKLLPINVENGKIKGYN---IRTWVFKKILSYIVCLKKGDSESSLAKLRQLSDS
ASGV            N-HFDKPSCLLAEEMRLLTKRFDKAVINRSTVSSLSTYMACLKTANAASAVAKLRQLEKR
PVX             DRYVIPPQIFL---PKVHNCKKP---ILKKTMMQLFLYVRTVKVAKNCDIFAKVRQLIKS
CymMV           T-HVLLPQIFH---PKGMNANKP---LSKRRAMQLWLYAKSVKEVSERDLYAKVRQLIPT
                                          :          *   :*  .  .  **.  :

CsFRLS1         EC----KIKEFLFFEQFCKIVIDRSTTYGLFGHTLFESITSNLIRMLPDF--MARTTTTW
CLBV            DV----EIKEFLFFEQFCKRLIERQTSWGLFGHSFFEKLTDMALSSLPNS--IARIFPQW
DMV             DV----EIKEFLFFEQFCKRLIERQTSWGLFGHSFFEKLTDMALSSLPNS--IARIFPQW
ACLSV           DI----SIESVFMIQEFASRIEKNGVESWSC--SFWGCMKDWFFDKLPYR-------EVL
CMLV            EI----SIEAVFLIQEFAKRIEKGGILSWSC--SFFDYLKDHFFDKIPFK-------DTL
PeMV            EI----SFEAVALIQEFARRVEKGGIQSWGC--SFFDYLKDFFFDKIPFK-------GTL
RuSPaV          KP----TGREIKFIEDFSSLVINCGRSGSLLMPNISKLVISFFCRMMPNA--LARLSSSF
PChMoV          NP----TGPEIKFFEEFSKLLLNCETVNTKLLPNVWKLISNFFIGLFPDS--VARNFKVF
PVS             EP----SGDMIRFIEELSDLIINTGTLRVMIDAELCKNFFGNLGLALPAT--LASKIKST
PVM             EP----SGREIDFVECFARLVIHNSSMCATIMPEQLKEFMGNWLGKMPSV--LARRFSSV
GVA             EE----NMDEALFVAQLAKQIKDTALYDKMGNPNLRSILSESFYDIAGSL--FTRLFNRP
GVB             EE----STAEALFVAQLSRQIQETKLYDKMGNFSLKECIWSAVSSAMGDS--LVYFFDKA
ChVA            SP----SSDELLLIGDFFDLMTRVKIFNKRSPWSFLSDAKNYVDSWIIQSPFLRRIFPVG
ASGV            DL----YPDELNFVYSFGEHFKNFGMRD-DFDVSVLQWVKDKFCQVMPHF--IAASFFEP
PVX             SDLDKYSAVELVYLVSYMEFLADLQATT-----CFSDTLSGGLLTKTLAP--VRAWIQEK
CymMV           SELDLFDPVEVTHLVNYLLFISHLSSVS-----SYDDILSSNIFQHFTIP--IKNKIREL
                .            .      .

CsFRLS1         R--KMNL-FEFLFSL---DTLTIEVN---RGTCYDGIGSEIEFQILKLDPRCLVDPLYLM
CLBV            K--KKNT-FEFLFSL---GTLVVDVE---RKVCFEHVLEEWGFEVVITDENAYLDPLSIF
DMV             K--KKNT-FEFLFSL---GTLVVDVE---RKVCFEHVLEEWGFEVVITDENAYLDPLSIF
ACLSV           E--KIGLADDFTRRLMKIKPLAFDIH---T----------------------TDRPLTVR
CMLV            E--KIGLADDFTRRLINLKPLSFDIR---A----------------------DQERKLIA
PeMV            E--KIGLADDFTRRLINLEPLSFDIR---A----------------------DQERKLIA
RuSPaV          R--ECSL-DSFVYSL---EPFNFSVN---LVDITPDFFEHLFLFSCLNELIEEDVE----
PChMoV          Q--QMSL-DNFISEM---EEFNFSVQ---TSTISLSWLDDIRFVDLNFDIKPVDID----
PVS             R--AVSL-EAFIASL---EPLVVDCE---LQTIS----WAVPLAQLLFSESPDDPPEDMI
PVM             R--AVCV-NKFIRGL---KPYSFTLR---LNEITWWNIWENSYAWFFDTDAEVDVP----
GVA             EYDARCL-EKFIRSC---ETTEIHVERRYMEGIRRGASFKVQNV-MDWVEDDSANALSEV
GVB             RFYNESL-ESFILNC---GPAKIEIKRVYRDAVYRNQGVCPHLVSFDGSPDGVMDELYLQ
ChVA            SRAITELIRDWIANA---ESLKIQTT----------------------------------
ASGV            TEFHLNM-RKLLNDL---ATKGIEVP----------------------------------
PVX             KMQLFGL-EDYAKLVKAVDFHPVDFS----------------------------------
CymMV           VQLFTGA-DQFNQLLKALDWQTFSYS----------------------------------
                                      .

CsFRLS1         EINDNFSEHRSDDKCLERVHLKGWGYKESGKRSIYELAS------------------EQL
CLBV            AINENFNEDRVDDGYLERIRLPFWNLNDYDLKRKRVNAY---------------------
DMV             AINENFNEDRVDDGYLERIRLPFWNLNDYDLKRKRVNAY---------------------
ACLSV           MIIDQIW----EERLSSFDNISN-----IVFYGRREWLN-----NGV------LPKVKKK
CMLV            MVFDAVW----PDQQSE-DYLAA-----EISKGKKRVINGNAFVNGVSSTKNFLL-----
PeMV            MVFDAVW----PDQLSE-DYLAA-----EISKGKKRVINGNAFVNGVSSTKKLFAWEEKG
RuSPaV          EVMDNSWFGLGDLQ-FNRQRAP-------FFLGSSYWLN---------------------
PChMoV          LMFQKGWGLVKEVTILERSREPYSFYKFDSY-----------------------------
PVS             EAMDRRWV-SSSTMLCDRVPAPYRGNMWSET-----------------------------
PVM             EKLDSLFMGEGAGLVAHITSRPYVG-----------------------------------
GVA             NFLDISWNDRV-------------------------------------------------
GVB             HIRDMDRS----------------------------------------------------
ChVA            ------------------------------------------------------------
ASGV            ------------------------------------------------------------
PVX             ------------------------------------------------------------
CymMV           ------------------------------------------------------------


CsFRLS1         NSYSNRVGKKASSFGPLGKLENTHIKANPCVNLAIGWVEPGPFETVDEIF---------L
CLBV            ---------NILSYRFEEERKIESAQKGPNKMLQIEWYGIKEFKVDPFISN---------
DMV             ---------NILSYRFEEERKIESAQKGPNKMLQIEWYGIKEFKVDPFISN---------
ACLSV           GLAKLIPGREVDSHNYPREIYSDLLSSTSIWRSY----------DDDFRHRSA-SPLV-I
CMLV            GRAREV------QPDYPEDFQSDDLAHTPIRLSG----------DEDYSRSAPRTPAI-F
PeMV            GCSRIT---------YQKSFQSDDLAHTPLRISQ----------NLDYSSRCTKNPCY-F
RuSPaV          --------SKFSV---EHKFSGTINSQIMQVILS----------LIPFSDDPTFRPSSTE
PChMoV          --------------------------------------------DAQFTSLVPSTNFEGF
```

```
PVS             --------------------------------------------SRAMSFWSIDFQRIKF
PVM             --------------------------------------------TVPLADREWNALLCMD
GVA             --------------------------------------------SEPYGIECIH------
GVB             --------------------------------------------SEPY------------
ChVA            ------------------------------------------------------------
ASGV            ------------------------------------------------------------
PVX             ------------------------------------------------------------
CymMV           ------------------------------------------------------------


CsFRLS1         SKEHFGKELSTFKRKG-------KGNFFFXEKIVKYIVNNALLVDECDFDCIMHKIDYN-
CLBV            ------------------------------------------------------------
DMV             ------------------------------------------------------------
ACLSV           LRSNRA-YSEAAKFSS-------NCLSLCAAPCDEVIARTPFELN------HRREK----
CMLV            -RTARTEYNVLPPSSG-------APLRIGFERFYGKVEKTEYQKD------LESRK----
PeMV            PEMARTDYVPLPPSYG-------APLCIGFEGFYGNIPKTDYQLK------KEKER----
RuSPaV          VNLALSEVKAALEATG-------QSKLFRF------LVDDCAMRE------VR-------
PChMoV          AKALISYIIRSFK-----------------------------------------------
PVS             LRGLMELYVDSMCTEG-------LATSVTFESYVAQIASSCSLLG---------------
PVM             SQKLLHAMRRMFMRGAWGAHMCVISREFLLKYVEARLKSSCLIAK------ARRRGQHKE
GVA             ------------------------------------------------------GEGSRI
GVB             ------------------------------------------------------------
ChVA            ------------------------------------------------------------
ASGV            ------------------------------------------------------------
PVX             ------------------------------------------------------------
CymMV           ------------------------------------------------------------


CsFRLS1         -----HISHLECMSFKDHEENKKGSAESVAVNCNSNEIEVDICSN---------------
CLBV            -----SITEFTLLEALLGKRIDPKKYSYSKQACTLSNYLTFLCAE--------------G
DMV             -----SITEFTLLEALLGKRIDPKKYSYSKQACTLSNYLTFLCAE--------------G
ACLSV           -----KELSLKCLDFHIKKMK---------------------------------------
CMLV            -----RKIALSALDFHVNKTK---------------------------------------
PeMV            -----KDVSMMVLEHHVKKKN---------------------------------------
RuSPaV          -----SSYKVGLF-KHIKALTH------------------CFNSC--------------G
PChMoV          PLLPCSWYFENVRDMAIGVLVKGGLGKSVSISCFTADLRALFDSSVKPKLHQPPPLIFWE
PVS             -------------LALIKCLTAAEYAEVARIVSNTRLIDVLFTAE--------------D
PVM             KLEAWEVLGLKSSDALFRAMTYLCNAR----------LEPMFSES--------------G
GVA             RVPLSRILRAHELIAGVQTDVEINFPRYVCSSRALIHFRQYLIKL--------------G
GVB             -----SLTSAEVLESSIGCRISLIEMGRAMLKFHQQQDVMINERV--------------V
ChVA            -------------------------------------CSSLTFSD--------------S
ASGV            -----------------------------LSVIILDKVNFIETRF--------------H
PVX             -------------------------------------FKVETWDF--------------R
CymMV           -------------------------------------MPVETIHT--------------R


CsFRLS1         ---FIGERYVRDVP------------------EGAAEETISVLTND--------------
CLBV            LDGFNLEEHLERRL------------------KAAGHDVSDDEEEELTSAEQAGPIKILA
DMV             LDGFNLEEHLERRL------------------KAAGHDISDDEEEELTSAEQAGPIKILA
ACLSV           ---------VKNV---------------------LELE---VKLRE--------------
CMLV            ---------NKDVE------------------SYLAAQ---GRRDE--------------
PeMV            ---------MRNVD------------------EYLASQ---VRVDK--------------
RuSPaV          LQWF-LLR-QRSNL------------------KFLKDR---ASSFA--------------
PChMoV          VSVLWFFKRNRINC------------------HFLREYSE--------------------
PVS             LRWFHATRHSRHNV------------------KFLDETADWARYKS--------------
PVM             LRFFLTR--GRNNL------------------YGLTNYTEGKRAVTGVQ-----------
GVA             RFSFMESRAIKDIE------------------DIQAGLEEGVITEE--------------
GVB             LSPLDDFRSMRKALTDRDGILGTTCLLLEWEYDPLKRYSMGLITAT--------------
ChVA            FEMIKTDSISEFGE------------------HILGNISGGLSSAI--------------
ASGV            ARMFDIAQAIGVNL------------------DLLGK-----------------------
PVX             FHPLQAWKAFRP------------------------------------------------
CymMV           AANYQVAKTVRMCR------------------DLPCDEYDRVKDVL--------------


CsFRLS1         ---------------DAEKSNQFRESHEEDESQISKRLDYKSMFKQIQCDKIHGEFVDVP
CLBV            DPLGFMKECLEEIPIETEPSLEERGQFSTDYHSEKFEINYNDIFNPHNCMNTHGDEIPTP
DMV             DPLGFMKECLEEIPIETEPSLEERGQFSTDYHSEKFEINYNDIFNPHNCMNTHGDEIPTP
ACLSV           ---------------RN-------TRISLSKKGVKKAGRSRMIPVHLL------------
CMLV            ---------------QTRLKNLALTRLSKVKGRVR-----------FV------------
PeMV            ---------------QNKIN---MGRLSANSGKEKRQGYAVIVNGEWV------------
RuSPaV          ---------------DLDCEVIKVYQLVTSQAILPEA-----LLS--L------------
PChMoV          ---------------DYCRADFRSFKIFKSRKFLNEVSNGSKLLNHCT------------
PVS             ---------------EFECATYAKPKGTGHVGYLQNT-----------------------
PVM             ---------------NLWSNVVHEVSTKRHKGMIRLE-----------------------
GVA             ---------------EAELRLLPTTKPKITEIHMDDD-----------------------
GVB             ---------------ELNAAIIGTTKITAKKEMAKEC-----------------------
ChVA            ---------------EACRNWLTSKRFSGGSYSM--------------------------
ASGV            ------------------------------------------------------------
PVX             ------------------------------------------------------------
CymMV           ------------------------------------------------------------


CsFRLS1         HDGNCFFHAIILTFEINCNHIKLRDSFSNWLL---DQGMDKLSEVIKPNGVFMEHELVHL
CLBV            SDGNCFFSAFTETFEVE-RPDTLRSDFSDWLMEFNGGSYASLAEMIRPNGVFMEAELIYL
DMV             SDGNCFFSAFTETFEVE-RPDTLRSDFSDWLMEFNGGSYASLAEMIRPNGVFMEAELIYL
ACLSV           ------------------KPTC--GEGNGKPE----ERNKEEAKIPMNEGTSKEEKGSEP
CMLV            -------------------PTCTASKSSEKPI----GKQQEEKKNELVPKTGHQDSTTLP
PeMV            -------------------PT---KNPNIVPV----LKGAAEKGKA-------QTEEVLT
RuSPaV          ------------------TKVFVRDSDSKGVSIPRLVSRNELEELAHPANSALEE----P
PChMoV          ------------------SPIFFGLESSSTLS----TITDEVPIPCTPKSVTPDA-----
PVS             ------------VYSFHGVGARWSFDPSYSCESDSEATHSDYSVVEYPKAAPIHSERTFP
PVM             ----------------------------------------KARVTEQPRSEFASC-----
GVA             --------------------------------TPGTSGESDVEKFKSVRSLCREE-----
GVB             ---------------------------SERYQQCSCGVRLPMRKLETPLKVPTEE-----
ChVA            ------------------------------------------------------------
```

```
ASGV            ------------------------------------------------------------
PVX             ------------------------------------------------------------
CymMV           ------------------------------------------------------------


CsFRLS1         FSFFIKKIIVV----HFNETVFMFGDEGVEGHIWCDGTHFMAMETYTINKRRDMSILGQI
CLBV            FCVFRGVTLIIHDRTHEKENVYAVHRGFEEGHMVHRGNHFVGIETYNISTLTSDPLLGDI
DMV             FCVFRGVTLIIHDRTHEKENVYAVHRGFEEGHMVHRGNHFVGIETYNISTLTSDPLLGDI
ACLSV           ---------PHSEVKREGVRLDE-------QHISEPLLSFKLDDFVGREKLCSAGLIKTV
CMLV            RDEFINSILKKSEVVREKIEATEVKEFDCSKFILEP--------------VCKHDLMRTN
PeMV            RTDFIESILEKAKLDNRGKSPEDAEMTDVHKYILEP--------------ICDHLIQKCN
RuSPaV          QSVDCNAGRVQAS-------------------------------------VSSSQQLADT
PChMoV          --------------------------------------------------LSDSNFPTFI
PVS             RGRDVEGVGCACGLQSATRVLEF-------------------------------------
PVM             --------------------------------------------------VLEPEVWRDV
GVA             ------------------------------------------------------------
GVB             ------------------------------------------------------------
ChVA            --------------------------------------------------VSRNGLLVDS
ASGV            ------------------------------------------------------------
PVX             ------------------------------------------------------------
CymMV           ------------------------------------------------------------


CsFRLS1         EVKRSDDLINFIKDFNETDYDVFNWHG---------------------------------
CLBV            PCGFSEEITKFHFRPDHFNCAQFRG-----------------------------------
DMV             PCGFSEEITKFHFRPDHFNCAQFRG-----------------------------------
ACLSV           GNDYLLLARQI-------ECMP--------------------------------LSQLRG
CMLV            EQVSALVRQSL-------NALC--------------------------------FRPNKG
PeMV            EKMDGEVLNAL-------LSLG--------------------------------GRELNG
RuSPaV          HS-----LGSV-------KSSIETANKAFNLEELRIMIRVLPEDFNWVAKNIGFKDRLRG
PChMoV          HSCGNLRCAKL------------------------------RSDCTAIPLNLDFPDKINN
PVS             ----------------------------------------------PTEHGFNLEKGVPG
PVM             EAALDIELGEV---------------------ACACNARFVQGVVLSNQAGLNVREQVAG
GVA             ----------------------------------------------------IYSEKLKG
GVB             -----------------------------------------------------FTDALKG
ChVA            MRQNTSFSCEV------------------------------------------FVDLFPS
ASGV            ------------------------------------------------------------
PVX             ------------------------------------------------------------
CymMV           ------------------------------------------------------------

             2OG-Fe(II) oxygenase superfamily; cl01206 (AlkB)→
CsFRLS1         -RRSAFLSNCG-ADYGHNGMTYPINAWNERLDQLVNACCK--NFKYNSALIQ-WYDKGAS
CLBV            -RKAAFITKVD-ADYGHNGMVYPHNSWVPSLEEIIQICGQ--GDDFNCALIN-FYEANSS
DMV             -RKAAFITKVD-ADYGHNGMVYPHNSWVPSLEEIIQICGQ--GDDFNCALIN-FYEANSS
ACLSV           -KKAAYFCLDFPMVYFHDKVSYPTFEATGEIRHVMMKARSKWGIDFNSALIQ-VYNDGCR
CMLV            -KKSVYVTLDSPMVYFHNSISYPSIEATGLIKDWILSKAEDYGVPFNAALVQ-VYEKDCI
PeMV            -RRAMYVCLDSAMVYFHNTISYPSIEAHGKIKDWILRKAKDYDVPFNAALVQ-VYRKDYT
RuSPaV          -RGASFFSKPGISCHSYNGGSHTSLGWPKFMDQ-ILSSTGG-RNYYNSCLAQ-IYEENSK
PChMoV          TRVACFYSRRG-DGYSYTGFSHKSMGWPDFLDKFLLDNQIA-LENYDHCLVQ-KYSQGAA
PVS             -KRAAWYCRGQ-IDYISGAIRLENLGWPRWLSQWMELHEID-ETYYNSMLAQ-EFPAGGT
PVM             -ASVGLYTKDR-SNLKWGNSELLSNGWGRSLSVWMEINSV--SQKFDVAVRL-SYSKETQ
GVA             -REVAFYSRHS-KEYKYNGGSHRSLGWDEALNELTQELGL--DDSYDHCLIQ-RYTAGGS
GVB             -RRAAFYSRHS-WNYSYTGANHASKGWPEWVSNIAEKLGL--GEDFDHCLAQ-IYEEDKG
ChVA            TIRPAFYSDPN-----FTKVESFEPEWDFLLGSWI----------FNHKQVRLCYEPS--
ASGV            ------------------------------------------RFDYEAESEE-YFSENGY
PVX             ------------------------------------------REVSDVEEMENLFSDGDL
CymMV           ------------------------------------------RQLPDGVT---LFEENDK
                                                              :       :

CsFRLS1         I--GFHKDNEKVYG-DDPILTIN---LIGECNFKVE-----FEGEVEEFTMKSCDFFIMP
CLBV            L--GFHRDNERVYN-DDPILTVC---TFGEGRFTIE-----FKDQVTSFLMTAGSFFLMP
DMV             L--GFHRDNERVYN-DDPILTVC---TFGEGTFTIE-----FKDQVTSFLMTAGSFFLMP
ACLSV           L--PLHSDNEECYD-DDGILTIN---VVGDAKFHTT-----CHDE--VIDLKQGNEILMP
CMLV            L--GMHKDNESCYG-NHPILTVN---VSGKAVFSTD-----CCGN--TMELDSGDELLMP
PeMV            M--GMHKDNEACYG-EEPILTVN---AFGSAVFSTD-----CCNN--VINLEDGDELLMP
RuSPaV          L--ALHKDDESCYEIGHKVLTVN---LIGSATFTISKSRNLVGGNHCSLTIGPNEFFEMP
PChMoV          L--GWHSDNEDCYDLDHQILTVN---LCGEAKFAVK-----CGAGSDQVDLLPWDAFIMP
PVS             L--ECEVGDGGQFIPGSNVAIAE---VGGQSQVSIG-----CMAGTGQLLLELGDFIEVP
PVM             M--NVLLPSLDGIERGAGATVVN---LRKCGAFIVR------CARGWRLALAWMDHICLE
GVA             I--GFHADDEPCYLPGGSVVTVN---LHGDATFEVK---ENQSGKIEKKELHDGDVYVMG
GVB             I--NFNADDEPCYT-DPEVVTVN---LNGNANFHLK------CGSESVVPLSDGDVLIMP
ChVA            ---NSHADSEE---------DVN------------------RTSETSSPEKTIADSSSVS
ASGV            IFMPSKSNPERNWILNSGSLKIDYSRLVRARRFRLR------------------RDFLDP
PVX             L---------DCFT-RMPAYAVN--------------AEEDLAAIRKTPEMDAGQEVKEP
CymMV           Q-DPSSSEAEDSED-DTDSVDFN---LPPTHDLPPN-----------FDPLDKGKSIIVD

                                       AlkB ←
CsFRLS1         -KGFQKRTRHSVSAKTK---RLSITFRLHKRMMNGSAI----------------------
CLBV            -KGFQKKARHSVSNEMS---RVSITFRKHVRRLNG-------------------------
DMV             -KGFQKKARHSVSNEMP---RVSITFRKHVRRLNG-------------------------
ACLSV           -AGYQKKNRHAVEVASEG--RTSVTLR---------------------------------
CMLV            -EDFQRKFRHGVKSITDG--RMSVTLR---------------------------------
PeMV            -AKFQRKFRHGVKSLSEG--RVSITLR---------------------------------
RuSPaV          -RGMQCNYFHGVSNCTPG--RVSLTFRRQK--LEDDDLIFIN----PQVPI------ELN
PChMoV          -HGFQVSHKHCILDTSEG--RVSLTFRKSKACLNGISRTLVQGAQGPGIPTPSDASHSLA
PVS             GPCWSKHHLHMCCSEVRG---VTFIFRRIKVPDPVVNAPAVQIAAPATTPGAGSSKPNEN
PVM             -VMANVAYGHECYMRSWGTMDVVVFLKRA-------------------------------
GVA             -PGMQQTHKHRVTSHTDG--RCSITLRNKT------------------------------
GVB             -KGFQKTHKHAVTGTSAG--RISLTFRNGINAPDE-------------------------
ChVA            -PFVSSNHEEGTSS----------------------------------------------
ASGV            -ISKGKSPRKQLFLESTGNIKSNPNAEKN-------------------------------
PVX             -AGDRNQYSNPAETFLSKLHR---------------------------------------
CymMV           -TDNPSTSTAPAVTFAAGINS---------------------------------------
```

```
CsFRLS1         ------------------------------------------------------------
CLBV            ------------------------------------------------------------
DMV             ------------------------------------------------------------
ACLSV           ------------------------------------------------------------
CMLV            ------------------------------------------------------------
PeMV            ------------------------------------------------------------
RuSPaV          HE-KLDRSMWQMGLHGIKKSISMNGTSFTSDLCSCFSCHNFHKFKDLINNLRLALGAQGL
PChMoV          HDLQVDVDGSVVELIRGKVG-GKFGKGYQSDLCCCNMSWATDEDEPILETLRSLSFACGF
PVS             DAH---------------------------------------------------------
PVM             ------------------------------------------------------------
GVA             ------------------------------------------------------------
GVB             ------------------------------------------------------------
ChVA            ------------------------------------------------------------
ASGV            ------------------------------------------------------------
PVX             ------------------------------------------------------------
CymMV           ------------------------------------------------------------


CsFRLS1         ------------------------------------------------------------
CLBV            ------------------------------------------------------------
DMV             ------------------------------------------------------------
ACLSV           ------------------------------------------------------------
CMLV            ------------------------------------------------------------
PeMV            ------------------------------------------------------------
RuSPaV          GQCDRVVFATTGPGLSKVLEMPRSKKQSILVLEGALSIETDYGPKVLGSFEVFKGDFHIK
PChMoV          SNVDRVLISDVNSITTLSSLLEVEMGTLWCISGFILVKSEGGEVKIGEMMASKFMKMSII
PVS             ------------------------------------------------------------
PVM             ------------------------------------------------------------
GVA             ------------------------------------------------------------
GVB             ------------------------------------------------------------
ChVA            ------------------------------------------------------------
ASGV            ------------------------------------------------------------
PVX             ------------------------------------------------------------
CymMV           ------------------------------------------------------------


CsFRLS1         -----------------------KTDSELSIKFSNR------------------------
CLBV            --------------------------SPIAIREENY------------------------
DMV             --------------------------SPIAIREENY------------------------
ACLSV           -----------------------VHKRDFSFESKLR------------------------
CMLV            -----------------------VHERDFSFEEKRK------------------------
PeMV            -----------------------VHSRDFSFEEKRK------------------------
RuSPaV          KMEEGSIFVITYKAPIRSTGRLRVHSSECSFSGSKEV-------------------LLGC
PChMoV          GWSKDFLSLFFYKPRLGKGMQLRTHNEECELSDFTE-------------------QLFGC
PVS             -----------------------HTREGVAVHASGK------------------------
PVM             -----------------------TVSEQVTFESAQEVGPIEGKSDSGAPGVGVNLDLGGV
GVA             -----------------VDYEARKGDEDSEYEEDKA------------------------
GVB             -----------------------GSDKMSEYEETHR------------------------
ChVA            -----------------------QQSEEKPISEQEK------------------------
ASGV            -----------------------SESGEIKIEGSAE------------------------
PVX             -----------------------KHSREVKHQAAKK------------------------
CymMV           -----------------------SASTNISFGSFTP------------------------


CsFRLS1         ------------------------------------------------------------
CLBV            ------------------------------------------------------------
DMV             ------------------------------------------------------------
ACLSV           ------------------------------------------------------------
CMLV            ------------------------------------------------------------
PeMV            ------------------------------------------------------------
RuSPaV          QIEACADYDIDDFNTFSVPGDGNCFWHSVGFLLSTDGLALKAGIRSFVESERLVSPDLSA
PChMoV          TVRLSRKFNPDEFHVFDVPGDGNCFWHSVGPLIGVDGILLKGILR-----ERCGRNGITH
PVS             ------CPAAKKFHRVPNAGGGDCFWLAISHFTGVSVQDMKQGLQ-----QLDWESDAFS
PVM             VGSEYPANGAERYKRVSGPGDGCCCWHSFAYLVGMHHMELKRLCT-----SHVFENAALN
GVA             ------------------------------------------------------------
GVB             ------------------------------------------------------------
ChVA            ------------------------------------------------------------
ASGV            ------------------------------------------------------------
PVX             ------------------------------------------------------------
CymMV           ------------------------------------------------------------


CsFRLS1         ------------------------------------------------------------
CLBV            ------------------------------------------------------------
DMV             ------------------------------------------------------------
ACLSV           -----------------------------------------FIKG---------------
CMLV            -----------------------------------------FIEG---------------
PeMV            -----------------------------------------FVNG---------------
RuSPaV          PAISKQLEENAYAENEMIALFCIRHHVRPIVITPEYEVSWKFGEG-EWPLCGILCLKSNH
PChMoV          KELLRQMSGDTWAEREAVAFFCSEYSIQLHVLSISEGVTWIFKPA-KVVKSSTLKCQDNH
PVS             AELALQLKPQAWAEEEAIIATSKQYRYRIVVLSADKEQTVIYSPKCEAVQSMVLYHAGAH
PVM             VELEQCKASGAFVTHAAILATALRLRAEIRVHNAGTGRVHRFAPK-QKNMALDLWLESEH
GVA             -----------------------------------------------------ELDEGID
GVB             ------------------------------------------------------IGGKPG
ChVA            ------------------------------------------------------------
ASGV            ----------------------NDQPHEVSHTSMETEDGQGFEGSIPVDLINCFEPEEIK
PVX             --------------------------AKRLAEIQESMRAEGEAESNEMSGGMGAIPSNAE
CymMV           ------------------------------------------------------------


                      →Carlavirus endopeptidase;pfam05379
CsFRLS1         ------ENDCLINAVSSSLSIRKNKVVNALISIDKSFX---MKFFXADIGATIEDCVKIA
CLBV            ------KNTCLINAFSKAMKRSKQAIIAKLKTVNSPFW---SRYLSEGNGGSIEDCQSAC
DMV             ------KNTCLINAFSKAMKRSKQAIIAKLKTVNSPFW---SRYLSEGNGGSIEDCQSAC
ACLSV           ------KFDCLFVSVAEIIHKKPEEIMMFI-----PHI---MDRCVSNRGCSLDDAKAIC
```

```
CMLV            ------KYDCLFVAMAAMISKKPEDIMFKC-----LNT---LDRCVMNKGCDLTDLRSIC
PeMV            ------KFDCLFDAVSKLISKKSEEVMIRN-----PHI---LDRCIMNRGCDLDDLRSIC
RuSPaV          FQPCAPLNGCMITAIASALGRREVDVLNYLCRPSTNHI---FEELCQGGGLNMMYLAEAF
PChMoV          FMPCLPVNGCVVRAIASALGRREIDVLAVLGKPEHRDL---YEDACSGNGFCVYDLERLF
PVS             FEAALPRNDCVLVAVASVLRRRVEEVLSILGAQLGNEF---LQDVLKGEGIDRDKLAVVF
PVM             YEPQVLRNGCVIESVAQALGTRNADILAVVEERCCEEV---VESVQAGLGLNLHHVEIVL
GVA             YLQKNQGNMCSLKAFADHMQLSTPSVIAIVNGASPQTL-----REIEDGGYSLATLVNLS
GVB             CPREEQKESVSLQILADHMRVDLAICTSMVFAKDPR-----AREEVKRGGMTLGTFICVL
ChVA            --KETRKNDCFFKAVGETIGIPANSLIERILCSDSEDLKPVIEQLNLDHPISSKLLEVCC
ASGV            LPKRRRKNDCVFKAISAHLGIDSQDLLNFLVNEDISDE--LLDCIEEDKGLSHEMIEEVL
PVX             LPSTSGARQELTLPTTKPVPARWEDASFTDSSVEEEQVKLPGKEAVET--ATQQVIEGLP
CymMV           -------------------------------------------------EAEATPPPPMEKLP

                                                             peptidase←
CsFRLS1         QDLKLNLHIHCDDEIKIVD------CGSTLCEVRFSNNHY-------------SKLCSPE
CLBV            EALDVTVDLNVNGKCVVLG------KGALRISMALRNNHF-------------SVINAAQ
DMV             EALDVTVDLNVNGKYVVLG------KGALRISMALKNNHF-------------SVINAAQ
ACLSV           EKYEIKIECEGDCGLVECGT-----SGLSIGRMLLRGNHF-------------SVASVRR
CMLV            SGYEIKVECQGDCGLVEIGD-----IGLPLGKLILRGNHF-------------TLCSKRR
PeMV            NNYEIKVECQGDCGLLEVGS-----IGMPLGRFMLRGNHF-------------SLCSKRR
RuSPaV          EAFDICAKCDINGEIEVINP-----CGKISALFDITNEHI-------------RHVEKIG
PChMoV          EIFSIRARLDHQGSLICLNE-----DGKISAEFSLEKEHL-------------IHLKELS
PVS             KLFDICAHIHAEGEVFVINS-----EGRLPGTFNLSKDHI-------------EHCKSKP
PVM             QCFDIVGHCNLGDKEITLNA-----GGKMPFCFDISDEHM-------------SFCGRRK
GVA             KALDFPIAIHGERGYAETP------GSYRRLHLKITSGHV-------------EPFEGVT
GVB             KSLNLGAYIESERGCLYVN------GAYKELSCYAEDDHI-------------SEWSGPR
ChVA            KFLGYRVHIYYGDSIIKLNDD----INMHAIHIGGKPGHLFCINQERSKIPKDSQIKVPE
ASGV            ITKGLSMVYTSDFKEMAVLNRKYGVNGKMYCTIKGNHCEL--------------------
PVX             WKHWIPQLNAVGFKALEIQRDR---SGTMIMPITEMVSGL-------------EKEDFPE
CymMV           WDLWIPLLEQHGFKG---KSKLYKPTGELICPITEIKTV--------------PHCPFPD


CsFRLS1         ELPISLVSKNKEKGNLNINIGLVESLEK----------------RKHYNYLPFNAEGEFV
CLBV            LMERTFVSHLLEKGNVNVLEGFDAMLSGDVG-------------AAGVNKIQFAANFEFA
DMV             LMERTFVSHLLEKGNVNVLEGFDEMLSGDVG-------------AAGVNKIQFAANFEFA
ACLSV           SSMDSLANSSKE----------IKS-DGVLDHVTFNFHKRLKLVEPDLTNADIKVDSSRA
CMLV            SNLDSLANSSKD----------VSSLSGGIDYVMVNFIKRLRSIEPDLSRSEIKVDIKRG
PeMV            SNLDSLANSVKD----------VSVSSCGIDYVQMKFLDRLRAIEPDLNSTKVKVDIRRG
RuSPaV          NGPQSIKVDELR-----------KVKRSALDFLSMNGSKI--TYFPSFE---------RA
PChMoV          LKKFSPVISDLH-----------TVSKSALKLLMINGSEI--SYSPSMD---------RA
PVS             MGITKFTS--VHDASCEIKQETLSMLKAMCTLLPYNPCEL------------------RA
PVM             DPICKLVSGALH-----------GKMFAESALLDLENCGLKIDFEPNWN---------RA
GVA             SK--------------------------------GGFREAMLLGDGVGVGHFRVDKAKA
GVB             KE--------------------------------TTFSNALSMNPDIRVVKYEVCEQRA
ChVA            VGPQSFIGSIFSKTYGSGSSAPIHL--------------------------GQIDITKA
ASGV            SSKECFIRLLKEGGEAQMSNENLNA-------------------DSLFDLGRFVHNRDRA
PVX             GTPKELARELLAMNRSPATIPLDLL---------------------------------RA
CymMV           KVPDGCVLALKSIKRFATKMTMLSS---------------------------------RA


CsFRLS1         KMICSSFLQRSTGV-------CLGEVIDNGIKF----LKDSI------------------
CLBV            RILANSFLNMTTGI-------CLGKALDNGEKYFLHILKDRV------------------
DMV             RILANSFLNMTTGI-------CLGKALDNGEKYFLHILKDRV------------------
ACLSV           GKLLKSLMDGMTGI-------VSHNSTHEGWRM----IKGINSTSEMRSFMNMVRGQIEE
CMLV            GKLLKCLMEGLTGI-------VSHNSTHDGWRL----LKGVKNSADMRSLIGAMRGNVDG
PeMV            GKLLKCLMEGLTGI-------VSHNSTHDGWRL----LKGVKNSADMRSLVSAMRGSSAG
RuSPaV          EKLQGCLLGGLTGV-------ISDEKFSDAKPW----LSGISTT----------------
PChMoV          QTLANSLHAGTTGV-------MCAETYNSRRHIMDGLEANIAER----------------
PVS             KVLADSLNAGSTGV-------LCDELFNKVGNLL--------------------------
PVM             GMLADSMYQGATGV-------LGSALFNNKR-----------------------------
GVA             DRLAQSFYNGNTGV-------LLGKY--NKGKMHTGEIEEPK------------------
GVB             KKLVDSFQEGFTGV-------CLNKFQKQKSSFNLVNGKELI------------------
ChVA            LVLVSAFESMNLGVRVDRKAILEGQLISNG---FLAFLKRKN------------------
ASGV            VKLAKSMARGTTGL---------------LNEFDLEFCKNMV------------------
PVX             RDYGSDVKNKRIGA-------ITKTQAASWGEYLTGKIESLT------------------
CymMV           SAYTSDIKNSRTGKLLPAMNMPWKASLAYVTQHGDREIPGVV------------------
                      .     *
                                           → UvrD/REP helicase; cl14126
CsFRLS1         --------DLAEE-RNVICTNLHCLTGFAGSGKSR-VMQRWIRER--KRGNFCIVCPRVN
CLBV            ---------------KQIGIDVTMVCGFAGSGKSR-KLQSWLHSR--KKGNFCVVSPRTN
DMV             ---------------KQIGTDVTMVCGFAGSGKSR-KLQSWLHSR--KKGNFCVVSPRTN
ACLSV           -PKSDLFDKVQELNFMKV--KIYGIFGFAGSGKSH-AIQNLIQTEFKGSQGIMVICPRRF
CMLV            MEKNKLLNELEELNFQKV--PIYGIFGFAGSGKSH-AIQNLIAKEFRGSQGLMIVCPRKF
PeMV            IEKNKLMEELEELNFQQV--SIHGIFGFAGSGKSH-AIQDLISKEFRGSQGLMVVCPRKF
RuSPaV          --------DIKPR-------ELTVVLGTFGAGKSF-LYKSFMKRS--EGKFVTFVSPRRA
PChMoV          --------------------KLCTIIGTFGCGKST-LFKKFISKS--PGKAITFVSPRRS
PVS             --------EANEGRLRESVREVGCLLGTFGAGKSM-VFRKVLSSN--LGKSIIYVSPRKH
PVM             --------NMREKFVRNVSLSLHAIVGTFGSGKST-LFKNLLKYG--AGKSLDFVSPRRA
GVA             --------------------EVLTAFGFAGSGKSH-WCQTILKHC--SVEKVLVISPRKV
GVB             --------------------DVHLTLGFAGSGKSF-YPQCVLKNS--HYANSLVIVPRKA
ChVA            --------NEGHKVIKIQSLPVYPFIGFAGSGKSFGLTEKLINGD--CSQNFMFTAPRKK
ASGV            --------TLSELFPENFSSVVGLRLGFAGSGKTH-KVLQWINYT--PSVKRMFISPRRM
PVX             ----------------ERKVAACVIHGAGGSGKSH-AIQKALREI-GKGSDITVVLPTNE
CymMV           ------------------------IHGAGGCGKSY-AIQKWLRSC-SDPCAATVVCPTLG
                                          *  *.**:       :              *

CsFRLS1         LAEDWRIKL-------------QLSGKDQKK-----VCTFETFIKR--EKSGLDMIVLDE
CLBV            LAADWAFKL-------------ELEPNEQRK-----VSTFEKFIKT--DKSKLDLIVIDE
DMV             LAADWAFKL-------------ELEPNEQRK-----VSTFEKFIKT--DKSKLDLIVIDE
ACLSV           LAKDW-----------------SEKGVDEKD-----IKTFESALKS--DVKGKRLFILDE
CMLV            LAKDW-----------------SEKGVEEMD-----IRTFESALKS--DIKGKRVFILDE
PeMV            LAKDW-----------------SEKGVDDQD-----IKTFESALKS--DLKGKRVFILDE
RuSPaV          LANSIKNDLEMDDSCKVAKAGRSKK--EGWD-----VVTFEVFLRKVAGLKAGHCVIFDE
PChMoV          LADEIKCDIGLTGA--------NKKIGKGKDLKNVRVLTFELFLMGIASIKKGHTVIIDE
PVS             LADSFNELVK------------SIKQQEGQRVCKVSHFHVRESTTEVRAIRPDATIIIDE
```

```
PVM             LAEDFKRTVGMNE-----RGGRAKAGQENWR-----VTTLETFLARVEFLTEGQVVILDE
GVA             LRDDW-----------------VAKISKKHR-----VVTFEVAFMD---DYGCKDIVIDE
GVB             LCSDW-----------------SNKVHPDVK-----VVTFESAFRQ--QKKGYGLIVIDE
ChVA            IIGQIHERIDSRQYDD------KLKISRKKN-----FSTFENTLLS---LVNKPLVIMDE
ASGV            LADEVEPQLKGTACQ---------------------VHTWETALKK--IDGTFMEVFVDE
PVX             LRLDWSKKVPNTEPYM--------------------FKTYEKALIG----GTGSIVIFDD
CymMV           RRNDWLNKIGSYEQTN--------------------IKTFEKALIQ----PVNDVVIFDD
                   .                                    .              ...*:

CsFRLS1         MSLFPNGYLDWLIYD---------------------------MSKKEIFPEIICIFDPLQ
CLBV            LTLFPNGYLDLLVYE---------------------------LADVNRHCQIILLFDPLQ
DMV             LTLFPNGYLDLLVYE---------------------------LADVNRHCQIILLFDPLQ
ACLSV           ISLLPKGFTDLLMLK-------------------------MHMEGILKKSTIVCIGDPLQ
CMLV            VTLLPRGFTDLLLMK-------------------------IHMEGNLKSSTIICLGDPLQ
PeMV            VTLLPRGFTDLLMMK-------------------------IHMEGNLKKSTIVCLGDPLQ
RuSPaV          VQLFPPGYIDLCLLI-------------------------IRSD-----AFISLAGDPCQ
PChMoV          IQLFPPGYLDLILVC------------------------------TSSDINLVLAGDPCQ
PVS             IQLFPPGYLDLFSML------------------------------APAGVHMFLVGDPCQ
PVM             MQLYPPGYFDLVVSM------------------------------LKVDVRLFLVGDPAQ
GVA             IGLLPPGYIDLVIAA-------------------------------HQPRTLVLLGDPLQ
GVB             IGLLPPGYIDMVHGY-------------------------------FCYDSLLVLGDPLQ
ChVA            CSLNPPGFIDLVLIKSLDSIIRKSNKDFDHFFSSSVLSEGIIANVASPIACIAVTGDTLQ
ASGV            IGLYPPGYLTLLQMCAFRKIVKGQSENF---------LKGKLLELSKTCLNIRCFGDPLQ
PVX             YSKLPPGYIEALICF------------------------------YSKIKLVILTGDSRQ
CymMV           YTKLPPGYIETMVYH------------------------------HHNLDLIILTGDPMQ
                    * *:                                           :    *. *

CsFRLS1         ARYFNESDSHKLQ-F---DHEVDRI--VDKVHLNY----LYTSFRMEKTFFDGRFEKLHL
CLBV            ARYHNKMDESILTF----EHDVDRL--IGGQNIEY----IYSTHRMSRYF--NRFFDVPC
DMV             ARYHNKMDESILTF----EHDVDRL--IGGQNIEY----IYSTHRMSRYF--NRFFDVPC
ACLSV           AGYFCPKDDNYLSRE---GEIKRLF--KGGVNYKW------YSYRI-NKFIAKKL-AIET
CMLV            ASYFSQKDDSYLERE---PEVKRLF--KDGVEYKW------FSHRV-NKFTARQL-SITT
PeMV            ASYFSQKDESYLERE---PEVKRLF--PEGVEYKW------FSYRI-NRFVARQL-SITT
RuSPaV          STYDSQKDRAILGAE---QSDILRL--LEGKTYRY----NIESRRFVNPMFESRL-PCHF
PChMoV          SDYDSKDDRHLFANS---DSDIIHI--LNGKSYRY----NILSQRFRNPVFSSRL-PCSI
PVS             SDYDSEKDRSLFQAM---KSDINLL--LDDADYDF----NCRSRRFKDKLFDGRL-PCSM
PVM             SDYDSEKDRLVLGAM---EENMSVV--LGAREYNY----KVRSHRFLNCNFIGRL-PCEI
GVA             STYHSKRDNVVLEAS---QEDVFNR--VRG-KLPY----LCYSHRLPR---NCKLFEIEC
GVB             CEYHSKADHFFLGQ----EESVFKK--FKG-HCNY----LYKSHRLPR---NQKLFEIEC
ChVA            SSFYSESCGKLMQY----KNDIKTLCALSHTRLPY----LFGSKRFGYFTGFLKL-----
ASGV            LRYYSAEDTNLLDK----THDIDLM--IKTIKHKY----LFQGYRFGQWFQE----LVNM
PVX             SVYHETAEDASIRHLGPATEYF----------SKYCRYYLNATHR-----------NKKD
CymMV           SAYHETNRDAYISLIPDASASF----------SEYCEFNINATHR-----------NVAE
                  :        :                      :         *

CsFRLS1         PNE--GAQNKEIWMFEDPFNINSI------NYGLGVDVLLVESRLEKK------------
CLBV            FNQADRTEEQRLWIFDDVYSIPSICS----DRQEPCDVLLVESDLEKKAFSPI----INV
DMV             FNQADRTEEQRLWIFDDVYSIPSICS----DRQEPCDVLLVESDLEKKAFSPI----INV
ACLSV           MNDFIGIDEQSSIYKDMPSAHHFMEK-----KGNHIEVILVASMVEKELYSNY----GNV
CMLV            TNTFPGIDSQSQIYGDVQSAICSIQK-----TGIDVEIILVASMIEKELYCNL----GRT
PeMV            RNEFPGIDNQSQVYGDVPSAIHSIQK-----SGVKVEVILVASMIEKELYCNL----CQT
RuSPaV          KKGSMTAAFADYAIFH------NMHDFLLARSKGPLDAVLVSSFEEKKIVQSYFGMKQLT
PChMoV          CPKRLTMDEEEYTLWDSITQFELA-------GGKNFPVVLVSSFEEKKVVAAHLGLKMKC
PVS             GP--MEGESSKFTIIEGIENCKAIHS--------QAEVCLVSLFDEKKIVQTYFPSSCHC
PVM             NKDDCTIDEPHIMRMHLENLLDVAEEY--------KSVVLVSSFDEKMVVCAHLP-EAKV
GVA             ----MGAESEKRVVYR--------------SNRLKDEPTICATRAMKEEKGS------GW
GVB             ----DGAEGEGVTF----------------NKPRAKDLTLCASQRRKD-------SEKDV
ChVA            -GYYNQMESKAFTIDNMETLQKAI------GTSMDKFGVLVTSRADKSDFELDFP---NV
ASGV            PTRVDESKFSRKFFADISSV-----------KTEDYGLILVAKREDKGVFAGRVP----V
PVX             LANMLGVYSERTGVTEISMSAEFL----------EGIPTLVPSDEKRRLYMGT--GRNDT
CymMV           LACLLGVYSERQGKLAVSFSTAPLSK--------GKVPILVPSRMKQEAFADV---GNRC
                                                       :      :

                                                   helicase←
CsFRLS1         --------------SIRFINQ---------------------------------------
CLBV            MTFGESQGLTFNH-VCILLSESSAASNEFRWMVALTRARTRFSLCSTFLGGIEEFKVKR-
DMV             MTFGESQGLTFNH-VCILLSESSAASNEFRWMVALTRARTRFSLCSTFLGGIEEFKVKR-
ACLSV           MTFGESQGLTFNC-GVIVLSEEAKLCSDAHIMVAITRFRRGFCFALGSKGSKE--DYMRS
CMLV            ITFGESQGLTFGV-GVVVLSEETKLCSDAHIMVAITRFNKGYSFALGSKGTKD--EYMRG
PeMV            MTFGESQGLTFGV-GIIVLSEEAKLCSDAHIMVAVTRFNRGFSYALGSKGTKE--EYMRG
RuSPaV          LTFGESTGLNFKN-GGILISHDSFHTDDRRWLTALSRFSHNLDLVNITGLRVE--SFLSH
PChMoV          LTFGESTGLNFQK-GAIFVSYESSLTSERRWLTALSRFSHEIHFIN--GLSIEWSNVMSL
PVS             FTFGESTGMTYKS-GVILITDTSQYTSERRWLTALSRFSHSIAFVNATGGNIQ--LVTRL
PVM             LTFGESTGLTFMH-GTIYISAVSERTNERRWITALRRFRFNLCFVNCSGMDYQ--QLAGR
GVA             YTVSETQGLSFKS-CLIYLDEHWAKKEDEDVMVALTRSRGEIGIHVTPALKKK---LITN
GVB             NTVGESQGLSANR-VNILLDKDWSLVNDETVIVAFTRARKEINIIGDASLVNN---LKRS
ChVA            CTINEAQGSTFNS-VILIVTRDFFSNPIESIIVAITRHQKNLLIYFPAAIQGEMDFLSRR
ASGV            ATVSESQGMTISKRVLICLDQNLFAGGANAAIVAITRSKVGFDFILKGNSLKEVQRMA--
PVX             FTYAGCQGLTKPK-VQIVLDHNTQVCSANVMYTALSRATDRIHFVNT-------------
CymMV           MTYAGCQGLTAPK-IQILIDNHTAFCSEQTLYTCLSRAVDQIHFINT-------------
                                  :

CsFRLS1         ---------------------------------------------MYDVWXVLSPGPX--
CLBV            ----KESLITSILQGEKITFNRLNLMLKCNLIRRE---KENGCRDEVDREERLEGDPFLK
DMV             ----KESLITSILQGEKITFNRLNLMLKCNLIRRE---KENGCRDEVDREERLEGDPFLK
ACLSV           M---KSGLLQRICSGVGASKEFILGSSSVNLILSEKDIAKGAGIDEMDREARLEGDVWLK
CMLV            M---KNGLLSRLTSNSGASKDFIMSSSSVKLNLSEKLIKTGAGIDEMDREERLSGDPWLK
PeMV            I---KNGLLSRLCSSIGASKDFILSSSPIPLNFSEKMIQKGAGLDEMDREERLSGDPWLK
RuSPaV          F---AGKPLYHFLTAK-SGENVIRDLLPGEPNFFSGF-NVSIGKNEGVREEKLCGDPWLK
PChMoV          F---HGKALNKFFSKR-ASHDDVVDLLPGKPEFIEGF-QVNIGRDEEVREPKLSGDPWLK
PVS             Y---QNRVLGRFLLKT-AKIDDLKMLLPGRPRFKEGFGGERIGADEGKREFKLEGDPWLK
PVM             Y---KGRVRSKFLCKT-AIPDDLNSMLPGQALFKSEY-PRLIGKDEGVREEKLAGDPWLK
GVA             A---KSTLLKKVLKGETYRRSEIVAMVRKHIPETTVLFEESRLAETVDYEARLAGDPYLK
GVB             A---KSTVLKKILGGERVTEGLILSLIRKKLPDVIWLRRRCCSGQSDEMEEKLSGDPYLK
ChVA            FPIHSNVVLKNF--------SVLDNLIKDKLNPFQLIQEDPFGHD---FEVKLEGDPFLK
```

```
ASGV            ----QKTIWQFIIEGKSIPMERIVNMNPG-----ASFYESPLDVGNSSIQDKASNDLFIM
PVX             ------------------------------------------SANSSAFWEKLDSTPYLK
CymMV           ------------------------------------------GPNSQAFWTKLESTPYLK


CsFRLS1         ------------------------------------------------------------
CLBV            PFIFLGQRVEKDE--DEVEEV-KIREPTCQTHLYITEPNFGLCYNFDFIREKEQREYRE-
DMV             PFIFLGQRIEKDE--DEVEEV-KIREPTCQTHLYITEPNFGLCYNFDFIREKEQREYRE-
ACLSV           SMIYLGKRYHMVE--PLGQVI-KLTDDAIKCHIPVCSSQTLGPE-LDNIQAREYREFKG-
CMLV            SQIFLGKRFHLRE--PLGQVV-NLEDSAIKCHLPICNNQTLYVE-LSKMRAREDREFKG-
PeMV            TQIFLGKRFHLRE--PLGQVC-QIEDSAIKCHVPLSNQQTLYVE-LSRMKAREDREFKG-
RuSPaV          VMLFLGQDEDCEV--EEMESE-CSNEEWFKTHIPLSNLESTRARWVGKMALKEYREVRC-
PChMoV          TAIFLGQQPDIEE--EEMAEE-IIQEDWFKTHIPVFPLEAVRARWVHKILAKEAREFRV-
PVS             TMLDLLQKEDQEE--VEEAVV-ELGEEWFRTHLPQCELEGVRARWVEKILAKEVREKRM-
PVM             TMINLYQAPEVEI--AEEPEV-VMQEEWFRTHLPRDELESVRAQWVHKILAKEYREVRM-
GVA             SLLALYDEIEMED--IEIEEP-VTLEPT-KTHLALSTKMNELA--PFDLKAKEHREQHT-
GVB             GLLCLIDETEEEE--VEIPEP-ELLEPQ-KTHLPLCVGENELS--ISDLRSKESREVST-
ChVA            SELSLVNEIKLQQ--IEENSI--ESKENLKTHLPISYSGLWNLE-ISEMRAREDREFKKF
ASGV            PFINLAEEEVDPE--EVVGDV-IQPVEWFKCHVPVFDTDPTLAEIFDKVAAKEKREFQS-
PVX             TFLSVVREQALKE--YEPAEAEPIREPEPQTHMCVENEESVLEEYKEELLEKFDREIHSE
CymMV           AFLDNYREEQTERLTSTAPEREVREPAPPKTHIPVENTSGLRISALD-LPEKHSREIFNK


CsFRLS1         ------------------------------AMY---------------------------
CLBV            DMLVTNQFCDSYDKVHINGKRETPGPLRFKAIYPKHSADDDMTFWMAVRKRLVFREEEEN
DMV             DMLVTNQFCDSYDKVHINGKRETPGPLRFKAIYPKHSADDDMTFWMAVRKRLIFREEEEN
ACLSV           KNGWSNQFREEAGP-NWKFPYKVNQAMSYEAVYPRHKMDDDLTFLAAIKKRLRFDNVANN
CMLV            KDGWSKQFREEAGP-NWKSPYKVAQPMNFEAIYPRHRMDDDITFYAAIKKRLRFDNVANN
PeMV            RDGWSNQFREEAGP-NWKFPYKVAQPMNFEAIYPRHRMDDDITFYAAIKKRLRFDSVANN
RuSPaV          GYEMTQQFFDEHRG-GTG-EQLSNACERFESIYPRHKGNDSITFLMAVRKRLKFSKPQVE
PChMoV          GHEVTEQFIDEHSK-NPG-KQITNAAERYETIYPRHKGTDSVTFLMAVKKRLSFSQPSIE
PVS             GLLVSEQFTDEHSK-QLG-KQITNAAERFETIYPRHRAADTVTFIMAVRKRLRFSDPIRD
PVM             GDMVSEQFTHDHTK-QLGAKQLTNAAERFETIYPRHRASDTVTFLMAVKKRLSFSNPGKE
GVA             EAGRTEQID------ENGYQGEVGDPMTHKALYLRHTSDDTATFMMSVKKRLRFRNYEAN
GVB             EAGRTDQID------ELGYKGEPENPMNHKALYLYHQNSDVATFFLSIRKRLRFRDPEKN
ChVA            GVGWSKQFKDEP---NQKDQVEDNCAMLPEAVFPRHFANDDLTFWSAVKKRLVFKNPLSN
ASGV            VLGLSNQFLDM----EKNGCKIDILPFARQNVFPHHQASDDVTFWAGVQKRIRKSNWRRE
PVX             SHGHSNCVQTEDTTIQ---------------LFSHQQAKDETLLWATIDARLKTSNQETN
CymMV           AHGFSNAIQGEGVA----------------PMFQHQQAKDETLFKATIDARLSITHPDEN
                               ::

 RNA dependent RNA polymerase; pfam00978→
CsFRLS1         -----------------YDKHFFLGHGVPPLRGIQFLTLISDGMSILQE-----------
CLBV            YQRLSRAHLVGGLLYTNFKKKM-------GLEFTFDQGLLEESINAFEKKKLEKSCGTIK
DMV             YQRLSRAHLVGGLLYTNFKKKM-------GLEFTFDQGLLEESINAFEKKKLEKSCGTIK
ACLSV           YAKFKAAESRGKYLTKIFLKHV-------PIKCGRDQRLLDQCRQEFEETKLSKSAATIG
CMLV            YAKFKQSQSRGQYLLKVFLEHV-------NLKPSRNQALLNQCRQEFEETKLNKSAATIG
PeMV            YTKFKQSQSRGQYLLQVFLKHV-------DLKPSRNQALLNQCKQEFEETKLSKSAATIG
RuSPaV          AAKLRRAKPYGKFLLDSFLSKI-------PLKASHNSIMFHEAVQEFEAKKASKSAATIE
PChMoV          SAKLRRAMPFGKFLLQTFLKYI-------PLCGKHDSDLMSKAVNDFEEKKLSKSAATIE
PVS             AAKLRAAEMYGPFLLKEFLKHV-------PLKPMHDTRMMAEAKFDFEEKKTQKSAATIE
PVM             KGNLFHAASYGKALLSEFLKRV-------PLKPNHNVRFMEEALWNFEEKKLSKSAATIE
GVA             RRKYKTCHGIGHQMFSVFKDTY-------QLKEIDSLPELERCEMEFMKKRIEKSTGLIE
GVB             RRKFNKCKGFGAQMFGVMKSVY-------NLKQPDHLPLLERAEQDFMKKRIAKSAKLIE
ChVA            AHDFEKAKPFGKELLNIFLRKV-------PLMPNFDQRMYDESVSEFEEKKISKNAAMIG
ASGV            KSKFEEFESQGKELLQEFISML-------PFEFKVNIKEIEDGEKSFLEKRKLKSEKMWA
PVX             FREFLSKKDIGDVLFLNYQKAMGLPKERIPFSQ----EVWEACAHEVQSKYLSKSKCNLI
CymMV           KREFAMKKDTGDVLFVNYKAIMNLPHEPVPFEP----RLWNICKAEVQNTYLAKPIANLI
                              :                     .

CsFRLS1         ----------------------------------------YTTQCIIGRIGLYFK---R---
CLBV            SHSIRSDIDWALNDVFLFMKSQLCTKYEKQ-FVDAKAGQTLACFQHLILVQFAPWCRYLE
DMV             SHSIRSDIDWALNDVFLFMKSQLCTKYEKQ-FVDAKAGQTLACFQHLILVQFAPWCRYLE
ACLSV           AHSQRSDSDWPLDKIFLFMKSQLCTKFEKR-FTEAKAGQTLACFPHKILVEFSPWCRYTE
CMLV            AHSQRSDPDWPLDRIFLFMKSQLCTKFEKR-FEDAKAGQTLACFQHRILVEFSPWCRYVE
PeMV            AHAQRSDPDWPLDRIFLFMKSQLCTKFEKR-FEDAKAGQTLACFQHKILVEFSPWCRYVE
RuSPaV          NHAGRSCRDWLLDVALIFMKSQHCTKFDNR-LRVAKAGQTLACFQHAVLVRFAPYMRYIE
PChMoV          NHSGRSSRDWPVDKALIFMKSQLCTKFDNR-FRSAKAGQTLACFQHSVLCRFAPYMRYIE
PVS             NHRNRSCRDWLADMGMVFSKSQLCTKFDNR-FRDAKAAQTIVCFQHSVLCRFAPYMRYIE
PVM             NHSGRSCRDWPTDVAQIFSKSQLCTKFDNR-FRVAKAAQSIVCFQHAVLCRFAPYMRYIE
GVA             KHAGRSDPDWPSNYLKIFLKQQTCTKMEKR-GVDAKAGQTIACFAHSVLCRFGPILRQTE
GVB             KHSYRSEPDWPSNYLKIFLKQQRCTKMEKR-KVDAKAGQTIACFCHAVLCRFGPLLRQTE
ChVA            AHHDRSTTDWPTNEIFLFIKSQLCTKKEKM-FCDAKAGQTLACFSHLILCKFAPLNRYIE
ASGV            NHSERSDIDWKLDHAFLFMKSQYCTKEGKM-FTEAKAGQTLACFQHIVLFRFGPMLRAIE
PVX             NGTVRQSPDFDENKIMVFLKSQWVTKVEKLGLPKIKPGQTIAAFYQQTVMLFGTMARYMR
CymMV           NGTLRQSPDFPANKIALFLKSQWVKKIEKIGAIPVKPGQTIASFMQETVMLYGTMARYLR
                                                       :   .:       :    *

CsFRLS1         --------------------------------SGHICDPQNW-----PLDHSLLQF----
CLBV            TQIRNQLPEEIYIHSNKNFDDLNAWV-KKFF-QRDICVESDYEAFDASQDEYILSFEIHL
DMV             TQIRNQLPEEIYIHSNKNFDDLNAWV-KKFF-QRDICVESDYEAFDASQDEYILSFEIHL
ACLSV           KVLTANLPDNYYIHQRKNFSELEDFA-RRFS-NGSICVESDYTAFDVSQDHTILAFEVEL
CMLV            KILTACTPDNFYIHQRKNFSELENFA-KRFS-DGSICVESDYTAFDVSQDHTILAFEVEL
PeMV            KILTSCLPENFYIHQRKNFSELEAFA-KRYS-DGSVCVESDYTAFDVSQDHTILAFEVEL
RuSPaV          KKLMQALKPNFYIHSGKGLDELNEWV-RTRG-FTGICTESDYEAFDASQDHFILAFELQI
PChMoV          AKLFNALPDRFYIHSGKNIDDLGNWVKKQN--FSGECTESDYEAFDASQDHFILAFEIEI
PVS             KKLNEVLPARFYIHQRKNFSELEAFA-KRYS-DGSVCVESDYTAFDVSQDQYIVAFELAL
PVM             MKVHEVLPKNYYIHSGKGLEELDAWVKKGK--FDRICTESDYEAFDASQDEFIMAFELEL
GVA             KALRELLPEKLMIYSQKKYMDLDKWA-KTWV-ESMMGTDSDYEAFDRSQDEKVLDLEVEV
GVB             KALRDQLGPNVMIYSQKNYTDLDKWC-KGFV-HTLDGTDSDYEAFDRSQDEKVLDFEVNV
ChVA            KKVTQSLPGNFYIHQKKNFDELERWV-KSYN-FSGVCTESDYKAYDASQDSCTLAFEYKL
ASGV            SAFLRSCGDSYYIHSGKNFFCLDSFVTKNASVFDGFSIESDYTAFDSSQDHVILAFEMAL
PVX             WFRQAFQPKEVFINCETTPEDMSAWALNNWN-FSRPSLANDYTAFDQSQDGAMLQFEVLK
CymMV           KMRQRYQPAHIFINCEKTPEDFNKFVLEHWS-HKQVAHTNDFTAFDQSQDAAMLQFEVIK
                                                       .::     . *   : :
```

```
CsFRLS1         -----------------------VEGSLT---FEC----PIPLIV---------------
CLBV            MKDAHFPQKIIDAYIDLKCKLGCKLGHFSIMRFTGEFCTFLFNTLANMAFTMCRYEW-RR
DMV             MKDAHFPQKIIDAYIDLKCKLGCKLGHFSIMRFTGEFCTFLFNTLANMAFTMCRYEW-RR
ACLSV           LRHFGWDDRVLQSYIKMKCTLGCRLGGFAIMRFTGEFSTFLFNTLANMVFTFCRYEV-PD
CMLV            LRFIGWDEKILNSYIKMKCTLGCRLGGFAIMRFTGEFSTFLFNTLANMAFTFCRYEV-KK
PeMV            LRYIGWDEAVLNSYIKMKCTLGCRLGGFAIMRFTGEFSTFLFNTLANMAFTFCRYEV-KK
RuSPaV          MKFLGLPEDLILDYEFIKIHLGSKLGSFSIMRFTGEASTFLFNTMANMLFTFLRYEL-TG
PChMoV          MRHLGLPEGLINDYIFIKCNLGSKLGSFAIMRFTGEASTFLFNTMANMLFTFLRYEL-SG
PVS             MKYLGLPNDLIEDYKYIKTHLGSKLGNFAIMRFSGEASTFLFNTMANMLFTFLRYKL-KG
PVM             MKYLRLPSDLIEDYKFIKTSLGSKLGNFAIMRFSGEASTFLFNTLANMLFTFMRYNI-RG
GVA             LRFFLWPEDLIREYEELKLMMGCALGDLAVMRFSGEFGTFFFNTVCNMVFSCMRYHI-DR
GVB             LKFFLWPEEMIEEYVTLKLMMGCSMGSLAVMRFSGEFGTFFFNTICNMGFTCLKYSI-RA
ChVA            LRYLAFSNSLIEDYLYLKMHLNCKLGNLAIIRFTGEFCTFLFNTLTNMLFTFMKYDV-RK
ASGV            LQYLGVSKEFQLDYLRLKLTLGCRLGSLAIMRFTGEFCTFLFNTFANMLFTQLKYKIDPR
PVX             AKHHCIPEEIIQAYIDIKTNAHIFLGTLSIMRLTGEGPTFDANTECNIAYTHTKFDI-PA
CymMV           ARYFNIPEDVIEGYIQIKLTAEIFLGTLSIMRLSGEGPTFDANTECSIAYNATRYHI-NE
                                         * ::   :

                                                                RdRp ←
CsFRLS1         ---------------------------LLQQVSQRA---------------CSFRES-F-
CLBV            GQPIAFAGDDMCALNNLAVCHDFDD--LFELISLKAK---V--ERTETPMFCGWRLTPYG
DMV             GQPIAFAGDDMCALNNLAVCHDFDD--LFELISLKAK---V--ERTETPMFCGWRLTPYG
ACLSV           GTPICFAGDDMCALRNLREIDTHEF--ILSKLSLKAK---V--NRTKVPMFCGWRLCCDG
CMLV            GTPICFAGDDMCALRNLREVSTHEH--ILDKLSLKAK---V--NRTTVPMFCGWRLCQDG
PeMV            GTPICFAGDDMCALRNLREIASHEH--ILNKLSLKAK---V--NRTNVPMFCGWRLCQDG
RuSPaV          SESIAFAGDDMCANRRLRLKTEHEG--FLNMICLKAK---V--QFVSNPTFCGWCLFKEG
PChMoV          HESISFAGDDMCANRRLRVRSTYKS--FLEKIRLKAK---V--QFTNFPTFCGWGLCPEG
PVS             DERICFAGDDMCANRALFIKDTHEG--FLKKLKLKAK---V--DRTNRPSFCGWSLCSDG
PVM             DEFICFAGDDMCASRRLQPTKKFAH--FLDKLKLKAKVQFV--QFVNKPTFCGWHLCPDG
GVA             NTPMCFAGDDMYSPGILRVKKDYEA--TLDQLTLKAK---V--HISEEPLFCGWRMSPFG
GVB             DTPICYAGDDMYAPGILVTKSEFKH--ILDELQLKAK---V--NYTRSPLFCGWRMSPYG
ChVA            THAICFAGDDMCANVRLPENHEYSS--LLKKFSLKAK---V--DFTRSPTFCGWNLSRYG
ASGV            RHRILFAGDDMCSLSSLKRRRGERATRLMKSFSLTAV-----EEVRKFPMFCGWYLSPYG
PVX             GTAQVYAGDDSALDCVPEVKHSFHR--LEDKLLLKSKPVITQQKKGSWPEFCGWLITPKG
CymMV           DVTQVYAGDDMAMDHVCPEKKSFKA--LEKKLKLTSKPLYPKQKPGDWADFCGWTITPYG
                                             . .   :               *.:

CsFRLS1         ------------------------------------------------------HESLV-
CLBV            IVKEPELVYNRFQVAIEEGKVLECLENYAIEVSYAYSLSERLYEVLKSERQVQYHQAVVR
DMV             IVKEPELVYNRFQVAIEEGKVLECLENYAIEVSYAYSLSERLYEVLKSERQVQYHQAVVR
ACLSV           LIKEPCLIYERLQVAIENGRLMDVIDSYFLEFSFAYKLGERLYSHLEIE-QLNYHQVLTR
CMLV            LIKEPCLIYERLCVAIENGRLLDVIDSYYLEFSFAYKLGERLFQYLEIE-QLNYHQVLAR
PeMV            LIKEPCLIYERLNVAIENGRLLDVIDSYYLEFSFAYKLGERLFQYLEIE-QLNYHQVLTR
RuSPaV          IFKKPQLIWERICIAREMGNLENCIDNYAIEVSYAYRLGELAIEMMTEE-EVEAHYNCVR
PChMoV          VFKKPDLVLERLQIAVETNNLQNCIDNYAIEVSYAYSMGESLSKYLSEE-EMDAHYNCVR
PVS             IYKKPQLVFERLCIAKETANLANCIDNYAIEVSYAYKLGERIKERMSEE-ELDAFYNCVR
PVM             IYKKPQLVLERMCIAKEMNNLSNCIDNYAIEVAYAYKLGEKAVNRMDEE-EVAAFYNCVR
GVA             IIKEPNLILDRWKIALRSGNLSLCLVNYAIEASFGYRLSEHLYDVNI---DVDAQQELVR
GVB             IVKDPNLLLDRWKIAERDGSLKNCMVNYALEAIYGYRLGEHLFDLNV---DIDAQQDLIR
ChVA            IVKKPELIAARLAVARQKGEVNLVLDSYFLEHLYAYNKGDHLFEILSEK-ELEHHYNLTR
ASGV            IIKSPKLLWARIKMMSERQLLKECVDNYLFEAIFAYRLGERLYTILKEE-DFEYHYLVIR
PVX             VMKDPIKLHVSLKLAEAKGELKKCQDSYEIDLSYAYDHKDSLHDLFDEK-QCQAHTLTCR
CymMV           IIENPKKLDACLQLHTQLGDADKVARSYALDAKYAYDLGDRIHEILNAD-EMTSHFNVIR

CsFRLS1         -IIG--------------------------------------------------------
CLBV            FIVTHIDKLKTKVRDLFLEQSSDEDI----------------------------------
DMV             FIVTHIDKLKTKVRDLFLEQSSDEDI----------------------------------
ACLSV           FFIRNKHLLRGDSRHNISELEWLSDEDGDNDKGSQIEDRRRGYSNCWGEKLQNLF-----
CMLV            FFVKNSHLLRGSAREGISELSWLSDGDDNDDESSQVKNRRRGYSNIWGEKLQSLF-----
PeMV            FFVKNSHLLRGGARKSVFQNSVGFHEDGSDDEGSQIKNRRRGYSNFWSEKLQN-------
RuSPaV          FLVRNKHKMRCSISGLFEAI----------------------------------------
PChMoV          FIVKHSHLLKCSVSDLFRSI----------------------------------------
PVS             VIIKHKHLLKSEIRCVYEDV----------------------------------------
PVM             IIVRNKHLIRSDVKQVFEVL----------------------------------------
GVA             EIVIKKHLLPKKISDLFSEDECERHSDGDEDFLSNDVARLYRIE----------------
GVB             RIVLIKHLLPQSLQRFYSDEERECFSDGEEFNLKVRNEGGLDESEFAEE-----------
ChVA            FFVKNSKLLKGESKKKFMETKEIEGGLFGECDFGNDSIFKDYINRVKNKVEIDLLNERIL....
ASGV            FFVRNSKLLTGLSKSLIFEI-----------------------------------GEGI-....
PVX             TLIK--------------------------------------------------------....
CymMV           QLHK--------------------------------------------------------....
                 :
```
