# Supplementary material for: Widespread Endogenization of Genome Sequences of Non-Retroviral RNA Viruses into Plant Genomes
Source: PLoS Pathog. 2011 Jul 14;7(7):e1002146. doi: 10.1371/journal.ppat.1002146 (PMC3136472; doi:10.1371/journal.ppat.1002146)
Supplement: Table S1 — Virus gene sequences used as query sequences in the search for non-retroviral integrated RNA viruses. (DOC) [file ppat.1002146.s007.doc]

**Table S1. Virus gene sequences used as query sequences in the search for non-retroviral integrated RNA viruses.**

| **Order** | **Family** | **Genus** | | **Species** | | **Accession or reference numbers** | | |
| --- | --- | --- | --- | --- | --- | --- | --- | --- |
| **dsRNA viruses** |  |  | |  | |  | | |
|  | Partitiviridae | Partitivirus | | Rosellinia necatrix partitivirus 1 | | [dsRNA1:NC_007537, dsRNA2:NC_007538] | | |
|  |  | Unassigned | | Rosellinia necatrix partitivirus 2 | | [dsRNA1:AB569997, dsRNA2:AB569998] | | |
|  |  | Unassigned | | Rahphanus sativus cryptic virus 1 | | [dsRNA1:NC_008191, dsRNA2:NC_008190] | | |
|  |  | Unassigned | | Rahphanus sativus cryptic virus 2 | | [dsRNA1: NC_010343, dsRNA2:NC010344, dsRNA2:NC010345] | | |
|  |  | Unassigned | | Rahphanus sativus cryptic virus 3 | | [dsRNA1:NC_011705, dsRNA2:NC_011706] | | |
|  | Reoviridae | Phytoreovirus | | Rice dwarf virus | | [dsRNA1-3:NC_003772-NC_003774, dsRNA4-12: NC_003760-8] | | |
|  |  | Fijivirus | | Fiji disease virus | | [dsRNA1-10: NC_007154-NC_007163] | | |
|  | Endornaviridae | Endornavirus | | Vicia faba endornavirus | | [NC_007648] | | |
| **ssRNA viruses** |  |  | |  | |  | | |
| *ssRNA negative-strand viruses, no DNA stage* | | |  | |  | |  |  |
|  | Bunyaviridae | Tospovirus | | Tomato spotted wilt virus | | [RNA-L:NC_002052, RNA-M:NC_002050, RNA-S:NC_002051] | | |
|  |  | Unclassified | | Fig mosaic virus | | [RNA1:AM941711,RNA2:FM864225, RNA3:FM991954, RNA4: FM992851] | | |
|  | Unassigned | Tenuivirus | | Rice stripe virus | | [RNA1:NC_003755, RNA2: NC_003754, RNA3:NC_003776, RNA4: NC_003753 ] | | |
|  | Ophioviridae | Ophiovirus | | Citrus psorosis virus | | [RNA1:NC_006314, RNA2:NC_006315, RNA3:NC_006316] | | |
|  | Unassigned | Varicosavirus | | Lettuce big-vein associated virus | | [RNA1:NC_011568, RNA2:NC_011558] | | |
|  | Unassigned | (Dichorhabdovirus) | | Orchid fleck virus | | [RNA1: NC_009608, RNA2: NC_009609] | | |
| Mononegavirales | Rhabdoviridae | Cytorhabdovirus | | Lettuce necrotic yellows virus | | [NC_007642] | | |
|  |  | Nucleorhabdovirus | | Potato yellow dwarf virus | | [GU734660] | | |
| *ssRNA positive-strand viruses, no DNA stage* | | | | |  | | | |
|  | Unassigned | Benyvirus | | Beet necrotic yellow vein virus | | [RNA1: NC_003514, RNA2:NC_003515] | | |
|  | Bromoviridae | Cucumovirus | | Cucumber mosaic virus | | [RNA2:NC_002035, RNA3:NC_001440] | | |
|  | Unassigned | Cilevirus | | Citrus leprosis virus C | | [RNA1:NC_008169, RNA2:NC_008170] | | |
|  | Closteroviridae | Closterovirus | | Citrus tristeza virus | | [NC_001661] | | |
|  | Luteoviridae | Luteovirus | | Barley yellow dwarf virus | | [D10206] | | |
|  | Potyviridae | Potyvirus | | Potato virus Y | | [NC_001616] | | |
|  | Unassigned | Sobemovirus | | Southern bean mosaic virus | | [NC_004060] | | |
|  | Tombusviridae | Tombusvirus | | Tomato bushy stunt virus | | [NC_001554] | | |
|  | Virgaviridae | Furovirus | | Soil-borne wheat mosaic virus | | [RNA1:NC_002041, RNA2:NC_002042] | | |
|  |  | Hordeivirus | | Barley stripe mosaic virus | | [RNA2:NC_003481, RNA3:NC_003478] | | |
|  |  | Tobamovirus | | Tobacco mosaic virus | | [NC_001367] | | |
|  | Unassigned | Umbravirus | | Groundnut rosette virus | | [NC_003603 ] | | |
| Tymovirales | Alphaflexiviridae | Potexvirus | | Potato virus X | | [NC_011620 ] | | |
|  | Betaflexiviridae | Carlavirus | | Potato virus M | | [NC_001361] | | |
|  | Tymoviridae | Tymovirus | | Turnip yellow mosaic virus | | [NC_004063 ] | | |
| Picornavirales | Secoviridae | Comovirus | | Cowpea mosaic virus | | [RNA1: NC_003549, RNA2: NC_003549] | | |
