# Supplementary material for: Widespread Endogenization of Genome Sequences of Non-Retroviral RNA Viruses into Plant Genomes
Source: PLoS Pathog. 2011 Jul 14;7(7):e1002146. doi: 10.1371/journal.ppat.1002146 (PMC3136472; doi:10.1371/journal.ppat.1002146)
Supplement: Table S2 — Amino acid sequence identities among selected partitivirus CPs and plant Partitivirus CP-like sequences (PCLSs). (DOC) [file ppat.1002146.s008.doc]

**Table S2. Amino acid sequence identities among selected partitivirus CPs and plant Partitivirus CP gene like sequences (PCLSs).**

| **Seqence name** | **Plant or virus name**  **(locus or accession)** | **Sequence**  **Size (aa)** | **RnPV2CP** | **AtPCLS1** | **CrPCLS1** | **OpPCLS1** | **MgPCLS1** | **RSCV1 CP** | **BrPCLS5** | **SpPCLS5** | **NtPCLS5-1** | **CaCV1 CP** |
| --- | --- | --- | --- | --- | --- | --- | --- | --- | --- | --- | --- | --- |
| **AtPCLS1** | *A. thaliana* Col-0 (At3g18485) | 386 | 32.8%  /403 aa |  |  |  |  |  |  |  |  |  |
| **CrPCLS1** | *C. rubella (*AB576171) | 417 | 34.2%  /406 aa | **71.3%**  **/404 aa^b^** |  |  |  |  |  |  |  |  |
| **OpPCLS1** | *O. pumila* (AB576174) | 227 | 37.3%  /217 aa | **70.8%**  **/185 aa** | **81.5%**  **/227 aa** |  |  |  |  |  |  |  |
| **MgPCLS1** | *M. guttatus* (mg022475m) | 452 | 31.6%  /437 aa | **49.4%**  **/156 aa** | **42.9%**  **/422 aa** | **43.1%**  **/232 aa** |  |  |  |  |  |  |
| **RSCV1 CP** | RSCV1  (DQ181927) | 502 | 21.8%  /358 aa | 30.4%  /115 aa | 25.9%  /316 aa | 28.1%  /114 aa | 22.7%  /304 aa |  |  |  |  |  |
| **BrPCLS5** | *B. rapa*  (Bra020160) | 427 | 23.2%  /353 aa | 21.6%  /213 aa | 21.2%  /415 aa | 22.1%  /199 aa | 21.4%  /285 aa | **56.2%**  **/416 aa** |  |  |  |  |
| **SpPCLS5** | *S. phureja*  (unnamed**^a^**) | 283 | 25.2%  /210 aa | 22.7%  /176 aa | 23.3%  /163aa | 20.5%  /205 aa | 27.3%  /150 aa | **45.8%**  **/275 aa** | **60.8%**  **/209 aa** |  |  |  |
| **NtPCLS5-1** | *N. tabacum*  (GSS Contig-1) | 221 | 25.4%  /142 aa | 42.9%  /84 aa**^c^** | 31.7%  /123 aa | 34.1%  /123 aa | 28.9%  /128 aa | **45.0%**  **/189 aa** | **59.3%**  **/140 aa** | **62.4%**  **/189 aa** |  |  |
| **CaCV1 CP** | CaCV  (FJ550605) | 490 | 18.9%  /169 aa | 17.3% /191 aa | 21.4%  /173 aa | 19.8%  /162 aa | 25.6%  /78 aa | 19.0%  /494 aa | 20.8%  /409 aa | 26.0% /181 aa | 25.8%  /155 aa |  |
| **BrPCLS4** | *B. rapa*  (Bra021820) | 293 | 24.7%  /158 aa | 23.5% /102 aa | 17.2%  /215 aa | 16.9% /  /231 aa | 22.6%  /195 aa | 24.4%  /160 aa | 24.0%  /254 aa | 30.5%  /167 aa | 29.4%  /153 aa | **47.5%**  **/297 aa** |

**^a^** Solanum phureja Scaffold PGSC0003DMS000001253, 87804bp..86952bp

**^b^** Bold and underlined: significant identities over 40%

**^c^** Underlined: significant identity over 40% found only between short sequences
