# Supplementary material for: Widespread Endogenization of Genome Sequences of Non-Retroviral RNA Viruses into Plant Genomes
Source: PLoS Pathog. 2011 Jul 14;7(7):e1002146. doi: 10.1371/journal.ppat.1002146 (PMC3136472; doi:10.1371/journal.ppat.1002146)
Supplement: Table S3 — Oligonucleotide primers used in this study. (DOC) [file ppat.1002146.s009.doc]

**Table S3. Oligonucleotide primers used in this study.**

| **primer** | **Sequence (5’ – 3’)** | **Template** | **Used in** | **NRVS** |
| --- | --- | --- | --- | --- |
| At-IRS-FW | CCGTAGGTGAACCTCGGAGGG | ribosomal internal transcribed region (ITS) | Figures 1, 2, 3, 5, 7, S1, S3 as **ITS-F** | (ITS) |
| At-IRS-RV | GGTGATCCCGCCTGACCTGG | ribosomal internal transcribed region (ITS) | Figures 1, 2, 3, 5, 7, S1, S3 as **ITS-R** | (ITS) |
| ILR2up1412 | GTTCCAAGATGCRWATYACATTGGATACTT | ILR2 locus, upstream | Figure 1 as **At-1F** | AtPCLS1 |
| ILR2-univ-F3s | GYTACCAGCTCAAAAGCACAGYT | ILR2 locus, coding region | Figure 1 as **PC1-1** | AtPCLS1 |
| ILR2-univ-R2s | CTTGCTTSACMATCGATGGGATRGTGAGG | ILR2 locus, coding region | Figure 1 as **PC1-2** | AtPCLS1 |
| CASP-2F | TCTTCACTTGTCTCTACCGTATGTCTGCT | ILR2 locus, downstream flanking ORF | Figure 1 as **At-1R** | AtPCLS1 |
| AtPVCP2-dw | TATCTATCTGCCAACACTCTTCAGG | FCCV-CP(2) like, downstream (*Ar. thaliana* Col-0) | Figure 2 as **At-2** | AtPCLS2 |
| AtPVCP2-F | ATGATTTGCTACCCTACTCTCG | FCCV-CP(2) like, coding region (*Ar. thaliana* Col-0) | Figure 2 as **PC2-1** | AtPCLS2 |
| AtPVCP2-R | TTAAACGGCAGATTTCGAAACT | FCCV-CP(2) like, coding region (*Ar. thaliana* Col-0) | Figure 2 as **PC2-2** | AtPCLS2 |
| AlPVCP3-up | TTTTTCAGGGGTTTGGAACTTTGC | RSCV2-CP like, upstream (*Ar. lyrata*) | Figure 2 as **Al-3** | AlPCLS3 |
| AlPVCP3-F | ATGACGCACATCCTCCAGACTAA | RSCV2-CP like, coding region (*Ar. lyrata*) | Figure 2 as **PC3-1** | AlPCLS3 |
| AlPVCP3-R | TTAAAGTGGAGTGAGTCCGAAGTTT | RSCV2-CP like, coding region (*Ar. lyrata*) | Figure 2 as **PC3-2** | AlPCLS3 |
| CaCV-CP-likeNFw | TCCGTTCTTCTCCTTCCTGG | CaCV-CP like, coding region (*B. rapa*) | Figure 3 as **PC4-1** | BrPCLS4 |
| CaCV-CP-likeCRv | ACAGCACCGAGTCCGGTCGGG | CaCV-CP like, coding region (*B. rapa*) | Figure 3 as **PC4-2** | BrPCLS4 |
| Br-RSCVlike-F | CAAAACCTCTGGATCCCTGGTCCC | RSCV1-CP like, coding region (*B. rapa*) | Figure 3 as **PC5a-1** | BrPCLS5 |
| Br-RSCVlike-R | ACACGTCAGTGTCAGTGGCAGTGC | RSCV1-CP like, coding region (*B. rapa*) | Figure 3 as **PC5a-2** | BrPCLS5 |
| Sp-RSCVlike-F | CCCGGTTTGAAGCTAGAAGTTGTC | RSCV1-CP like, coding region (*Sol. phureja*) | Figure 3 as **PC5b-1** | SpPCLS5 |
| Sp-RSCVlike-R | AGACGGGAGATGAAGATACTGATG | RSCV1-CP like, coding region (*Sol. phureja*) | Figure 3 as **PC5b-2** | SpPCLS5 |
| PUX4-univ-R | AGTYCTTCYMACRCCCTGAAAKGAAAC | RSCV1-CP like, downstream flanking ORF PUX_4, (*Sol. phureja*) | Figure 3 as **Sp-5** | SpPCLS5 |
| Nt-Contig1-F | GTGGACTGACAACTGTTTGGGATG | RSCV1-CP like, coding region (*N. tabacum*) | Figure S1 as **PC5-1-1** | NtPCLS5-1 |
| Nt-Contig1-R | CAGCTAAGTTGAACTGGTAGGTGG | RSCV1-CP like, coding region (*N. tabacum*) | Figure S1 as **PC5-1-2** | NtPCLS5-1 |
| Nt-Contig2-F | AAATTCTCTGCGCTGTCTCTAGCC | RSCV1-CP like, coding region (*N. tabacum*) | Figure S1 as **PC5-2-1** | NtPCLS5-2 |
| Nt-Contig2-R | CTGGAAGCAGTTATGGTTACCGAC | RSCV1-CP like, coding region (*N. tabacum*) | Figure S1 as **PC5-2-2** | NtPCLS5-2 |
| Nt-Contig3-F | CTGCGTAACATTCACCGCACCTTC | FCCV-CP(3) like, coding region (*N. tabacum*) | Figure S1 as **PC6-1** | NtPCLS6 |
| Nt-Contig3-R | CATCTGTGCGATATGGCATGACAG | FCCV-CP(3) like, coding region (*N. tabacum*) | Figure S1 as **PC6-2** | NtPCLS6 |
| Nt-Contig4-F2 | GCATATGTTTCCTGCTGGTGTTATG | RSCV3-CP like, coding region (*N. tabacum*) | Figure S1 as **PC7a-1** | NtPCLS7 |
| Nt-Contig4-R2 | GATGTGGGCAGCAATGAGGTCAATTG | RSCV3-CP like, coding region (*N. tabacum*) | Figure S1 as **PC7a-2** | NtPCLS7 |
| Mt-Contig1-F | TGGATCATTCACCATTCGTGGACTC | RSCV3-CP like, coding region (*Med. truncatula*) | Figure S1 as **PC7b-1** | MtPCLS7 |
| Mt-Contig1-R | GGGTACGACATTGTTTGGATATTGC | RSCV3-CP like, coding region (*Med. truncatula*) | Figure S1 as **PC7b-2** | MtPCLS7 |
| LjPCLS8-F | TACCTCCGCGAGCTAAAAGACCGAA | RCV1-CP(3) like, coding region (*L. japonicus*) | Figure S1 as **PC8-1** | LjPCLS8 |
| LjPCLS8-R | CTAACATGAGCCGAAGCTCCATTCT | RCV1-CP(3) like, coding region (*L. japonicus*) | Figure S1 as **PC8-2** | LjPCLS8 |
| BrNSV-F | ATGGCAACATCTCCGAATGAACC | LBVaV CP-like, coding region (*B. rapa*) | Figure 5 as **RN1a-1** | BrRNLS1 |
| BrNSV-R | TTACTTCTTGGTTGATTCCTTCTC | LBVaV CP-like, coding region (*B. rapa*) | Figure 5 as **RN1a-2** | BrRNLS1 |
| AcRNLS-F1 | TAGTGTTGTTTTGAGTCTTGGAAGG | LBVaV CP-like, upstream (*Aq. coerulea*) | Figure S3 as **RN1b-1** | AqcRNLS1 |
| AcRNLS-R1 | TTGAATCCCACAATAGCTGGTCAGC | LBVaV CP-like, downstream (*Aq. coerulea* ) | Figure S3 as **RN1b-4** | AqcRNLS1 |
| Ac-NSV-ORF-F | ATGGGTACTTATCAATATCGCGTG | LBVaV CP-like, coding region (*Aq. coerulea* ) | Figure S3 as **RN1b-2** | AqcRNLS1 |
| Ac-NSV-ORF-R | GCACTTCATTTGAGTTTCAATCTGC | LBVaV CP-like, coding region (*Aq. coerulea* ) | Figure S3 as **RN1b-3** | AqcRNLS1 |
| appleRNLS-F1 | TTTCGGCAGTGGACTAGTCATTTAC | LBVaV CP-like, upstream (*Mal. domestica*) | Figure S3 as **RN1c-1** | MdRNLS1-1 |
| appleRNLS-R1 | TACGCAGGAACACGGAAGAGAAATC | LBVaV CP-like, downstream (*Mal. domestica*) | Figure S3 as **RN1c-2** | MdRNLS1-1 |
| appleRNLS2-F | ACCTTGAAGAGGGTCGAGGGATGG | LBVaV CP-like, coding region (*Mal. domestica*) | Figure S3 as **RN1d-1** | MdRNLS1-2 |
| appleRNLS2-R | ACGCCGGAACAAAACCAAGGTCATT | LBVaV CP-like, coding region (*Mal. domestica*) | Figure S3 as **RN1d-2** | MdRNLS1-2 |
| LjRNLS-F1 | CGCTTAGTCGTGATTAAGGCAGATG | LBVaV CP-like, coding region (*L. japonicus*) | Figure S3 as **RN1e-3** | LjRNLS1-1 |
| LjRNLS-F3 | AAGCTCTAGGTGCAGTGTACCCTT | LBVaV CP-like, upstream (*L. japonicus*) | Figure S3 as **RN1e-1** | LjRNLS1-1 |
| LjRNLS-R1 | CTATTGAATTAATTTGTTCCATGAC | LBVaV CP-like, downstream (*L. japonicus*) | Figure S3 as **RN1e-2** | LjRNLS1-1 |
| LjRNLS2-F | ATCGCGAATGCATTATGTTTCCTGG | LBVaV CP-like, coding region (*L. japonicus*) | Figure S3 as **RN1f-1** | LjRNLS1-2 |
| LjRNLS2-R | CTTGGCATATGCCATAGCAAACTTC | LBVaV CP-like, coding region (*L. japonicus*) | Figure S3 as **RN1f-1** | LjRNLS1-2 |
| CsRNLS-F | CATTAGCTTGGAACCTCCTTTCACC | LBVaV CP-like, coding region (*Cuc. sativus*) | Figure S3 as **RN1g-1** | CsRNLS1 |
| CsRNLS-R | GATCATTGCTAACAAACAGAATAAG | LBVaV CP-like, coding region (*Cuc. sativus*) | Figure S3 as **RN1g-1** | CsRNLS1 |
| NtNSV-F | ATGGCTGCAAATACCATAGATCTC | LBVaV CP-like, coding region (*N. tabacum*) | Figure 5 as **RN2-1** | NtRNLS2 |
| NtNSV-R | TCAGTTCTTCTTCAAAGATTCTCC | LBVaV CP-like, coding region (*N. tabacum*) | Figure 5 as **RN2-2** | NtRNLS2 |
| NtRNLS2-F | CTCAGGCATAGATGCAGATTGGATC | NSMV N-like, coding region (*N. tabacum*) | Figure S3 as **RN3-1** | NtRNLS3 |
| NtRNLS2-R | AATGATCAGAGCGAGCTCCTCAAGG | NSMV N-like, coding region (*N. tabacum*) | Figure S3 as **RN3-2** | NtRNLS3 |
| Cucsa.038520F | CTCCTTCACCAATCAACTCTAGAGC | CLBV rep-like, upstream flanking ORF (*Cuc. sativus*) | Figure 7 as **Cs-1F** | CsFRLS1 |
| Cucsa.038540R | CTATTCAACCGTCACAGGGACTCTC | CLBV rep-like, downstream flanking ORF (*Cuc. sativus*) | Figure 7 as **Cs-1R** | CsFRLS1 |
| Cs-DMV-mtf-F | TTCTGTTCGATCAAGGAATCCAGGTC | CLBV rep-like, Met domain (*Cuc. sativus*) | Figure 7 as **FR1-1** | CsFRLS1 |
| Cs-DMV-mtf-R | AATTTGTCTCAATTTAGCAAGGCCAC | CLBV rep-like, Met domain (*Cuc. sativus*) | Figure 7 as **FR1-2** | CsFRLS1 |
| Cs-DMV-intF | TCTCCAATTGGTTGCTTGATCAAGG | CLBV rep-like, Met-Hel inter region (*Cuc. sativus*) | Figure 7 as **FR1-3** | CsFRLS1 |
| Cs-DMV-intR | TTCATGGTAAACTCCTCAACCTCCC | CLBV rep-like, Met-Hel inter region (*Cuc. sativus*) | Figure 7 as **FR1-4** | CsFRLS1 |
| Cs-DMV-Hel-F | GGTTTCGCAGGTTCAGGGAAAAGTAG | CLBV rep-like, Hel domain (*Cuc. sativus*) | Figure 7 as **FR1-5** | CsFRLS1 |
| Cs-DMV-Hel-R | CAACCTTGATTCAACAAGTAGAACATC | CLBV rep-like, Hel domain (*Cuc. sativus*) | Figure 7 as **FR1-6** | CsFRLS1 |
| Cs-DMV-Hel-amp | GATGTTCTACTTGTTGAATCAAGGTTG | CLBV rep-like, Hel domain (*Cuc. sativus*) | Figure 7 as **FR1-6*** | CsFRLS1 |

Other primers used for analyzing plant genome-integrated NRVSs are available upon request.
