# Supplementary material for: Widespread Endogenization of Genome Sequences of Non-Retroviral RNA Viruses into Plant Genomes
Source: PLoS Pathog. 2011 Jul 14;7(7):e1002146. doi: 10.1371/journal.ppat.1002146 (PMC3136472; doi:10.1371/journal.ppat.1002146)
Supplement: Table S4 — Partitivirus CP-like sequences (PCLSs) analyzed in this study. (DOC) [file ppat.1002146.s010.doc]

**Table S4. Partitivirus CP-like sequences (PCLSs) analyzed in this study.**

| **HGT sequence** | **Plant** | **accession** | **Matched virus^a^** | **e-value** | **Current form of PCLS coding region^b^** | **Mol. analysis^c^** |
| --- | --- | --- | --- | --- | --- | --- |
| AtPCLS1 | *Arabidopsis thaliana* ecotype Col-0 | At3g18485**^d^** | RnPV2, (SsPV-S)^e^ | 2e-47 (1e-14)^e^ | disrupted by frame-shifting | GP, GS, SQ, PA |
| AtPCLS1(C24) | *Arabidopsis thaliana* ecotype C24 | AB576168 | RnPV2, (SsPV-S)^e^ | (2e-16)^e^ | disrupted by frame-shifting | GP, GS, SQ, PA |
| AtPCLS1(No-0) | *Arabidopsis thaliana* ecotype No-0 | AB576169 | RnPV2, (SsPV-S)^e^ | (2e-14)^e^ | disrupted by frame-shifting | GP, SQ, PA |
| AtPCLS1(Ler) | *Arabidopsis thaliana* ecotype Ler | AB576170 | RnPV2, (SsPV-S)^e^ | (NA^f^)^e^ | large truncation (5’-region) | GP, SQ |
| AtPCLS1(Ws) | *Arabidopsis thaliana* ecotype Ws | AY152649 | RnPV2, (SsPV-S)^e^ | 5e-12 (NA)^e^ | large truncation (5’-region) | GP, SQ |
| AtPCLS1(Sho) | *Arabidopsis thaliana* ecotype Shokei | AB609326 | RnPV2, (SsPV-S)^e^ | (2e-14)^e^ | disrupted by frame-shifting | GP, SQ, PA |
| AlPCLS1 | *Arabidopsis lyrata* | XM_002885214 | RnPV2, (SsPV-S)^e^ | 4e-2^g^ (NA)^e^ | large truncation (5’-region) | GP, SQ |
| AaPCLS1-A | *Arabidopsis arenosa* | AB609327 | RnPV2, (SsPV-S)^e^ | (8e-16)^e^ | ORF retained | GP, SQ, PA |
| AaPCLS1-B | *Arabidopsis arenosa* | AB609328 | RnPV2, (SsPV-S)^e^ | (4e-06)^e^ | disrupted by frame-shifting | GP, SQ, PA |
| CrPCLS1 | *Capsella rubella* | AB576171 | RnPV2, (SsPV-S)^e^ | (9e-18)^e^ | nearly entire ORF retained | GP, SQ, PA |
| CbPCLS1 | *Capsella bursa-pastoris* | AB576172 | RnPV2, (SsPV-S)^e^ | (1e-16)^e^ | nearly entire ORF retained | GP, GS, SQ, PA |
| TgPCLS1 | *Turritis glabra* | AB576173 | RnPV2, (SsPV-S)^e^ | (1e-03)^e,g^ | deletion (5’-region), frame-shifting , ORF extension**^h^** | GP, SQ |
| OkPCLS1-A | *Olimarabidopsis korshinskyi* | AB576175 | RnPV2, (SsPV-S)^e^ | (6e-14)^e^ | disrupted by internal stop codon | GP, SQ, PA |
| OkPCLS1-B | *Olimarabidopsis korshinskyi* | SAUR**^i^** | RnPV2, (SsPV-S)^e^ | NA | <partial sequence was obtained> | GP, SQ |
| OkPCLS1-C | *Olimarabidopsis korshinskyi* | AB609329 | RnPV2, (SsPV-S)^e^ | (7e-07)^e^ | partial deletion (3’-region) | GP, SQ, PA |
| OpPCLS1-A | *Olimarabidopsis pumila* | AB576174 | RnPV2, (SsPV-S)^e^ | (6e-14)^e^ | disrupted by internal stop codon | GP, SQ, PA |
| OpPCLS1-B | *Olimarabidopsis pumila* | SAUR | RnPV2, (SsPV-S)^e^ | NA | <partial sequence was obtained> | GP, SQ |
| OpPCLS1-C | *Olimarabidopsis pumila* | SAUR | RnPV2, (SsPV-S)^e^ | NA | <partial sequence was obtained>, partial deletion (3’-region) | GP, SQ |
| AtPCLS2 | *Arabidopsis thaliana* ecotype Col-0 | At4g14104 | RSCV2, dsRNA2 | 3e-49 | truncated from 5’- and 3’-region but in-frame | GP, SQ |
| AtPCLS2(Ler) | *Arabidopsis thaliana* ecotype Ler | NA | NA | NA | NA | GP |
| AtPCLS2(Sho) | *Arabidopsis thaliana* ecotype Shokei | SAUR | RSCV2, dsRNA2 | <4e-47>^j^ | <partial sequence was obtained> | GP, SQ |
| AlPCLS3 | *Arabidopsis lyrata* | XM_002872767 | FCCV, dsRNA 2 | 5e-38 | truncated from 5’- and 3’-region but in-frame | GP, SQ |
| AaPCLS3 | *Arabidopsis arenosa* | SAUR | FCCV, dsRNA 2 | <6e-38> ^j^ | <partial sequence was obtained> | GP, SQ |
| BrPCLS4(Cc) | *Brassica rapa* (Chinese cabbage) | AB609330 | CaCV1 | 6e-107 | full viral sequence but frame-shifted | GP, GS, SQ, PA |
| BrPCLS4(tu) | *Brassica rapa* (turnip) | NA | NA | NA | NA | GP |
| BnPCLS4 | *Brassica napus* | AB609331 | CaCV1 | 5e-98 | full viral sequence, internal stop codon, frame-shifting | GP, SQ, PA |
| BoPCLS4(ca) | *Brassica oleracea* (cabbage) | AB609332 | CaCV1 | 1e-100 | disrupted by frame-shifting | GP, GS, SQ, PA |
| BoPCLS4(br) | *Brassica oleracea* (broccoli) | NA | NA | NA | NA | GP, GS |
| BrPCLS5(Cc) | *Brassica rapa* (Chinese cabbage) | AB609333 | RSCV1 | 2e-130 | partially truncated from 5’- and 3’-region but in-frame | GP, GS, SQ, PA |
| BrPCLS5(tu) | *Brassica rapa* (turnip) | AB609334 | RSCV1 | 2e-130 | partially truncated from 5’- and 3’-region but in-frame | GP, SQ, PA |
| BnPCLS5 | *Brassica napus* | AB609335 | RSCV1 | 3e-124 | partially truncated from 5’- and 3’-region, internal stop codon | GP, SQ, PA |
| BoPCLS5(ca) | *Brassica oleracea* (cabbage) | AB609336 | RSCV1 | 2e-82 | partially truncated from 5’- and 3’-region, internal stop codon | GP, GS, SQ, PA |
| BoPCLS5(br) | *Brassica oleracea* (broccoli) | NA | NA | NA | NA | GP, GS |
| StPCLS5 | *Solanum tuberosum* | AB609337 | RSCV1 | 4e-111 | disrupted by frame-shifting and internal stop codon | GP, GS, SQ, PA |
| SlPCLS5 | *Solanum lycopersicum* | AB609338 | RSCV1 | 2e-73 | disrupted by frame-shifting and internal stop codon | GP, GS, SQ, PA |
| NtPCLS5-1 | *Nicotiana tabacum* | SAUR | RSCV1 | <2e-69> ^j^ | <partial sequence was obtained> | GP, GS, SQ, PA |
| NtPCLS5-2 | *Nicotiana tabacum* | SAUR | RSCV1 | <4e-28> ^j^ | <partial sequence was obtained> | GP, GS, SQ |
| NtPCLS6 | *Nicotiana tabacum* | SAUR | FCCV, dsRNA3 | <1e-26> ^j^ | <partial sequence was obtained> | GP, GS, SQ |
| NmPCLS6 | *Nicotiana megalosiphon* | SAUR | FCCV, dsRNA3 | <1e-19> ^j^ | <partial sequence was obtained> | GP, SQ |
| NtPCLS7 | *Nicotiana tabacum* | SAUR | RSCV3 | <1e-03> ^j^ | <partial sequence was obtained> | GP, GS, SQ |
| NbPCLS7 | *Nicotiana benthamiana* | SAUR | RSCV3 | <7e-21> ^j^ | <partial sequence was obtained> | GP, GS, SQ |
| NmPCLS7 | *Nicotiana megalosiphon* | SAUR | RSCV3 | <3e-27> ^j^ | <partial sequence was obtained> | GP, SQ |
| NsPCLS7 | *Nicotiana sanderae* | SAUR | RSCV3 | <3e-20> ^j^ | <partial sequence was obtained> | GP, SQ |
| MtPCLS7(A17) | *Medicago truncatula* line A17 | SAUR | RSCV3 | <6e-22> ^j^ | truncated terminus, internal stop codons, frame-shifting | GP, SQ |
| MtPCLS7(A20) | *Medicago truncatula* line A20 | SAUR | RSCV3 | <9e-21> ^j^ | truncated terminus, internal stop codons, frame-shifting | GP, SQ |
| MtPCLS7(R108) | *Medicago truncatula* line R108 | SAUR | RSCV3 | <9e-21> ^j^ | truncated terminus, internal stop codons, frame-shifting | GP, SQ |
| LjPCLS8(B) | *Lotus japonicus* line B129 | SAUR | RCV1, dsRNA 3 | <1e-29> ^j^ | <partial sequence was obtained>  truncated terminus, large fragment insertion | GP, SQ |
| LjPCLS8(M) | *Lotus japonicus* line MG-20 | SAUR | RCV1, dsRNA 3 | <2e-31> ^j^ | <partial sequence was obtained>  truncated terminus, large fragment insertion | GP, SQ |

**^a^** See Table 1 for abbreviation of the virus names.

**^b^** In-frame small deletions and insertions, and nucleotide substitutions, frequently found, are not indicated in this table.

**^c^** Molecular analysis carried out in this study: GP, genomic PCR; GS, genomic Southern blot; SQ, sequencing; PA, phylogenetic analysis; -, not performed.

**^d^** The accession of ILR2 putative mRNA sequence lacking predicted intron sequence (100 bps) is NM_148731.

**^e^** E-value is shown against Sclerotinia sclerotiorum partitivirus S (SsPV-S) CP sequence rather than the RnPV2 CP sequence that is unavailable for BLAST database search at the time of submission.

**^f^** NA: not applicable.

**^g^** Lower e-value could be obtained when compared with AtILR2.

**^h^** ORF extension: loss of original stop codon of ILR2 extends the reading frame (Figure1 A, *T. glabra*, gray box).

**^i^** SAUR: sequence available upon request.

**^j^** E-values obtained using partial sequences as queries. Because the length of query sequences is different among homologues, e-values even for some similar sequences vary to a great extent.
