# Supplementary material for: Widespread Endogenization of Genome Sequences of Non-Retroviral RNA Viruses into Plant Genomes
Source: PLoS Pathog. 2011 Jul 14;7(7):e1002146. doi: 10.1371/journal.ppat.1002146 (PMC3136472; doi:10.1371/journal.ppat.1002146)
Supplement: Table S5 — Rhabdovirus N-like sequences (RNLSs) analyzed in this study. (DOC) [file ppat.1002146.s011.doc]

**Table S5. Rhabdovirus N-like sequences (RNLSs) analyzed in this study.**

| **HGT sequence** | **Plant** | **accession** | **Matched virus** | **e-value** | **Current form of RNLS coding region^a^** | **Mol. analysis^b^** |
| --- | --- | --- | --- | --- | --- | --- |
| BrRNLS1A(Cc) | *Brassica rapa* (Chinese cabbage) | AB609339 | lettuce big-vein associated virus | 7e-09 | Complete N ORF | GP, GS, SQ, PA |
| BrRNLS1B(Cc) | *Brassica rapa* (Chinese cabbage) | AB609340 | lettuce big-vein associated virus | 1e-09 | Complete N ORF | GP, GS, SQ, PA |
| BrRNLS1(tu) | *Brassica rapa* (turnip) | NA**^c^** | lettuce big-vein associated virus | NA | NA | GP |
| BnRNLS1A | *Brassica napus* | AB609341 | lettuce big-vein associated virus | 4e-08 | Complete N ORF | GP, SQ, PA |
| BnRNLS1B | *Brassica napus* | AB609342 | lettuce big-vein associated virus | 3e-9 | Complete N ORF | GP, SQ, PA |
| BoRNLS1A(ca) | *Brassica oleracea* (cabbage) | AB609343 | lettuce big-vein associated virus | 7e-09 | Complete N ORF | GP, GS, SQ, PA |
| BoRNLS1(br) | *Brassica oleracea* (broccoli) | NA | lettuce big-vein associated virus | NA | NA | GP, GS |
| RsRNLS1A | *Raphanus sativus* | AB609344 | lettuce big-vein associated virus | 3e-11 | Complete N ORF (terminal sequences are primer origin) | GP, SQ, PA |
| RsRNLS1B | *Raphanus sativus* | AB609345 | lettuce big-vein associated virus | 5e-12 | Complete N ORF (terminal sequences are primer origin) | GP, SQ, PA |
| AqfRNLS1 | *Aquilegia flabellata* var. *pumila* | AB609346 | lettuce big-vein associated virus | 6e-17 | Complete N ORF | GP, SQ, PA |
| MdRNLS1-1(F) | *Malus x domestica* cv. Sun-Fuji | AB609347 | lettuce big-vein associated virus | 3e-31 | Complete N ORF | GP, SQ, PA |
| MdRNLS1-1(J) | *Malus x domestica* cv*.* Jonagold | SAUR**^d^** | lettuce big-vein associated virus | <1e-03>^e^ | <partial sequence was obtained> multicopy**^f^** | GP, SQ |
| MdRNLS1-1(M) | *Malus x domestica* cv. Mutsu | SAUR | lettuce big-vein associated virus | <1e-03>^e^ | <partial sequence was obtained> multicopy | GP, SQ |
| MdRNLS1-1(O) | *Malus x domestica* cv. Ohrin | SAUR | lettuce big-vein associated virus | <8e-29>^e^ | <partial sequence was obtained> multicopy | GP, SQ |
| MdRNLS1-2(F) | *Malus x domestica* cv. Sun-Fuji | SAUR | lettuce big-vein associated virus | <7e-10>^e^ | <partial sequence was obtained> | GP, SQ |
| MdRNLS1-2(J) | *Malus x domestica* cv. Jonagold | SAUR | lettuce big-vein associated virus | <7e-10>^e^ | <partial sequence was obtained> | GP, SQ |
| MdRNLS1-2(M) | *Malus x domestica* cv. Mutsu | SAUR | lettuce big-vein associated virus | <7e-10>^e^ | <partial sequence was obtained> | GP, SQ |
| MdRNLS1-2(O) | *Malus x domestica* cv. Ohrin | SAUR | lettuce big-vein associated virus | <7e-10>^e^ | <partial sequence was obtained> | GP, SQ |
| LjRNLS1-1(B) | *Lotus japonicus* line B129 | AB609348 | lettuce big-vein associated virus | 3e-13 | Complete N ORF | GP, SQ, PA |
| LjRNLS1-1(M) | *Lotus japonicus* line MG-20 | AB609349 | lettuce big-vein associated virus | 3e-13 | Complete N ORF | GP, SQ, PA |
| LjRNLS1-2(B) | *Lotus japonicus* line B129 | SAUR | lettuce big-vein associated virus | <2e-15>^e^ | <partial sequence was obtained> | GP, SQ |
| LjRNLS1-2(M) | *Lotus japonicus* line MG-20 | SAUR | lettuce big-vein associated virus | <9e-15>^e^ | <partial sequence was obtained> | GP, SQ |
| CsRNLS1(H) | *Cucumis sativus* cv. Hokushin | SAUR | lettuce big-vein associated virus | <4e-10>^e^ | <partial sequence was obtained> | GP, SQ |
| CsRNLS1(S) | *Cucumis sativus* cv. Suyo | SAUR | lettuce big-vein associated virus | <1e-10>^e^ | <partial sequence was obtained> | GP, SQ |
| CsRNLS1(B) | *Cucumis sativus*  cv. Borszczagowski B10 line | SAUR | lettuce big-vein associated virus | <3e-10>^e^ | <partial sequence was obtained> | GP, SQ |
| NtRNLS2 | *Nicotiana tabacum* | AB609350 | lettuce necrotic yellows virus | 5e-35 | Complete N ORF | GP, GS, SQ, PA |
| NtRNLS3 | *Nicotiana tabacum* | SAUR | northern cereal mosaic virus | <9e-10>^e^ | <partial sequence was obtained> | GP, SQ |

**^a^** In-frame small deletions and insertions, and nucleotide substitutions, frequently found, are not indicated in this table.

**^b^** Molecular analysis carried out in this study: GP, genomic PCR; GS, genomic Southern blot; SQ, sequencing; PA, phylogenetic analysis; -, not performed.

**^c^** NA: not applicable.

**^d^** SAUR: sequence available upon request.

**^e^** E-values obtained using partial sequences as queries. Because the length of query sequences is different among homologues, e-values even for some similar sequences vary to a great extent.

**^f^** Direct sequencing of PCR fragments showed heterogeneity at some positions.
