# Supplementary material for: Widespread Endogenization of Genome Sequences of Non-Retroviral RNA Viruses into Plant Genomes
Source: PLoS Pathog. 2011 Jul 14;7(7):e1002146. doi: 10.1371/journal.ppat.1002146 (PMC3136472; doi:10.1371/journal.ppat.1002146)
Supplement: Table S6 — Rhabdovirus N-like sequences (RNLSs) identified in plant EST collections. (DOC) [file ppat.1002146.s012.doc]

**Table S6. Rhabdovirus N-like sequences (RNLSs) found in plant EST collections.**

| **EST-Contig^a^** | **Plant** | **Database^b^** | **Best-matched virus** | **e-value^c^** |
| --- | --- | --- | --- | --- |
| Festuca EST | *Festuca pratensis* | NCBI- nr | lettuce big-vein associated virus | 6e-25 |
| Aquilegia EST | *Aquilegia formosa x Aquilegia pubescens* | NCBI- nr | lettuce big-vein associated virus | 2e-20 |
| B. oleracea EST | *Brassica oleracea var. acephala* (kale) | NCBI- nr | lettuce big-vein associated virus | 9e-12 |
| B. napus EST | *Brassica napus* | NCBI- nr | lettuce big-vein associated virus | 4e-11 |
| Cichorium EST | *Cichorium intybus* (chicory) | NCBI- nr | northern cereal mosaic virus | 2e-10 |
| Picea EST | *Picea glauca* (white spruce) | NCBI- nr | lettuce necrotic yellows virus | 2e-15 |
| Triphysaria EST | *Triphysaria pusilla* | NCBI- nr | lettuce necrotic yellows virus | 2e-65 |

**^a^** The contig-map of these assembled EST sequences are illustrated in Figure S4.

**^b^** Non-mouse and non-human EST entries.

**^c^** TBLAST e-value for the top hits from the NCBI Non-redundant protein sequences (nr) database.
