# Supplementary material for: Widespread Endogenization of Genome Sequences of Non-Retroviral RNA Viruses into Plant Genomes
Source: PLoS Pathog. 2011 Jul 14;7(7):e1002146. doi: 10.1371/journal.ppat.1002146 (PMC3136472; doi:10.1371/journal.ppat.1002146)
Supplement: Table S7 — Amino acid sequence identities among selected rhabdovirus Ns/CPs and plant rhabdovirus N-like sequences (RNLSs). (DOC) [file ppat.1002146.s013.doc]

**Table S7. Amino acid sequence identities among selected rhabdovirus Ns/CPs and plant rhabdovirus N-like sequences (RNLSs).**

| **Seqence name** | **Plant or virus name**  **(locus or accession)** | **Sequence Size (aa)** | **LBVaV CP**  (BAD36830) | **BrRNLS1** | **AqcRNLS1** | **MdRNLS1-1** | **LjRNLS1-1** | **CsRNLS1** | **MgRNLS1** | **LNYV N** | **NtPCLS4** | **LYMoV N** |
| --- | --- | --- | --- | --- | --- | --- | --- | --- | --- | --- | --- | --- |
| **BrRNLS1** | *Brassica rapa* (Bra020820) | 406 | 18.6%  /354 aa |  |  |  |  |  |  |  |  |  |
| **AqcRNLS1** | *Aquilegia coerulea* (AcoGold_v1.007196m) | 364 | 21.2%  /339 aa | 25.2%  /345 aa |  |  |  |  |  |  |  |  |
| **MdRNLS1-1** | *Malus x domestica* (unassigned) | 366 | 27.8%  /370 aa | 25.8%  /299 aa | 24.6%  /346 aa |  |  |  |  |  |  |  |
| **LjRNLS1-1** | *Lotus japonicus* (unassigned) | 412 | 21.5%  /335 aa | 25.3%  /229 aa | 25.8%  /349 aa | 22.5%  /346 aa |  |  |  |  |  |  |
| **CsRNLS1** | *Cucumis sativus* (unassigned) | 486 | 23.7%  /308 aa | 29.2%  /209 aa | 25.4%  /311 aa | 28.9%  /342 aa | 26.3%  /186 aa |  |  |  |  |  |
| **MgRNLS1** | *Mimulus guttatus* ( mgf014425m ) | 415 | 18.3%  /349 aa | 21.9%  /311 aa | 29.5%  /336 aa | 20.8%  /355 aa | 26.9%  /338 aa | 20.5%  /278 aa |  |  |  |  |
| **LNYV N** | LNYV  (CAI30421) | 459 | 22.3%  /130 aa | 19.6%  /224 aa | 21.9%  /260 aa | 24.8%  /218 aa | 24.8%  /230 aa | 20.3%  /202 aa | 22.0%  /218 aa |  |  |  |
| **NtRNLS2** | *Nicotiana tabacum* (GSS Contig-5) | 408 | 19.4%  /289 aa | 22.4%  /183 aa | 17.8%  /338 aa | 19.7%  /229 aa | 24.2%  /244 aa | 19.5%  /185 aa | 20.7%  /222 aa | 27.4%  /379 aa |  |  |
| **LYMoV N** | LYMoV  (ABV56124 ) | 452 | 18.7%  /241 aa | 18.2%  /231 aa | 21.0%  / 267 aa | 22.5%  /293 aa | 21.8%  /238 aa | 17.1%  /245 aa | 18.4%  /223 aa | **57.3%**  **/450 aa^a^** | 24.9%  /385 aa |  |
| **PtRNLS4** | *Populus trichocarpa* ( POPTR_0008s16330 ) | 466 | 28.6%  /84 aa | 21.5%  /223 aa | 23.7%  /224 aa | 22.8%  /189 aa | 17.4%  /218 aa | 34.5%  /55 aa^‡^ | 19.5%  /374 aa | 29.0%  /427 aa | 21.0%  /400 aa | 28.9%  /433 aa |

^a^ Bold and underlined: significant identities over 40%
